# Imaging Mass Spectrometry and Lectin Analysis of N-Linked Glycans in Carbohydrate Antigen–Defined Pancreatic Cancer Tissues

**DOI:** 10.1074/mcp.RA120.002256

**Published:** 2020-12-08

**Authors:** Colin T. McDowell, Zachary Klamer, Johnathan Hall, Connor A. West, Luke Wisniewski, Thomas W. Powers, Peggi M. Angel, Anand S. Mehta, David N. Lewin, Brian B. Haab, Richard R. Drake

**Affiliations:** 1 Department of Cell and Molecular Pharmacology and Experimental Therapeutics, Medical University of South Carolina, Charleston, South Carolina, USA; 2 Van Andel Research Institute, Grand Rapids, Michigan, USA; 3 Department of Pathology and Laboratory Medicine, Medical University of South Carolina, Charleston, South Carolina, USA

**Keywords:** imaging mass spectrometry, N-glycans, MALDI, pancreatic cancer, CA19-9, lectins, biomarkers, glycosylation, BSA, bovine serum albumin, CA19-9, carbohydrate antigen 19-9, CHCA, alpha-cyano-4-hydroxycinnamic acid, DAB, 3,3′-diaminobenzidine, DMSO, dimethyl sulfoxide, Endo F3, endoglycosidase F3, EtOH, ethanol, FC, fold change, FFPE, formalin-fixed paraffin-embedded, FTICR, Fourier-transform ion cyclotron resonance, GSL-II, *Griffonia simplicifolia* lectin II, HexNAc, N-acetylhexosamine, HRP, horseradish peroxidase, IHC, immunohistochemistry, IMS, imaging mass spectrometry, LacNAc, N-acetyl-lactosamine, LASSO, least absolute shrinkage and selection operator, NHS, N-hydroxysuccinimide, PBST0.05, PBS with 0.05% Tween-20, PDAC, pancreatic ductal adenocarcinoma, PHA-E, phaseolus vulgaris erythroagglutinin, PNGase F, peptide N-glycosidase F, QTOF, quadrupole time-of-flight, sLeA, sialyl-Lewis A, sTRA, sialylated tumor-related antigen, TMA, tissue microarray

## Abstract

The early detection of pancreatic ductal adenocarcinoma (PDAC) is a complex clinical obstacle yet is key to improving the overall likelihood of patient survival. Current and prospective carbohydrate biomarkers carbohydrate antigen 19-9 (CA19-9) and sialylated tumor-related antigen (sTRA) are sufficient for surveilling disease progression yet are not approved for delineating PDAC from other abdominal cancers and noncancerous pancreatic pathologies. To further understand these glycan epitopes, an imaging mass spectrometry (IMS) approach was used to assess the N-glycome of the human pancreas and pancreatic cancer in a cohort of patients with PDAC represented by tissue microarrays and whole-tissue sections. Orthogonally, these same tissues were characterized by multiround immunofluorescence that defined expression of CA19-9 and sTRA as well as other lectins toward carbohydrate epitopes with the potential to improve PDAC diagnosis. These analyses revealed distinct differences not only in N-glycan spatial localization across both healthy and diseased tissues but importantly between different biomarker-categorized tissue samples. Unique sulfated biantennary N-glycans were detected specifically in normal pancreatic islets. N-glycans from CA19-9–expressing tissues tended to be biantennary, triantennary, and tetra-antennary structures with both core and terminal fucose residues and bisecting GlcNAc. These N-glycans were detected in less abundance in sTRA-expressing tumor tissues, which favored triantennary and tetra-antennary structures with polylactosamine extensions. Increased sialylation of N-glycans was detected in all tumor tissues. A candidate new biomarker derived from IMS was further explored by fluorescence staining with selected lectins on the same tissues. The lectins confirmed the expression of the epitopes in cancer cells and revealed different tumor-associated staining patterns between glycans with bisecting GlcNAc and those with terminal GlcNAc. Thus, the combination of lectin-immunohistochemistry and lectin-IMS techniques produces more complete information for tumor classification than the individual analyses alone. These findings potentiate the development of early assessment technologies to rapidly and specifically identify PDAC in the clinic that may directly impact patient outcomes.

## Highlights

• N-glycan structures localize to distinct regions in normal and PDAC tumor tissue.
• N-glycan composition differs between biomarker-stratified PDAC subtypes.
• Modeling with N-glycan IMS and biomarker data improves tumor/normal discrimination.
• Multiplexed IMS and lectin staining potentiates new PDAC classification strategies.

## Graphical Abstract

**Figure:**
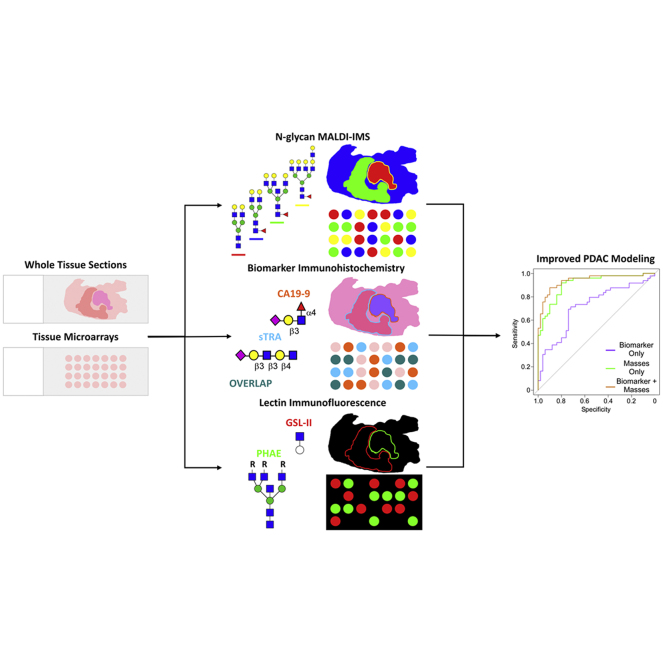

## In Brief

N-glycosylation is an attractive target for PDAC biomarker discovery because of its well-understood roles in oncogenesis, cancer maintenance, and metastasis. Using MALDI-IMS, we observed distinct histopathology localized differences in N-glycosylation distributions between healthy and cancerous tissues. We combined the tumor-to-normal ratio of N-glycan changes determined from IMS with biomarker IHC data into modeling which improved PDAC identification over models utilizing either data set individually. This multiplexed approach potentiates the development of cross-disciplinary biomarker panels for pancreatic cancer detection.

Pancreatic ductal adenocarcinomas (PDACs) are aggressive neoplasia diagnosed in approximately 57,000 patients in the United States, with nearly 46,000 recorded deaths during the year 2019, and both metrics are predicted to continue to increase. PDAC accounts for 7% of total cancer deaths and has the lowest 5-year survival rate of any cancer at just 9% for all stages combined (1, 2). The high mortality associated with pancreatic cancer is due to late detection of the disease as compared with other cancers, with 22% of patients diagnosed at the locally invasive stage, 13% at the locally advanced stage, and 55% at the metastatic stage (3). The typically late identification of PDAC owes to the heterogeneity of often-mild early-stage symptoms, the location of the pancreas deep within the abdominal cavity, and the lack of reliable biomarkers for early identification. Currently, the sole Food and Drug Administration-approved biomarker for pancreatic cancer is carbohydrate antigen 19-9 (CA19-9), increased levels of which are associated with the majority of PDAC cases. Despite CA19-9's utility as a prognostic indicator for judging the effectiveness of a patient's treatment regimen, the antigen's association with other cancers and noncancer malignancies such as cystic fibrosis, pancreatitis, and liver disease make it unsuitable as a diagnostic predictor for pancreatic cancer (4). The glycan epitope of the CA19-9 antibody appears on the surface of cancer cells in about 60% of PDACs. Another glycan marker called sialylated tumor-related antigen (sTRA), recently discovered as a biomarker of pancreatic cancer, likewise appears in the majority of tumors but in a nonidentical subgroup of tumors (5). Some tumors have only one of the glycans, some have both, and some have neither. Together, these two antigens are in development for clinical use as a means of pancreatic cancer surveillance and differentiation between pancreatic cancer and other pancreatic diseases. Because CA19-9 and sTRA only recognize sialyl-Lewis A (sLeA) and a desialylated type 1 and type 2 N-acetyl-lactosamine (LacNAc) carbohydrate motifs, respectively, an important aspect of better understanding their ability to recognize PDAC is the elucidation of specifically which glycans or glycoproteins these antibodies are binding to and what role these molecules play in the biological processes underlying the development of pancreatic cancer.

An important and yet often understudied aspect of cancer biology is the glycocalyx, the dense layer of complex carbohydrates that plays a significant role in governing the interactions between cancer cells, immune cells, and the local microenvironment. The glycocalyx is composed of N- and O-linked glycans attached to a wide variety of glycoproteins, glycosaminoglycans attached to proteoglycans, and glycolipids. The majority of human proteins and lipids have glycan modifications, which play key roles in protein folding, cell–cell communication, intracellular trafficking, and signal transduction among other functions (6). Consequently, aberrations in glycosylation state often result in or are indicators of disease, and many glycoproteins or glycans are known biomarkers of cancer. Indeed, it has been well studied that changes in glycan structure and composition can alter the metastatic capacity and the invasive spread of cancer cells (7). Of specific interest to pancreatic cancer is N-linked glycosylation (attached to the asparagine residue in Asn-X-Ser/Thr protein motifs), which is known to play a role in immune recognition, cellular mobility, and cell signaling. Increases in N-glycan branching, fucosylation, and sialylation have also recently been associated with PDAC (8).

In an attempt to better define the N-linked glycome of pancreatic cancer directly in clinical tissues, we have used MALDI–imaging mass spectrometry (MALDI–IMS) to identify peptide N-glycosidase–released N-glycans linked spatially and histochemically to pathology features. This method has been used to map N-glycan distribution across multiple cancer types using formalin-fixed paraffin-embedded (FFPE) tissue samples (7, 9, 10, 11, 12, 13, 14, 15, 16, 17, 18). When complemented with histopathological identification, this combined analysis can map correlations between specific glycans or groups of glycans and different tissue regions and subtypes including tumor, necrotic, and adjacent normal tissues (10). The present study analyzes the N-glycome of the pancreas and PDAC. A majority of previous studies on the role of glycosylation in pancreatic cancer have focused on serum profiling of N-glycans and the truncation of O-linked glycosylation and increased O-GlcNAcylation (8, 19). Comprehensive spatial mapping of N-glycan distribution in normal and pancreatic cancer tissues is lacking, and correlative mapping of N-glycans to immunohistochemistry (IHC) staining of current and prospective pancreatic cancer biomarkers has only recently begun to be explored (20). The present study addresses this using an MALDI–IMS approach to characterize the N-glycans present in healthy pancreatic tissue and a cohort of pancreatic cancer tissues represented by multiple tissue microarrays (TMAs) and a subset of the source whole-block tissue slices. Changes in tissue glycans associated with PDAC were further correlated with antibody-based assessments for CA19-9, sTRA, and other carbohydrate epitopes in the same tissues. The cumulative analyses revealed multiple subsets of N-glycans with increased branching, bisecting GlcNAc, fucosylation, and sialylation in PDAC-specific regions of tissues, many of which could be correlated with each carbohydrate antigen biomarker.

## Experimental Procedures

HPLC-grade acetonitrile, ethanol (EtOH), methanol, xylene, and water as well as CitriSolv Hybrid were obtained from Fisher Scientific. 28 to 30% ammonia in water, bovine serum albumin (BSA), dimethyl sulfoxide (DMSO), citraconic anhydride, and 1 X PBS were obtained from Thermo Scientific. Acetic acid, alpha-cyano-4-hydroxycinnamic acid (CHCA), chloroform, 4′,6-diamidino-2-phenylindole, dimethylamine, hydrogen peroxide, 1-hydroxybenzotriazole hydrate, sodium bicarbonate, TFA, Triton-X100, and Tween-20 were obtained from Sigma-Aldrich. 1-(3-Dimethylaminopropyl)-3-ethylcarbodiimide was obtained from Alfa Aesar. Recombinant peptide N-glycosidase F (PNGase F) PRIME was obtained from N-Zyme Scientifics, and recombinant endoglycosidase F3 (Endo F3) was obtained from the laboratory of Dr Anand Mehta as described (11, 18). H&E stains were obtained from Cancer Diagnostics. Horseradish peroxidase–3,3′-diaminobenzidine (HRP–DAB) staining kits were obtained from R&D Systems. CA19-9 mAb was obtained from USBiological. TRA-1-60 mAb was obtained from Novus Biologicals. Sulfo-cyanine3 N-hydroxysuccinimide (NHS) and sulfo-cyanine5 NHS esters were obtained from Lumiprobe. Tetramethylrhodamine-conjugated anti–phaseolus vulgaris erythroagglutinin (PHA-E), biotinylated *Griffonia simplicifolia* lectin II (GSL-II), and Cy5-conjugated streptavidin were obtained from EY Laboratories, Vector Laboratories, and Roche Applied Science, respectively. α2-3,6,8 Neuraminidase and 1 X Glycobuffer were obtained from New England Biolabs.

### Clinical Pancreatic Cancer FFPE Tissues

Four FFPE pancreatic cancer TMAs (n = 53 patients) were provided by the Medical University of South Carolina's Hollings Cancer Center Biorepository and Tissue Analysis Shared Resource in accordance with the National Cancer Institute's Best Practices for Biospecimen Resources. Fifty-one of 53 cases of the pancreatic cancer cohort were adenocarcinomas, with the remaining 2 of 53 being cholangiocarcinoma and adenosquamous carcinoma. Each case was represented by a minimum of 4 cores, 2 from adjacent tissue and 2 from tumor tissue. Furthermore, 23 of 53 cases also had representative pretumor tissue, 29 of 53 had metastatic tissue, 22 of 53 had lymph-node tissue, and 14 of 53 had lymph-node tumor tissue. The staging of the cases ranged from T1NOMX (2 cm or smaller tumor localized to the pancreas, absent from regional lymph nodes) to T4N1MX (4 cm or larger tumor extending into local major arteries, spread to 1–3 regional lymph nodes), with most cases staged at T3N1MX (4 cm or larger tumor extending beyond the pancreas, spread to 1–3 regional lymph nodes) (3). The majority of the patients' tumors were moderately to poorly differentiated. Thirteen of 53 patients experienced recurrence, mostly at distant sites such as the lungs and liver. More detailed information is contained in supplemental Table S1. Full tissue slices of thirteen PDAC pathology blocks that were the original sources of the tumor cores in the TMAs mentioned above were also analyzed. All cases were adenocarcinomas, the majority of which were staged at T3N1MX. Normal human pancreas FFPE tissue slides were obtained from Zyagen.

### Tissue Preparation for MALDI–IMS

A standardized protocol was used for tissue preparation, enzymatic digestion, and matrix application by a solvent sprayer (9). Tissue slides were dewaxed in xylenes and then rehydrated in a gradation of EtOH and water washes before antigen retrieval with citraconic anhydride buffer (25-μl citraconic anhydride, 2-μl 12 M HCl, 50-ml HPLC-grade water, pH 3.0 ± 0.5) for 30 min in a decloaking chamber at 95 °C. Fifteen passes of PNGase F or 10% Endo F3/90% PNGase F at 0.1 μg/μl was then sprayed onto the slides at a rate of 25 μl/min with a 3-mm offset and a velocity of 1200 at 45 °C and 10 psi using an M5 TM Sprayer (HTX Technologies LLC). Tissue slides were then incubated at 37 °C for 2 h in prewarmed humidity chambers followed by desiccation before matrix application. 10 passes of 7 mg/ml CHCA matrix in 50% acetonitrile/0.1% TFA was applied to the desiccated slides at 0.1 ml/min with a 2.5-mm offset and a velocity of 1300 at 80 °C and 10 psi using the TM Sprayer. After matrix application slides were desiccated until analysis by MALDI–Fourier-transform ion cyclotron resonance (FTICR) MS or MALDI quadrupole time-of-flight mass spectrometry (MALDI–QTOF MS). Postanalysis whole-tissue slides were H&E-stained according to a standardized protocol and annotated for histological classification by an expert pathologist.

### Sialic Acid Derivatization by Amidation–Amidation Reaction

Sialic acids were stabilized by chemical amidation directly on tissue using an established protocol (21), with some slight modifications as follows. Tissue slides were dewaxed and rehydrated as described above and then incubated with 200 μl of the primary reaction solution (22-μl 1-(3-Dimethylaminopropyl)-3-ethylcarbodiimide, 42.2-mg 1-hydroxybenzotriazole hydrate, 15.8-μl dimethylamine, 0.5-ml DMSO) for 1 h at 60 °C. After incubation, tissues were washed with 200-μl DMSO 3× and then incubated with 200 μl of the secondary reaction solution (150-μl 28–30% ammonia in water and 350-μl DMSO) for 2 h at 60 °C. After the second incubation, slides were rinsed sequentially in 100% EtOH for 2 min 2×, Carnoy's solution (30% chloroform, 60% EtOH, 10% acetic acid) for 10 min 2×, water for 1 min, 100% EtOH for 2 min 2×, and 0.1% TFA in EtOH for 30 s. Slides were then immediately processed for MALDI–IMS as described above, starting with antigen retrieval.

### MALDI–FTICR Analysis of N-Glycans

A Solarix dual-source 7T FTICR mass spectrometer (Bruker Daltonik) (*m/z* 490–5000) equipped with a 2000-Hz SmartBeam II laser utilizing a laser spot size of 25 μm was used to detect PNGase F–released N-glycans in a positive-ion mode. Using the Smartwalk feature set at a width of 30 μm and one scan per pixel, 200 laser shots per pixel were collected for analysis with an ion-accumulation time of 0.1 s. A 1.2059-s transient with a calculated resolving power of 160,000 at *m/z* 400 was used over a mass range of 500 to 5000 *m/z* and a time domain set to 512K words.

### MALDI–QTOF Analysis of N-Glycans

A timsTOF Flex trapped ion mobility–separated QTOF mass spectrometer (Bruker Daltonik) (*m/z* 500–4000) operating in a positive mode equipped with a 10-kHz Smartbeam 3D laser and a spot size of 20 μm was used to detect released N-glycans with high spatial resolution (20- to 40-μm raster). Three hundred laser shots per pixel were collected with an ion-transfer time of 125 μs, a prepulse storage time of 25 μs, a collision radio frequency of 4000 Vpp, a multipole radio frequency of 500 Vpp and a collision cell energy of 15 eV.

### MS Data Processing and Analysis

After MS data acquisition, spectra were imported to SCiLS Lab 2017a and 2020a imaging software for analysis of the mass range *m/z* 500 to 4000. SCiLS-generated N-glycan spectra were normalized to the total ion count (ICR Noise Reduction Threshold = 0.95), which were then matched within ±5 ppm against an in-house database of known N-glycans generated using GlycoWorkbench and GlycoMod for annotation (22). N-glycan structures annotated in this article are compositionally accurate as determined by accurate mass and through prior structural characterizations by both MALDI–TOF–MS/MS and reversed-phase liquid chromatography-coupled tandem mass spectrometry (23). Annotated collision-induced dissociation spectra for 23 common core N-glycan structures in this report are included in Supplemental Materials. Additional isomeric configurations were confirmed by complementary analyses with core fucose-specific Endo F3 and stabilization of sialic acids by amidation. A list of all N-glycans reported in this analysis along with mass error calculations and representative structures can be found in supplemental Tables S2 and S3.

### DAB Immunohistochemical Staining for CA19-9

FFPE (5 μm) whole-tissue block slides were dewaxed and rehydrated, and the antigen was retrieved as described above in preparation for IHC–DAB staining. Samples were sequentially incubated with 100 μl each of the peroxidase blocking reagent, serum blocking reagent, avidin blocking reagent, and biotin blocking reagent for 5 min, 15 min, 15 min, and 15 min respectively. CA19-9 primary antibody was diluted to 15 μg/ml in phosphate-buffered saline with Triton-X100 (50 μl Triton-X100, 50-ml PBS, pH 7.4) containing 0.025% serum blocking reagent, and tissue samples were incubated with the diluted antibody overnight at 4 °C. After incubation, samples were washed with PBS, incubated with 100-μl high-sensitivity streptavidin–HRP secondary antibody at RT for 1 h, and then developed with the DAB chromogen solution for 5 min with observation under a microscope. Stained samples were rinsed in water, dehydrated in the reverse gradation of EtOH solutions and xylenes, and then sealed and permanently mounted. Slides were subsequently imaged in a NanoZoomer 2.0-RS high-resolution slide scanner (Hamamatsu) at 40× magnification across the whole tissue. Scanned images were analyzed using Image Color summarizer, a freely available online k-means clustering–based algorithm, to generate an unbiased clustering of pixels by color to delineate between blank slide background, low-intensity CA19-9 staining, and high-intensity CA19-9 staining. Subsequently generated grouped pixel maps were then coregistered in flexImaging software with the original slide scans used for MALDI–IMS analysis to draw regions of CA19-9 staining for import into SCiLS lab software for analysis.

### CA19-9 and sTRA Immunofluorescence Staining

Paraffin was removed from 5-μm-thick FFPE sections using CitriSolv Hybrid, and then, the tissue was rehydrated through an EtOH gradient. After rehydration, antigen retrieval was achieved through incubating slides in citrate buffer at 100 °C for 20 min. Slides were then blocked in PBS with 0.05% Tween-20 (PBST0.05) and 3% BSA for 1 h at RT. Primary antibodies against CA19-9 and TRA-1-60 were labeled for immunofluorescent staining with sulfo-cyanine5 NHS ester and sulfo-cyanine3 NHS ester, respectively. Dialysis was performed after labeling to remove unreacted conjugate, and the primary antibodies were then diluted into the same solution of PBST0.05 with 3% BSA to a final concentration of 10 μg/ml. Slides were incubated overnight with this solution at 4 °C in a humidified chamber.

The following day, the antibody solution was decanted and the slides were washed twice in PBST0.05 and once in 1X PBS, each time for 3 min. The slides were dried via blotting and then incubated with 4′,6-diamidino-2-phenylindole at 10 μg/ml in 1X PBS for 15 min at RT. Two five-minute washes were performed in 1X PBS, and then, slides were coverslipped and scanned using a fluorescent microscope. All TMA slides were scanned using Vectra (Perkin Elmer), whereas the whole-tissue sections were scanned using an Axio Scan.Z1 (Zeiss). Each system collected data for each field of view at three different emission spectra. All image data were quantified using SignalFinder-IF.

After scanning, slides were stored in a humidified chamber. Coverslips were removed for the subsequent rounds of staining by submerging the slide in deionized water at 37 °C until the coverslip floated free (between 30 and 60 min). Fluorescence was quenched between rounds by incubating the slides with 6% hydrogen peroxide in 250-mM sodium bicarbonate (pH 9.5–10) twice for 20 min at RT. Subsequent rounds of staining were performed as described above using tetramethylrhodamine-conjugated anti–PHA-E and biotinylated GSL-II as the primary detections. Cy5-conjugated streptavidin was used as a secondary antibody to detect the biotinylated GSL-II for 1 h at RT. This was performed following washing after primary incubation.

To perform sialidase treatment, slides were incubated with a 1:200 dilution (from a 50,000 U/ml stock) of α2-3,6,8 neuraminidase in 1X GlycoBuffer (5-mM CaCl_2_, 50-mM, pH 5.5, sodium acetate) overnight at 37 °C. Slides were washed as described above and then antibody detections were performed. H&E staining was performed following a standard protocol.

### Experimental Design and Statistical Rationale

A total of 327 tissue cores across 4 TMAs representing 53 patients were analyzed in this study, with each core consisting of 171 individual spectra on average. Individual spectrum pixels in each core were averaged to generate a representative spectrum for the whole core, which was subsequently used for statistical analysis. Each patient in the study was represented at minimum by n = 2 adjacent normal tissue cores and n = 2 tumor tissue cores. SCiLS Lab was used to generate area under the peak values for TMAs after normalizing glycan intensity values to total ion current, which were subsequently exported to statistical software for analysis. Log_2_ fold change (FC) was calculated by comparing individual glycan intensities between normal and tumor tissues on a patient-matched basis. To determine statistically significant changes in glycan expression, area under the peak values were evaluated using a two-sample *t*-test assuming unequal variance. Thirteen whole-tissue blocks from which some TMA cores were derived were also used to draw qualitative conclusions about spatial localization trends across different PDAC regions. An automated thresholding program, SignalFinder-IF, was used to identify real signals on fluorescently labeled tissues. The algorithm uses multiround segmentation to separate nontissue and tissue backgrounds and signal levels of fluorescence (20, 24, 25). The immunofluorescence data were overlaid onto MALDI mass and H&E images using the custom-built Overlay program (5) and Canvas 14. Least absolute shrinkage and selection operator (LASSO)-regularized logistic regression was performed using the R “glmnet” package setting the mixing parameter alpha to 1 and setting lambda through cross validation to 0.0582. The mass-only model was similarly performed with a lambda set to 0.1017. Individual mass comparisons were performed using a paired t-test between tumor and adjacent normal cores from the same tissue and were plotted using the R “ggplot” package.

## Results

### N-Glycan Imaging of Normal Pancreas Tissue

**Fig. 1:**
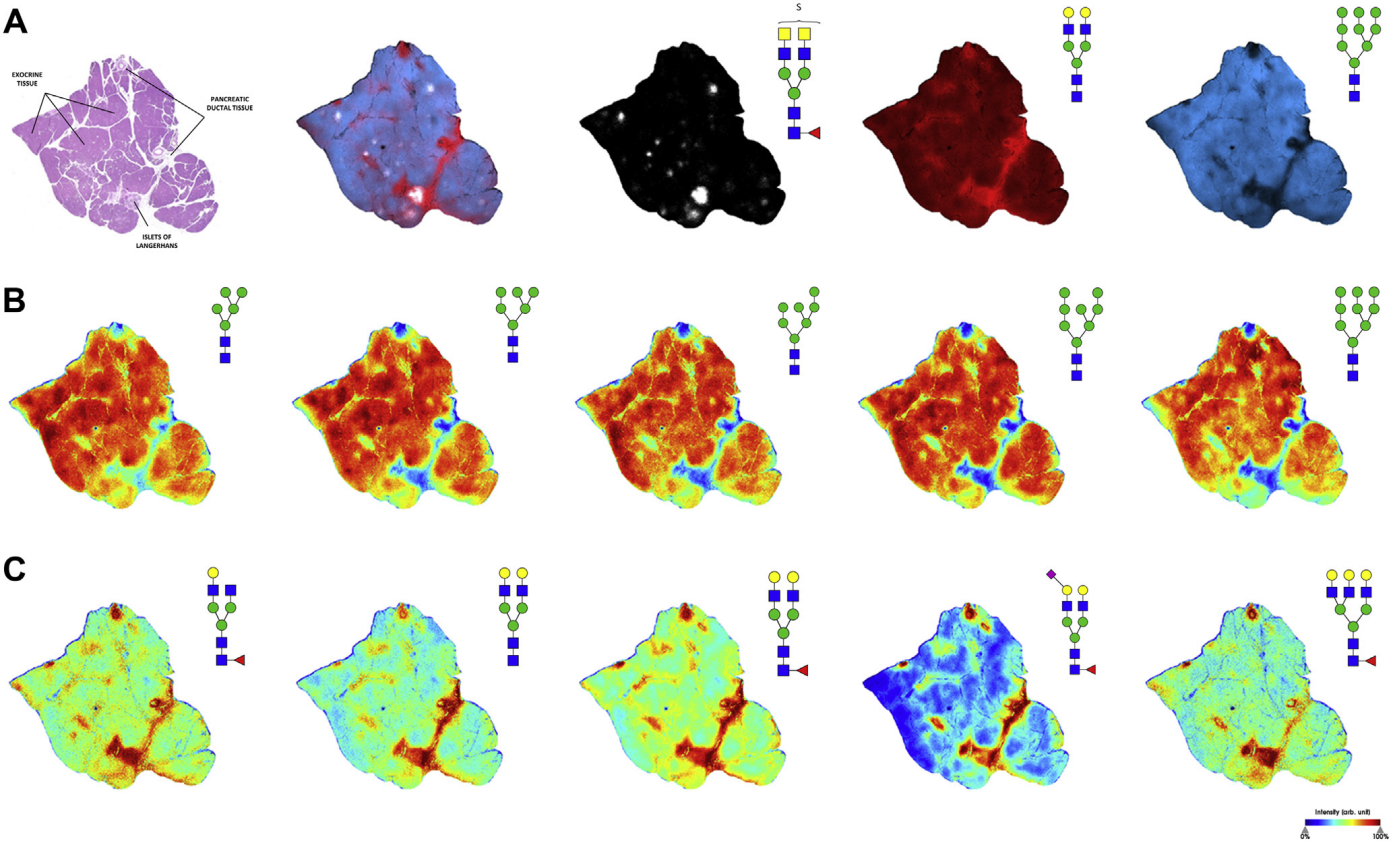
**N-glycan imaging of a normal human pancreas.***A*, the overlay image of three representative N-glycans corresponding to acinar exocrine tissue (*blue*, Hex9HexNAc2 *m/z* 1905.6338), ductal tissue (*red*, Hex5HexNAc4 *m/z* 1663.5814), and islets of Langerhans (*white*, Hex3dHex1HexNAc6SO_4_2 *m/z* 1993.6390) along with accompanying annotated H&E stain. *B*, high-mannose N-glycans predominantly localized to acinar exocrine tissue. *C*, N-glycans expressed predominantly in pancreatic ductal tissue tend to be biantennary and triantennary structures with core fucose residues lacking terminal fucosylation.

The goal of the study was to characterize N-glycan changes in normal pancreas and pancreatic cancer tissues; thus, an initial step was to define the distribution of N-glycans present in a normal pancreas, which comprises distinct endocrine, exocrine, and ductal cell types. A normal human pancreas tissue was antigen retrieved, sprayed with a molecular coating of PNGase F, and coated with CHCA matrix as described in Experimental Procedures section. Some selected tissues were also treated with Endo F3 to distinguish core fucosylated structures from outer arm fucosylated species. The enzymatically released N-glycans were detected first with an MALDI–FTICR mass spectrometer at a low spatial resolution (150-μm raster) and then again at a higher spatial resolution (30-μm raster, offset) using a MALDI–QTOF mass spectrometer according to a standardized protocol (26). Distinct differences in glycan patterning were observed between endocrine tissue, exocrine tissue, and ductal tissue subtypes (Fig. 1*A*). High-mannose N-glycan structures were primarily localized to pancreatic exocrine acinar tissue but were detected at lower levels in other tissue regions as well (Fig. 1*B*). N-glycans associated with interlobular and intralobular ductal tissue were primarily core-fucosylated biantennary and triantennary structures (Fig. 1*C*).

**Fig. 2:**
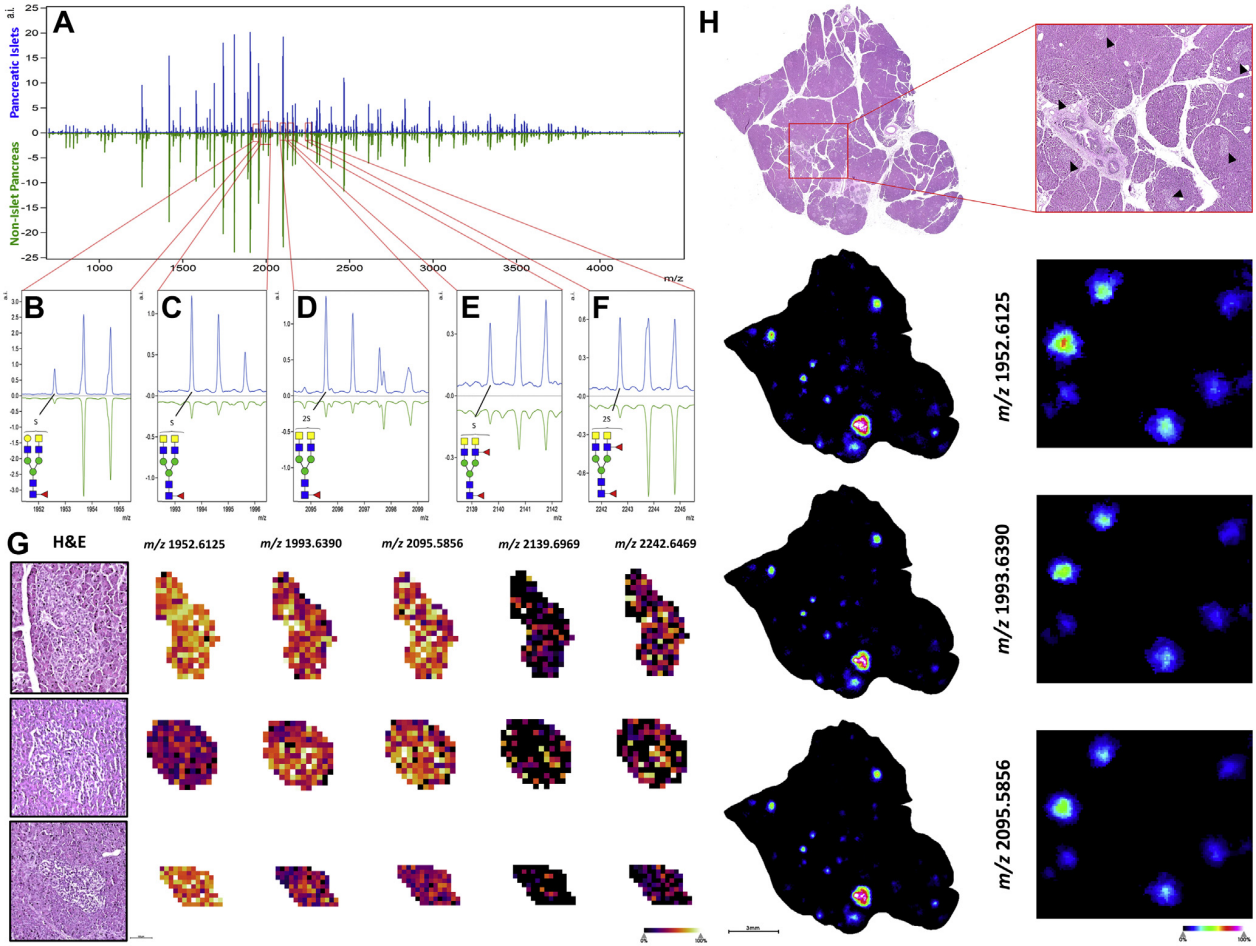
**Sulfated N-glycans with terminal GalNAc residues specific to pancreatic islets of Langerhans.***A*, comparative mass spectra of pancreatic islets of Langerhans (*blue*) and nonislet pancreatic tissue (*green*) from the same normal pancreas tissue section. Sulfated hybrid N-glycans, *B*, Hex4dHex1HexNAc5SO_4_, *m/z* 1952.6125; *C*, Hex3dHex1HexNAc6SO_4_, *m/z* 1993.6390; *D*, Hex3dHex1HexNAc6SO_4_2, *m/z* 2095.5856; *E*, Hex3dHex2HexNAc61SO_4_, *m/z* 2139.6969; *F*, Hex3dHex2HexNAc6SO_4_2, *m/z* 2242.6469 unique to endocrine islet tissue. *G*, targeted imaging of unique islet N-glycans along with representative H&E staining. *H*, nontargeted whole-tissue images of normal human pancreas showing specific localization of the 3 most abundant hybrid, sulfated N-glycan structures to islets of Langerhans tissue (*black carats*), which are clearly absent from other pancreatic tissue regions.

A more targeted N-glycan analysis of pancreatic islets (islets of Langerhans) was performed for normal human pancreas tissue. Before preparation for MALDI–IMS, the tissue was H&E-stained and scanned at 40× magnification before proceeding to antigen retrieval. These high-resolution histopathological images were coregistered in Fleximaging software to direct targeted glycan IMS of only pancreatic islets. These same tissues were then reanalyzed at the whole-tissue level to generate a profile of nonislet pancreatic tissue for comparison. Comparing the N-glycan profiles between islet and nonislet regions, detection of the most common N-glycans were generally consistent in both regions but with lower expression levels for most structures in the islets. (Fig. 2*A*). There were however a distinct set of sulfated N-glycans that were detected in islets that were either not present or expressed at much lower levels in nonislet regions. These 5 N-glycans were all sulfated and core fucosylated and had at least one terminal GalNAc residue, with some structures having outer arm fucose (Fig. 2, *B*–*F*). The mass-based structural predictions for these novel islet glycans were orthogonally confirmed via fragmentation by on-tissue collision-induced dissociation and Endo F3 analysis (data not shown). Sulfated N-glycan structures with terminal GalNAc have been previously reported to be detected in adult pig islet tissue (27). Targeted analysis images of these 5 structures for individual islets are shown in Figure 2*G*. In addition, whole-tissue scans of the normal pancreas tissue exhibited specific localization of these unique sulfated N-glycans to pancreatic islets, which were clearly absent from other nonislet pancreatic tissue regions (Fig. 2*H*).

A normal tissue section was digested with Endo F3 to assess the anomeric linkages of fucose residues in reported structures. Endo F3 cleaves N-glycans above the first GlcNAc residue on the chitobiose core in the presence of a fucose residue attached to that primary GlcNAc. Core fucosylated structures are thereby freed from their protein carriers with a distinct mass shift representing the loss of one GlcNAc residue and one fucose residue. By using a combination of Endo F3 and PNGase F in a 1:10 ratio, both core and terminally fucosylated N-glycan isomers can be released for analysis (supplemental Fig. S1*A*). Although some outer arm fucosylated isomers were detected (supplemental Fig. S1*B*), N-glycan structures with at least 1 fucose residue were predominantly core fucosylated (supplemental Fig. S1*C*), illustrated by the unique islet N-glycan *m/z* 1952.6125 for which no outer-arm fucosylated isomers were observed.

### N-Glycan Imaging of Advanced-Stage PDAC Tumor Tissues

**Fig. 3:**
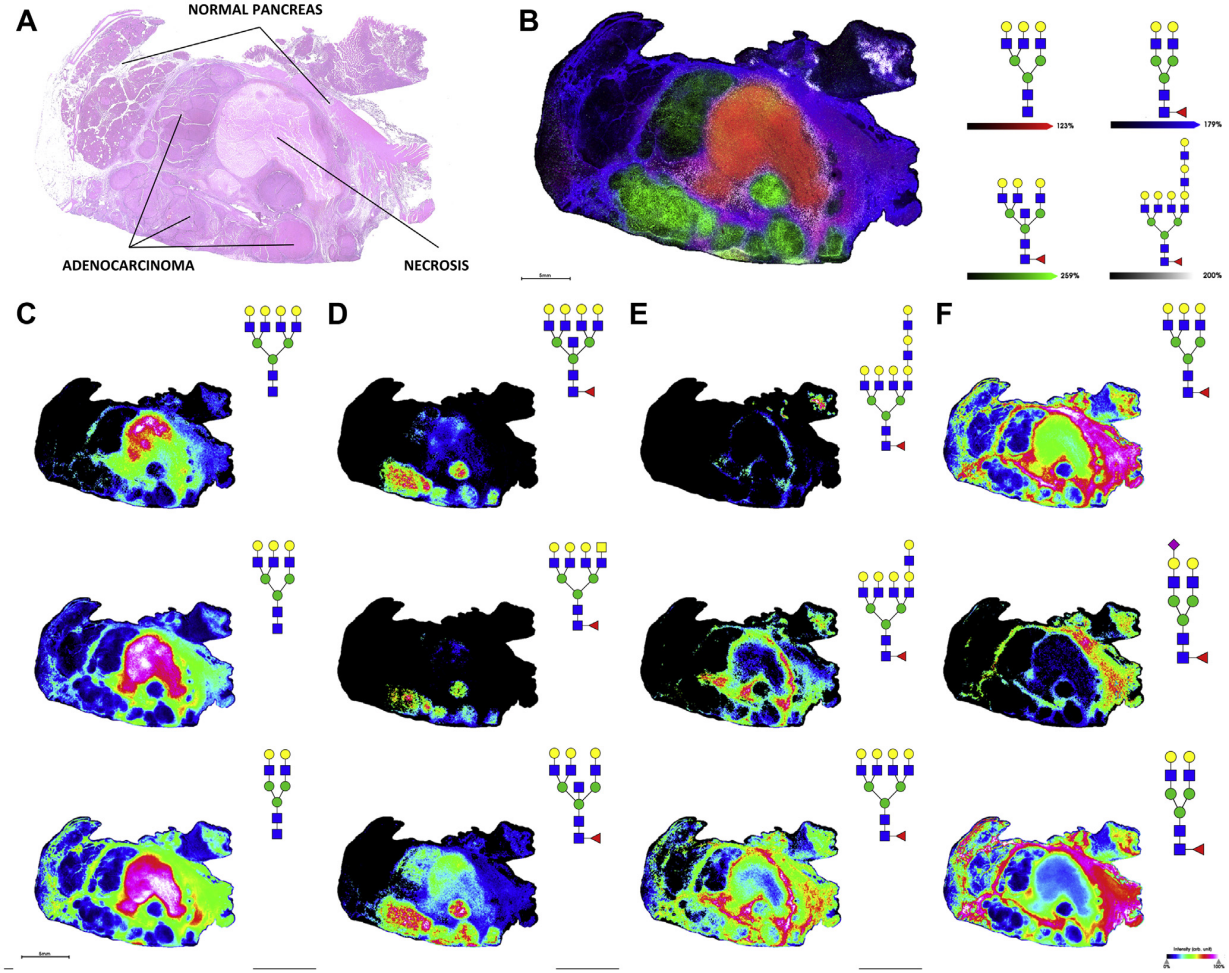
**N-glycan imaging of a representative stage 3 PDAC tumor tissue.***A*, H&E staining of T3NXMX PDAC tissue with annotations. *B*, overlay of 4 representative N-glycans corresponding to necrotic tissue (*red*, Hex6HexNAc5 *m/z* 2028.7136), adenocarcinoma (*green*, Hex6dHex1HexNAc6 *m/z* 2377.8509), tumor margin (*white*, Hex9dHex1HexNAc8 *m/z* 3270.1681) and adjacent non-tumor tissue (*blue*, Hex5dHex1HexNAc4 *m/z* 1809.6393). *C*, N-glycan structures localized to necrotic tissue (*top* to *bottom*) Hex7HexNAc6 *m/z* 2393.8458, Hex6HexNAc5 *m/z* 2028.7136, and Hex5HexNAc4 *m/z* 1663.5814. *D*, N-glycan structures localized to adenocarcinoma tumor (*top* to *bottom*) Hex7dHex1HexNAc7 *m/z* 2742.9831, Hex6dHex1HexNAc7 *m/z* 2580.9302, and Hex6dHex1HexNAc6 *m/z* 2377.8509. *E*, N-glycan structures localized to tumor margin (*top* to *bottom*) Hex9dHex1HexNAc8 *m/z* 3270.1681, Hex8dHex1HexNAc7 *m/z* 2905.0359, and Hex7dHex1HexNAc6 *m/z* 2539.9037. *F*, N-glycan structures localized to adjacent nontumor tissue (top to bottom) Hex6dHex1HexNAc5 *m/z* 2174.7715, Hex5dHex1HexNAc4NeuAc1 *m/z* 2100.7347, and Hex5dHex1HexNAc4 *m/z* 1809.6393. PDAC, pancreatic ductal adenocarcinoma.

To evaluate PDAC glycosylation by N-glycan MALDI–IMS, representative whole tissues were analyzed, and data were compared with a series of carbohydrate antigen–defined tissue microarray slides. These same samples were also evaluated for differences in isomeric sialic acid distributions. As an example, a PDAC tumor tissue staged at T3NXMX was analyzed for N-glycan composition differences by IMS. N-glycan spatial distributions could be linked with the histopathologically annotated primary tumor tissue, necrotic tissue, tumor margin tissue, and adjacent normal tissue regions as shown in the H&E-stained tissue (Fig. 3*A*) adjacent to four N-glycans detected in these regions (Fig. 3*B*). N-glycan structures primarily localized to necrotic tissue tended to be biantennary, triantennary, and tetra-antennary structures lacking any fucose or sialic acid residues (Fig. 3*C*), which has previously been reported for tumor necrosis regions of other cancer tissues (16). Core-fucosylated triantennary and tetra-antennary N-glycan species with bisecting GlcNAc modifications and terminal GalNAc residues were primarily localized to adenocarcinoma regions (Fig. 3*D*). The interface between tumor, necrosis, and adjacent normal tissue was defined by tetra-antennary core-fucosylated structures with terminal poly-LacNAc extensions of increasing length (Fig. 3*E*), whereas N-glycans localized to adjacent normal tissue were typically biantennary and triantennary structures with core fucose residues (Fig. 3*F*). High-mannose glycans were predominantly localized in normal tissue regions (data not shown).

**Fig. 4:**
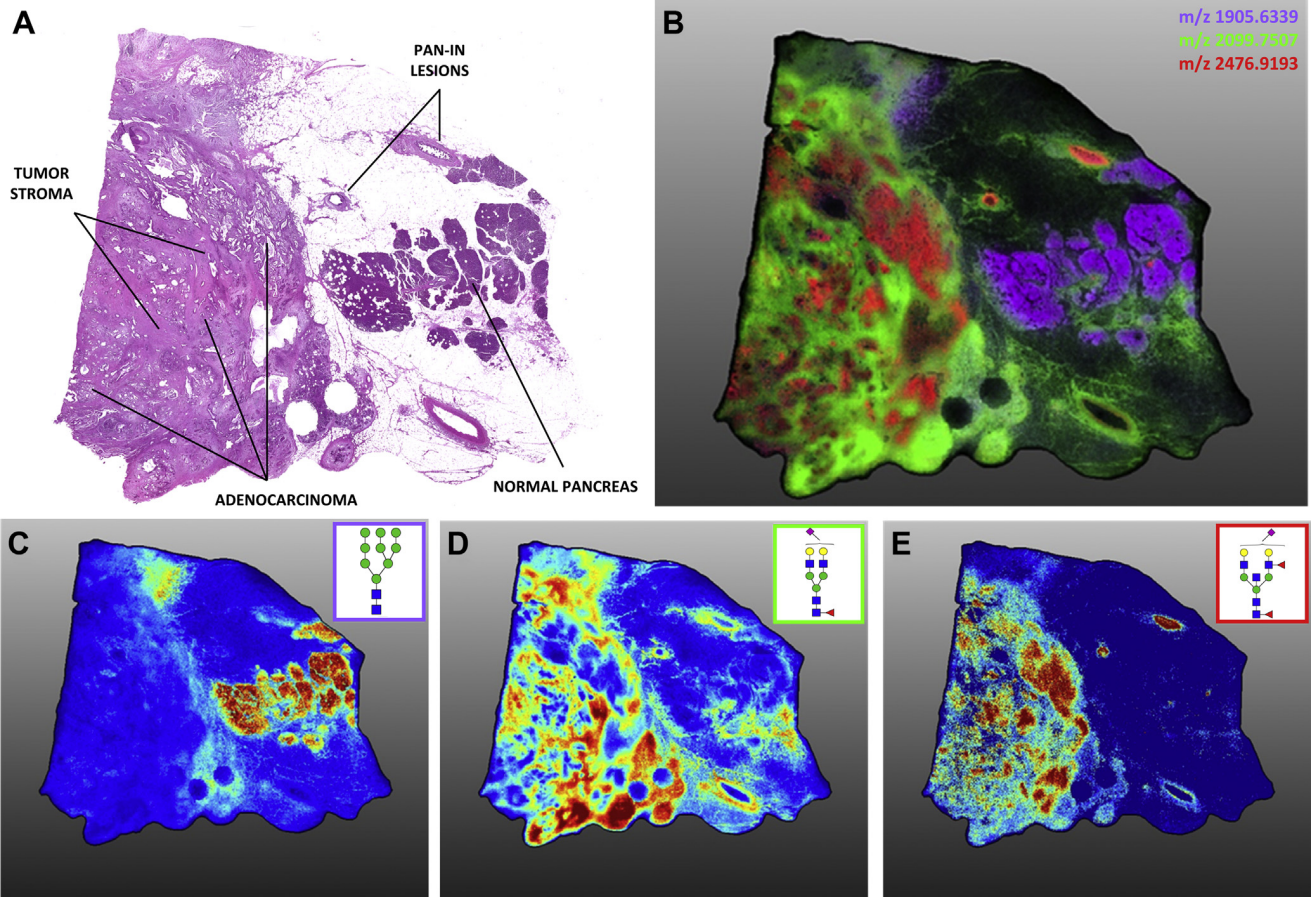
**Sialic acid linkage and expression differences between tissue types in an advanced-stage PDAC tumor tissue.** High-resolution (40 μm) MALDI-IMS of AA-stabilized sialylated N-glycans. *A*, annotated H&E staining of late-stage PDAC tumor. *B*, the overlay image of three N-glycan structures (depicted individually in panels *C–E*) localized to adjacent normal tissue (*purple*, Hex9HexNAc2 *m/z* 1905.6339), tumor stroma (*green*, Hex5dHex1HexNAc4NeuAc1(2,3) *m/z* 2099.7507), and adenocarcinoma (*red*, Hex5dHex2HexNAc5NeuAc1(2,6) *m/z* 2476.9193). *C*, high-mannose glycan structures tended to associate with normal adjacent tissue. N-glycan structures in normal tissue lacked significant sialic acid expression as compared with tumor tissue. *D*, α2,3-oriented sialic acid–decorated N-glycan structures localized to tumor stroma regions. *E*, adenocarcinoma-associated N-glycans were predominantly α2,6-sialylated although α2,3 vs. α2,6 sialylation of the same base structures drove different intratumor localization. These same α2,6-sialylated structures were also present within Pan-IN lesions. IMS, imaging mass spectrometry; Pan-IN, pancreatic intraepithelial neoplasia; PDAC, pancreatic ductal adenocarcinoma.

An on-tissue sialic acid derivatization reaction was performed on a different PDAC tumor tissue to assess differential patterns of sialylation. The amidation–amidation chemistry derivatizes the sialic acid 1ʹ carboxyl group differentially for α2,3- and α2,6-linked residues, introducing a mass shift that allows for the delineation of these isomers (21). This pancreatic cancer tissue, shown in Figure 4, contained late-stage adenocarcinoma (T3N1MX) tissue surrounded by a network of tumor stroma as well as pancreatic intraepithelial neoplasia lesions and adjacent normal tissue (Fig. 4*A*). An overlay image of three representative N-glycan structures corresponding to these regions is shown in Figure 4*B*. Adjacent normal tissue in this PDAC sample contained the same abundance of high-mannose structures seen in other tissues analyzed in this report (Fig. 4*C*) and few sialylated N-glycans were localized to this region. In contrast, most structures detected in regions of primary tumor or tumor stroma tissue contained one or more sialic acid residues. The N-glycans from tumor stroma regions were typically α2,3 sialylated (Fig. 4*D*). This differed from areas of primary adenocarcinoma, which were a mixture of α2,3 and α2,6 sialylation slightly favoring α2,6 sialylated species (Fig. 4*E*). Interestingly, differential α2,3 versus α2,6 sialylations of the same base N-glycan structures indicated distinct spatial localizations of these structures within the same tissue and even within the same tissue region, suggesting possible compartmentalization of specific sialyltransferases to distinct tissue regions and subtypes in pancreatic cancer. A table of N-glycan compositions and structures detected in this tissue and other analyses throughout this report is provided in supplemental Table S3.

### N-Glycan Imaging of Carbohydrate Antigen–Classified PDAC Tissue Microarrays

**Fig. 5:**
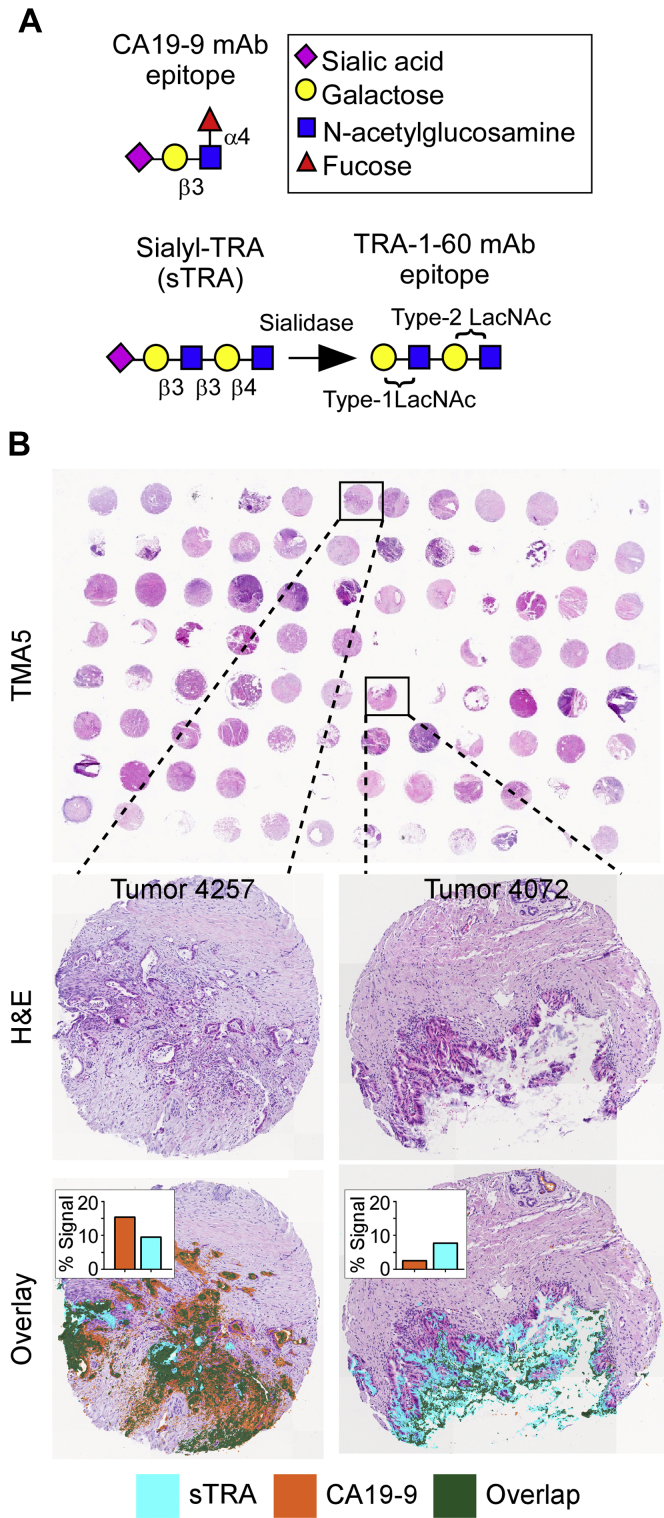
**Quantifying biomarker epitopes in tissue samples using immunofluorescence.***A*, treatment of the sTRA epitope with sialidase exposes the TRA-1-60 epitope, allowing for detection with anti–TRA-1-60 mAb. *B*, tissue microarrays (TMAs) were used to obtain marker data on primary PDAC and related tissues from multiple patients. TMAs were stained using multi-round immunofluorescence with subsequent H&E staining and imaging. Each core was quantified to determine the percentage of tissue pixels above background. PDAC, pancreatic ductal adenocarcinoma.

A more comprehensive assessment of the PDAC N-glycome was performed using four tissue microarrays representing 53 individual patients including matched adjacent normal tissue, tumor tissue, and metastatic tissues as well as some patients with samples of pretumor lesions, normal lymph node tissue, and cancerous lymph node tissue. More detailed information about patient samples can be found in supplemental Table S1. N-glycans were released from these tissue arrays as described above and N-glycan intensities across the tissue cores were analyzed via MALDI–FTICR–MS. In conjunction to analysis by IMS, serial TMA sections were analyzed via multiround immunofluorescence for the CA19-9 and sTRA biomarkers (Fig. 5*A*) followed by H&E staining as previously described (20). This system allows for high-resolution and high-sensitivity quantification of multiple markers on one tissue section, while also preserving the ability to acquire bright-field imaging of the cellular histology using H&E staining. An in-house image processing program called SignalFinder was used to automatically locate and quantify the signals from each marker. The maps of identified signal can be overlaid onto the bright-field images to gain information about the types of cells that produce each marker as well as overlaps in marker expression (Fig. 5*B*). These analyses drove classification of tumor cores as “both” (significant CA19-9 expression and significant sTRA expression), “CA19-9 only” (significant CA19-9 expression with low sTRA expression), “sTRA only” (low CA19-9 expression with significant sTRA expression), or “neither” (low in CA19-9 expression and low in sTRA expression). These designations subsequently informed statistical analyses of differential N-glycan patterns between varying levels of TMA biomarker expression.

**Fig. 6:**
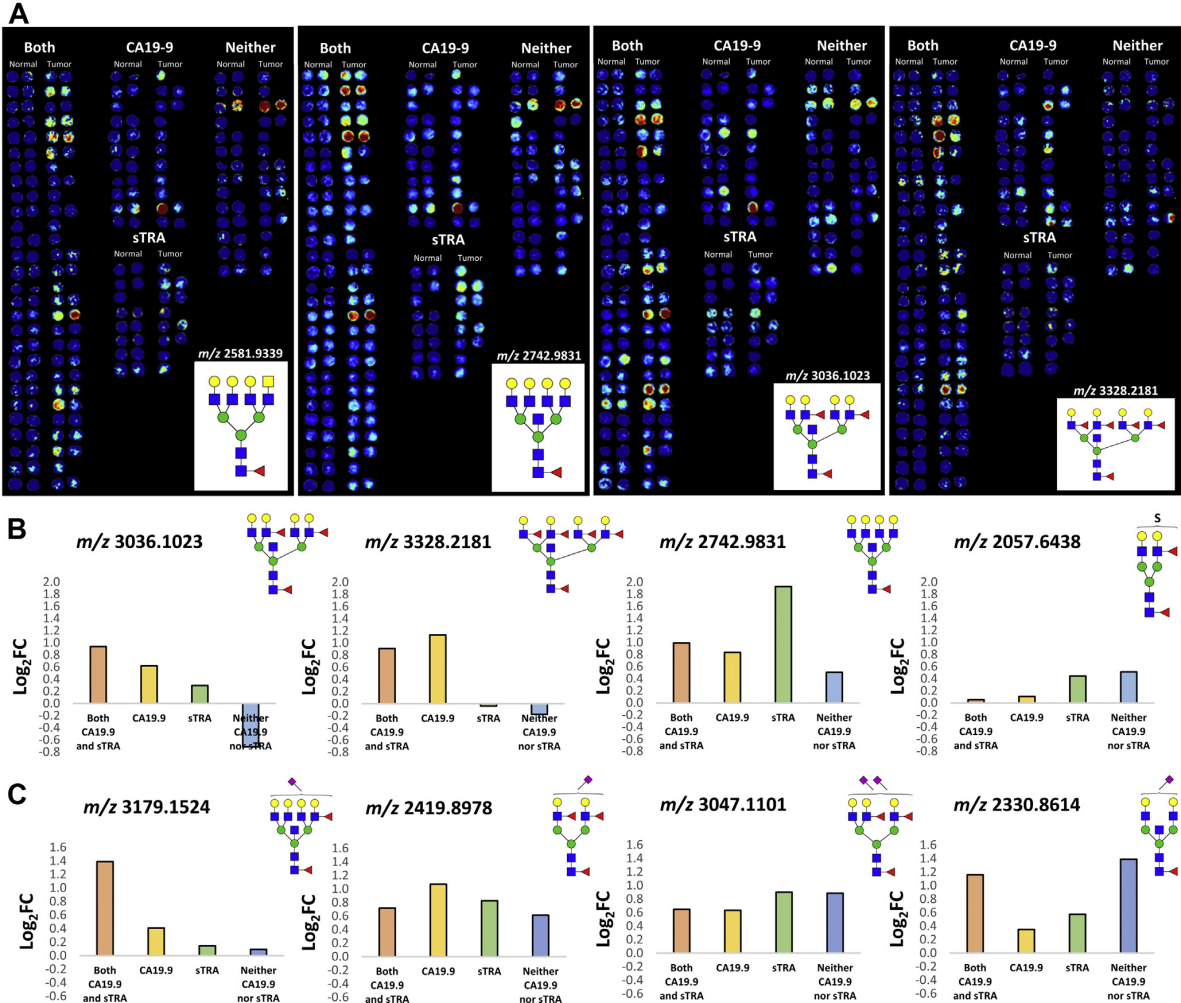
**TMA cores grouped by CA19-9 and sTRA expression.***A*, individual patient core pairs analyzed by SCiLS Lab were extracted from each of the 4 TMAs and grouped in one of four designations based on biomarker expression. Four representative sorted TMA heat maps for N-glycans *m/z* 2581.9339, *m/z* 2742.9831, *m/z* 3036.1023, and *m/z* 3328.2181 are shown. Average Log_2_ fold change between patient-matched tumor and normal cores across the four groupings for (*B*) nonsialylated N-glycans and for (*C*) amidation–amidation–stabilized sialic acid containing N-glycans. Positive values represent enrichment in tumor tissues. CA19-9, carbohydrate antigen 19-9; sTRA, sialylated tumor-related antigen; TMAs, tissue microarrays.

Tumor cores and their matching nontumor tissue cores were analyzed in SCiLS Lab to elucidate differential N-glycan expression between the groups. Heat map images of specific N-glycan structure distributions, normalized to the total ion count, were generated, and then, cores were grouped according to their biomarker-classified designations for visualization (Fig. 6*A*). N-glycans significantly associated with CA19-9 expression tended to be core-fucosylated biantennary, triantennary, and tetra-antennary structures with bisecting GlcNAc residues and multiple outer arm fucosylations. These structures were detected less frequently in tumor cores with high sTRA expression, which instead tended to be triantennary and tetra-antennary structures lacking outer arm fucosylation. Average Log_2_ FC between patient-matched tumor and normal cores is shown in Figure 6*B*. These data and average intensity data across the four biomarker-classified groupings are provided in supplemental Table S4 and were used in further evaluations in the next section. The same TMAs were also treated by the sialic acid amidation–amidation stabilization reactions before the standard imaging workflow and analyzed by MALDI–IMS. In general, levels of sialylation were significantly elevated in all tumor tissues relative to normal controls, summarized in supplemental Table S5 for Log2 FCs and intensities of each glycan. Like that shown for the whole tissue, the abundance of α2,3 sialylated glycans was higher relative to α2,6 sialylated glycans, but there was more specificity of the α2,6 glycans to distinguish biomarker antigen subtypes (Fig. 6*C*, supplemental Fig. S2 and supplemental Table S5).

### N-Glycan Profiles Indicate Potential Complementarity to Current Biomarkers

**Fig. 7:**
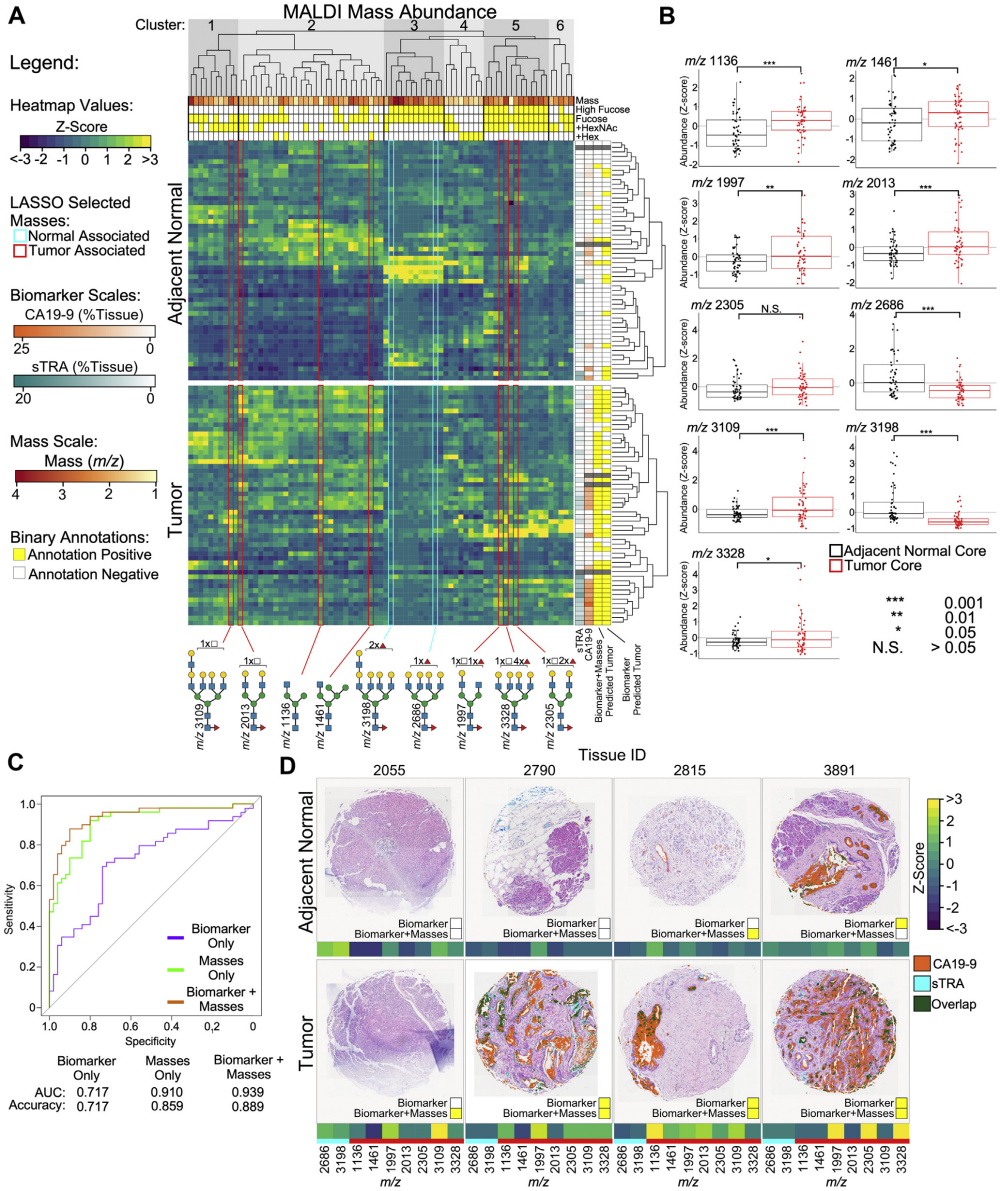
**Mass abundance indicates glycan structures with potential complementary value to current biomarkers.***A*, hierarchical clustering and LASSO-regularized logistic regression identified families of masses with potential complementary value to known PDAC biomarkers. Masses selected by LASSO regularization are highlighted and color-coded for tumor-core associated or normal-core associated. Cores predicted to be tumors are indicated by a *yellow box* on the right. *B*, individually, the masses selected by LASSO regularization used in the logistic regression model have significantly different abundances between adjacent-normal and tumor cores. *C*, receiver operating characteristic curves of the use of the models to distinguish the tumor cores from the adjacent-normal cores indicate an improved accuracy using the combination of masses and biomarkers. *D*, example cores illustrate the complementary value of the masses and the biomarkers. The *yellow box* indicates prediction as a tumor core. The addition of the masses correctly identified a tumor core missed by the biomarkers (2055) and correctly identified a normal core falsely called tumor by the biomarkers (3891). In other instances, the combination produced no change (2790) and falsely predicted a normal core to be a tumor core (2815). LASSO, least absolute shrinkage and selection operator; PDAC, pancreatic ductal adenocarcinoma.

The extensive glycan data generated in the TMAs that are summarized in Figure 6 and supplemental Table S4 were further analyzed in combination with carbohydrate antigen data available for the same tissue cores. There exists a number of N-glycan differences between adjacent normal and tumor cores that suggest a potential for expansion of the biomarker repertoire. Hierarchical clustering of mass data resulted in clusters with distinct glycan properties and patterns for adjacent normal and cancer cores (Fig. 7*A*). For example, the masses of clusters 1 and 5, which are more abundant in the tumor cores relative to adjacent normal, generally have an unbalanced N-acetylhexosamine (HexNAc), meaning a terminal GlcNAc or GalNAc. In contrast, the normal-associated glycans of cluster 3 are mainly high-mass, balanced glycans with more than one fucose, and the tumor-associated glycans of cluster 2 are mainly nonextended, hybrid N-glycans. This result suggests that specific features may be present on glycans of different masses, which are expressed in the same tissues.

Using LASSO-regularized logistic regression, masses that offered predictive value complementary to the CA19-9 and sTRA biomarkers were identified, 9 masses in total with 7 masses being associated with tumor cores. The LASSO regression selects a subset of predictors with minimal collinearity and identifies the most informative masses from the tumor and normal associated clusters. Each of the selected masses had an abundance that was significantly different between the tumor and the adjacent-normal cores. When used in a predictive model in combination with the glycan biomarkers, overall predictive value was increased relative to using the biomarkers or the masses alone (Fig. 7*C*). The area under the curve in receiver operating characteristic analysis was 0.939 using the combination, greatly improved over the value of 0.717 using the biomarkers alone and moderately improved over the value of 0.910 using the masses alone.

We further examined cores for which the immunostaining and glycan imaging were complementary to each other. Figure 7*D* shows cores from 4 tumors and their corresponding, adjacent-normal areas. In the adjacent normal cores, tissues 2055 and 2790 are examples that were correctly predicted to be normal by both the biomarker and the combination, whereas 2815 and 3891 were incorrectly predicted to be tumor cores by the combination and the biomarker, respectively. In each of the tumor sections, either the biomarker or the glycans provided a positive signal. Tissue 2790 represents a strongly predicted tissue by biomarker alone, whereas tissue 2815 was predicted more strongly through the high expression of the lower mass, hybrid and immature N-glycan structures.

### Whole-Tissue N-Glycan Imaging of CA19-9–Stained PDAC Tumor Tissues

To obtain more information about interesting glycans identified in the TMA, whole-tissue sections corresponding to the cores highlighted in Figure 7*D* were analyzed by immunofluorescence. The TMA analysis showed that glycans with a terminal GlcNAc or GalNAc could be higher in certain tumors. Lectin immunofluorescence staining provides an additional layer of detail to mass abundances, as it allows for distinguishing between mass isomers and binding a range of masses that share glycan motifs. We chose the lectin PHA-E to detect bisecting GlcNAc and the GSL-II to detect terminal GlcNAc.

We first coregistered the IMS data from the relevant glycans with stains for CA19-9 expression obtained via HRP-DAB IHC on serial sections (supplemental Fig. S3, *A*–*B*). Biantennary, triantennary, and tetra-antennary N-glycans with both core and terminal fucosylation were specifically colocalized with areas of CA19-9 staining as determined by coregistration analysis in SCiLS Lab (supplemental Fig. S3*C*). Segmentation analyses of 77 detected N-glycan structures revealed distinct glycoprofiles between those areas of high CA19-9 expression and those with little to no expression (supplemental Fig. S3, *D*–*E*).

**Fig. 8:**
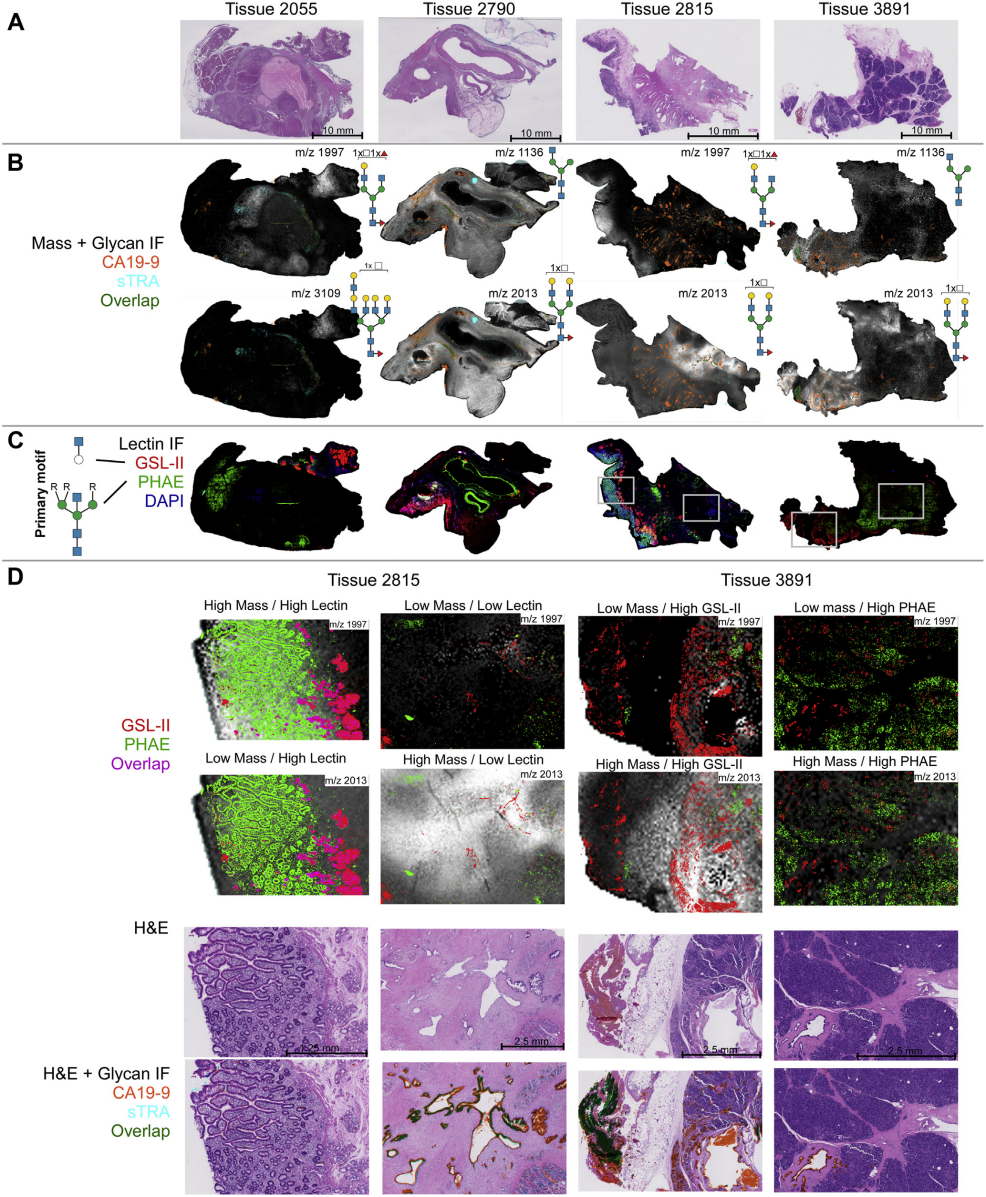
**Immunofluorescence stains reveal complex relationships between biomarkers and help distinguish terminal from bisecting GlcNAc**. Whole-tissue sections of patients that contain both cancer and adjacent normal regions were investigated. *A*, H&E-stained tissue sections. *B*, immunofluorescence (IF) signal above threshold for biomarkers CA19-9 and sTRA overlaid on select complementary mass abundance. *C*, lectin-stained IF images above threshold. The primary motif for PHA-E (*green*) is a bisecting GlcNAc with two or three antennae, whereas the primary motif for GSL-II (*red*) is a terminal GlcNAc presented on an unbranched monosaccharide. *D*, selected regions of interest in tissues 2815 and 3891 indicate a mixed relationship between lectin binding, mass abundance, and biomarker presence. Regions low and high in biomarker (CA19-9 and sTRA) expression are given for each. CA19-9, carbohydrate antigen 19-9; PHA-E, phaseolus vulgaris erythroagglutinin; sTRA, sialylated tumor-related antigen.

H&E stains on the whole-tissue sections revealed diverse tissue morphologies (Fig. 8*A*). The immunofluorescence stains and MALDI–MS analysis showed similar patterns as the selected TMA cores in Figure 7*D*, with tissue 2055 featuring low levels of biomarker expression, whereas the remaining tissues have regions of high and low expression. By overlaying the biomarker data on the mass abundances (Fig. 8*B*), one can observe a complex relationship between the glycans identified by MS and the biomarkers. Tissues 2815 and 3891 have significant diversity, featuring regions with all combinations of high and low mass abundance and biomarker expression.

The lectins PHA-E and GSL-II had very distinct, largely nonoverlapping staining (Fig. 8*C*), indicating that these two epitopes are characteristic of different types of cells. Regions of interest highlighted in Figure 8*D* show the diversity in mass abundance, lectin staining, and biomarker expression. Notably, tissue 2815 shows regions high in m/z 1996.7238 and lectin staining as well as regions high in m/z 2012.7187 and biomarker expression with little lectin staining. In contrast, tissue 3891 shows regions with high m/z 2012.7187, biomarker expression and GSL-II staining, as well as regions which are low in the highlighted masses but show strong PHA-E staining. Despite the combined binding specificity for PHA-E and GSL-II for many +HexNAc containing structures, regions exist in all 4 highlighted tissues that have high mass abundance but low lectin binding. Although this may be due to limited sensitivity of the lectins, it could also indicate that these regions feature mass isomers that do not have the primary binding motifs of these lectins. Such glycans could include tetra-antennary bisecting GlcNAc glycans, glycans with terminal GlcNAc presented on a branched chain, blood group A–presenting glycans, and alpha-GalNAc–terminated glycans. In sum, the combination of lectin staining with MSI data gives more information on the glycans containing the lectin epitopes and it serves to shed light on the isomeric variants that are present for particular masses identified.

## Discussion

This study describes an initial effort to determine the tissue N-glycome of the human pancreas and pancreatic cancer using formalin-fixed clinical specimens. Unique to the pancreas is the finding that high-mannose N-glycan structures are expressed abundantly in nondiseased organ tissue and normal tissue adjacent to cancerous regions (Figs. 1 and 4) but are not detected with high abundance in the tumor regions. This is in contrast to other cancer tissues that all exhibit tumor-localized abundant detection of high-mannose structures, including prostate, ovarian, bile duct, breast, and colorectal cancers (12, 28, 29, 30). It is suspected that the abundance of high-mannose structures in these cancers is due to incomplete N-glycan processing (31). This does not appear to be the case for pancreas but rather a feature of healthy tissue whose functions have yet to be determined.

The analysis of normal pancreas detected multiple sulfated, terminally GalNAcylated biantennary N-glycan structures in islets (Fig. 2). As mentioned previously, sulfated N-glycans of the same composition were reported in adult pig islet transplant studies. Sulfation is already known to play a role in pancreatic islets, where in that glycolipid sulfatides are involved in normal insulin secretion (32) and sulfation of heparin sulfate has been implicated in islet amyloid polypeptide aggregation in type 2 diabetes mellitus (33). Sulfated N-glycans on immunoglobulin G have also been extensively characterized, where these acidic modifications can lead to fine-tuned immune responses (34). Sulfated N-glycan structures, especially those with sulfo-sialyl-Lewis X motifs, are known to enhance lymphocyte homing and are implicated in interactions with other immune cell populations (35). Terminal sulfo-GalNAc residues found on N-glycans specific to pituitary hormones lutropin and thyrotropin are known to govern immune interactions with macrophages via the C-type mannose receptor 1 (36). Because the pancreas is a highly dynamic site of immune regulation, it is possible that these unique sulfated, terminally galactosylated islet structures play a role in normal immune recognition and homeostasis.

Key N-glycan structural themes associated with PDAC compared with nontumor tissues were increased sialylation, poly-LacNAc extensions, branching and extensive fucosylation of high-mass N-glycans. Also observed were structures with bisecting GlcNAc residues and terminal GalNAc (di-LacNAc) modifications. Low levels of sialylation were seen in normal tissue, whereas α2,3 sialylated N-glycans were expressed abundantly in TMA tumor cores. α2,6-Sialylated structures were detected at lower levels than their α2,3 counterparts but were typically more specific to primary adenocarcinoma clusters. This is in accordance with studies of pancreatic cell lines, which showed increased but varied α2,3/α2,6 content across different models as compared with normal pancreatic cells, with α2,3 sialylation implicated in a more metastatic phenotype, as well as with studies of pancreatic cancer in mouse models that observe β-galactoside α2,6-sialyltransferase 1 overexpression and subsequent increases in α2,6-linked sialic acid in aggressive metastasis (23, 37, 38). It should also be noted that CA19-9 and sTRA epitopes circulating on their glycoprotein carriers are α2,3-sialylated. A lack of fucose and sialic acid residues characterized N-glycans in necrotic PDAC tissue in this study, which is consistent with findings from previous glycan MALDI imaging analyses of breast cancer tissues and other tumor tissue types that detected similar necrosis-associated glycans (16). Increases in N-glycan branching is attributable to increased alpha-1,6-mannosylglycoprotein 6-beta-N-acetylglucosaminyltransferase A expression that extends triantennary structures into tetra-antennary N-glycans. Alpha-1,6-mannosylglycoprotein 6-beta-N-acetylglucosaminyltransferase A has been shown to be upregulated in PDAC in response to Kirsten RAS oncogene –mediated oncogenesis (39). Similarly, prior studies have demonstrated increased amounts of α1,3-linked fucose present in certain subsets of pancreatic cancer cell lines (40). Another study reported expression of α-(1,3/1,4)-fucosyltransferase, which is one of the FUT enzymes responsible for adding fucose residues to N-glycan antennae, is upregulated in pancreatic cancers and is involved in the development of a more metastatic phenotype (41).

Another goal of the study was to better link N-glycan structures associated with biomarker expression by integrating mass spectrometry and IHC data. Consistently, N-glycan structures that colocalized with CA19-9 immunostaining in tissues (see supplemental Fig. S3) did not contain a sLeA motif (Hex[β1,3](Fuc[α1,4])HexNAc) as assessed by IMS. CA19-9 epitopes have been detected specifically on mucin glycoproteins mucin 5AC and mucin 16 in patient populations with PDAC, detection of which bolsters the performance of cancer identification when combined with clinically used CA19-9 panels (42). These mucin glycoproteins are predominantly O-glycosylated, and it may be more likely that the CA19-9 epitope is present in these O-glycans. Studies of commonly used pancreatic cancer cell lines have demonstrated variable levels of CA19-9 and sTRA expression between cell lines (5). Other analyses of normal pancreas and PDAC cell lines have shown a complex relationship between the expression of Lewis antigens B, Y, sLeA, and sialyl-Lewis X which in some lines correlated with increased metastatic capacity, whereas in others showed little to no correlation (23). These heterogeneous results demonstrate the challenges of using one or few PDAC cell lines to accurately study the common pancreatic cancer biomarkers. Linking specific N-glycans to CA19-9 and sTRA biomarker expression levels is currently inconclusive because of the enormous complexity of the N-glycan structures detected in the CA19-9 and CA19-9/sTRA tissues. The N-glycans detected represent every major feature of complex N-glycans including bisecting GlcNAc, LacNAc extensions, di-LacNAc, and multifucosylation and sialylation modifications. Although an extensive number of N-glycans were detected in the tumor regions, there are an unknown number of higher mass glycans of m/z > 5000 that cannot be effectively detected by MALDI–FTICR directly from tissue. Stability of sialic acids in the MALDI process is an additional factor, which is mitigated by softer ionization environments in the MALDI–FTICR (9, 43) and can be further addressed by amidation stabilization. This aids in detecting high mass sialylated glycans, and additional methods are being explored to better link antigen motifs with specific N-glycans in PDAC tissues.

The cumulative data generated from the IMS glycan tissue analyses also allowed integration of the detection of individual N-glycans with results from existing antibody-based linear regression models for the classification of PDAC tumor vs. normal tissue samples (5). Improvements in receiver operating characteristic accuracy and area under the curve were observed when both IMS glycan masses and biomarker data were combined to build linear regression models as compared with models based on those analyses individually. These results demonstrate a complex relationship between mass spectrometry and antibody–biomarker characterization of these tissues which can be exploited to bolster classification algorithms. Complementary IMS and biomarker staining data enhance the confidence of tumor vs. nontumor tissue calls in our modeling. In addition, cases where biomarker data are lacking or insufficient can be rescued and correctly classified by the incorporation of IMS data into these models. The ability of these separate techniques to inform one another and compensate for the limitations of either model is demonstrated herein and suggests future utility for combining cross-disciplinary analyses such as these into existing PDAC classification assays. The N-glycan distribution maps can also be used to select other lectins not previously evaluated for staining for more targeted screening of glycan motifs. The PNGase F-based IMS methods is specific for N-glycan species, and combined use with multi-lectin stains can address O-linked antigens, which are well known in PDAC. Lectin staining provides the opportunity to parse out further structural characterizations in our mass spectrometry data via the ability of specific lectins to recognize specific carbohydrate motifs. These data layered into analysis by mass spectrometry offer the possibility of identifying specific N-glycan isomers which are typically indistinguishable by most IMS systems.

In summary, an initial N-glycome analysis of biomarker-defined PDAC tissues using N-glycan MALDI–IMS approaches illustrates the potential to inform more targeted probing of specific N-glycans and N-glycan structural classes that correlate with current and prospective carbohydrate biomarkers of PDAC. The glycan maps generated will be used to target regions of interest to identify potential glycoprotein carriers. Experiments to elucidate the glycoprotein carriers of the N-glycans highlighted in this study, as well as analyses of N-glycosylation in blood plasma samples from the same PDAC cohort, are already underway. Combining IMS with antibody immunostaining improves detection and classification of PDAC and informs on expanded directions for each method. Advancements in IMS techniques and instrumentation and the use of new enzymes and chemistries to reveal a broader repertoire of structures, isomers, modifications, and molecules will expand these approaches beyond the N-glycome. The continued search for new lectins that recognize carbohydrate structures associated with PDAC, as well as improvements to multiround immunofluorescence technologies and advancements in statistical image analysis software, potentiates the discovery of further, novel subclasses of pancreatic cancer. Observations about PDAC biomarkers and N-glycan structures from this and ongoing studies may provide leads to impactful new diagnostic and surveillance strategies critically needed in the clinic, as well as continued insights into the evolving role of the glycome in this disease.

## Data availability

Imaging mass spectrometry N-glycan accurate mass, intensity, and log_2_ fold change data for the tissue microarrays analyzed in this article are summarized in supplemental Tables S4 and S5. Raw imaging mass spectrometry data used in this report to be made available upon request, contact Dr Richard Drake, Medical University of South Carolina, draker@musc.edu.

## Conflict of interest

Authors declare no competing interests.
