# Supplementary material for: Imaging Mass Spectrometry and Lectin Analysis of N-Linked Glycans in Carbohydrate Antigen–Defined Pancreatic Cancer Tissues
Source: Mol Cell Proteomics. 2020 Dec 8;20:100012. doi: 10.1074/mcp.RA120.002256 (PMC8724603; doi:10.1074/mcp.RA120.002256)
Supplement: Supplementary file 1 — Supplemental Material [file mmc1.pdf]

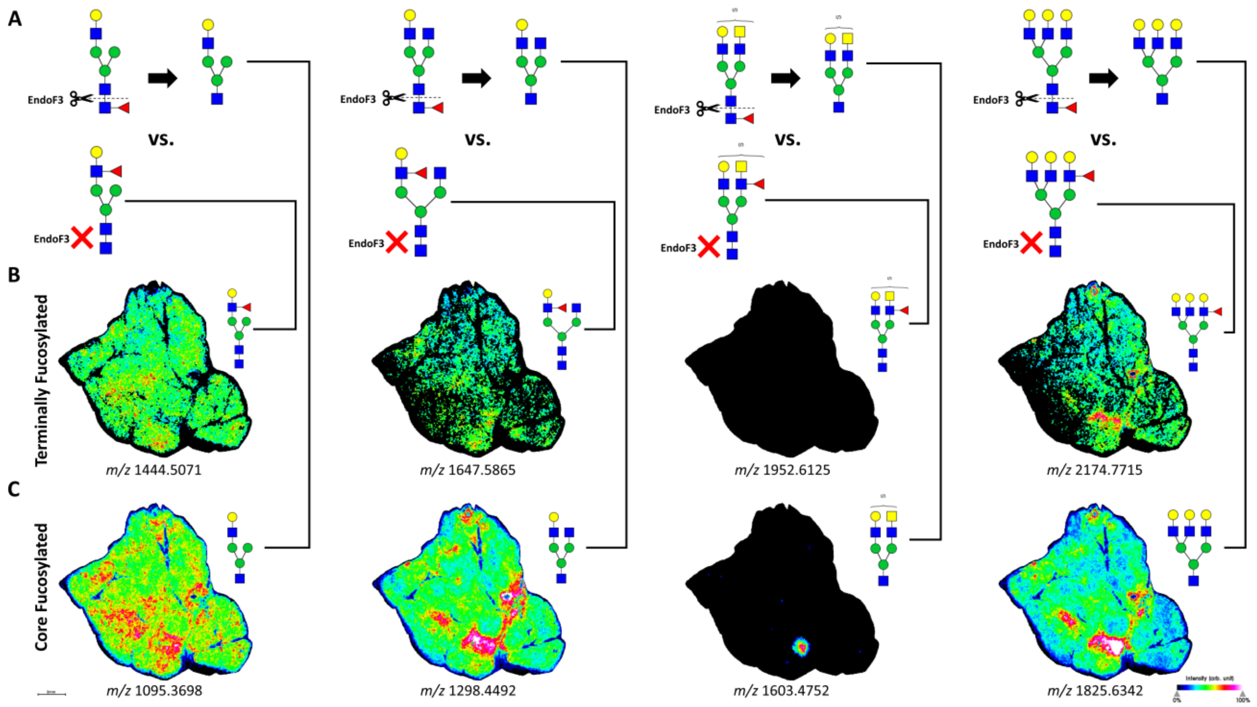

Figure S1. Endoglycosidase F3 reveals core fucosylated N-glycans. A) Endo F3 cleaves N-glycans between GlcNAc residues on the chitobiose core only in the presence of a core fucose residue and is unable to cleave terminally fucosylated isomers. B) Terminally fucosylated isomers were detected minimally (Hex4dHex1HexNAc3 *m/z* 1444.5071, Hex4dHex1HexNAc4 *m/z* 1647.5865, Hex6dHex1HexNAc5 *m/z* 2174.7715) or not at all (Hex4dHex1HexNAc5$SO_4$1 *m/z* 1952.6125). C) Endo F3 cleavage products from core fucosylated isomers with a distinct -349.1373 mass shift were observed in much greater abundance for the four representative structures shown (Hex4HexNAc2 *m/z* 1095.3698, Hex4HexNAc3 *m/z* 1298.4492, Hex4HexNAc4$SO_4$1 *m/z* 1603.4752, Hex6HexNAc4 *m/z* 1825.6342) as well as for most structures with at least one fucose residue in this report.

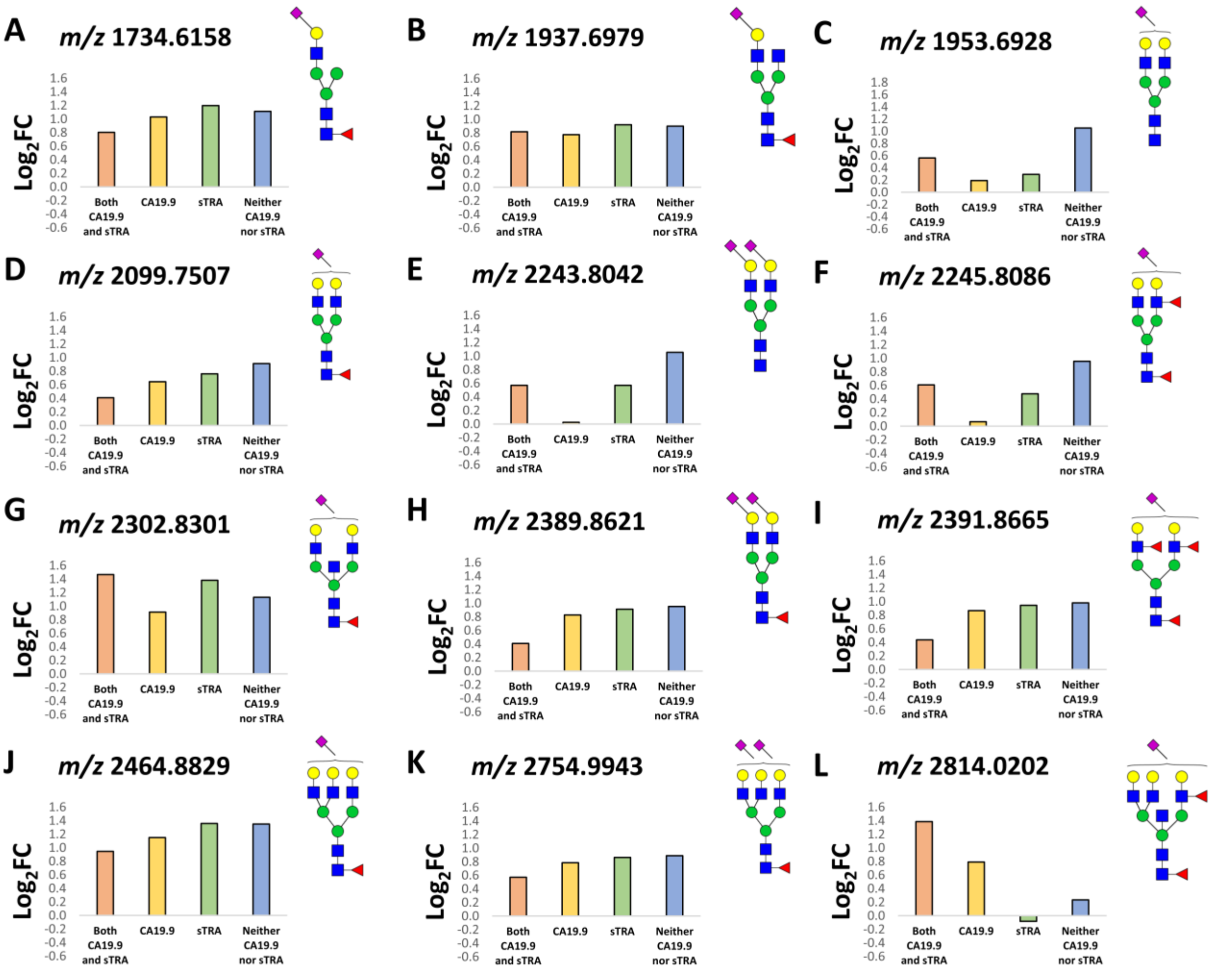

Figure S2. Sialic Acid Stabilization of TMA cores. A-L) Representative α2,3-sialylated N-glycan structures enriched in tumor tissue cores as compared to patient matched normal tissue cores. Log_2 FC between patient matched tumor and normal cores, where positive values represent enrichment in tumor tissue.

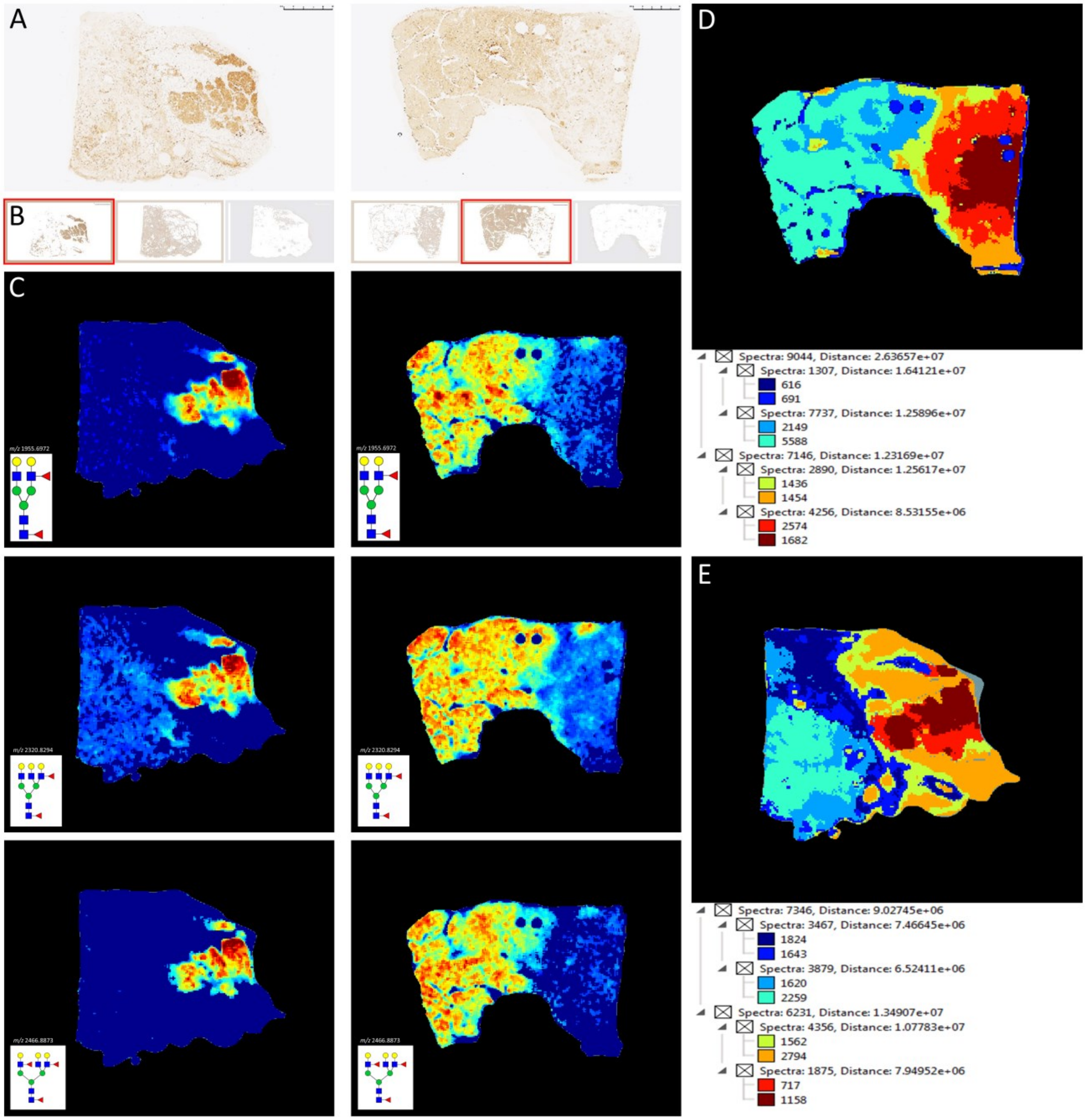

Figure S3. N-glycan colocalization with CA19-9. A) Representative whole tissue block sections from which TMA cores were derived were IHC stained for CA19-9 expression. B) A color-based k-means clustering algorithm was applied to high-resolution staining images to generate unbiased staining classifications, the most intense of which (outlined in red) were co-registered with original scans in Fleximaging and used to draw regions of interest around stained tissue. C) SCiLS lab software was used to identify and create images of N-glycans colocalized to areas of CA19-9 staining. Shown are

three representative N-glycans (Hex5dHex2HexNAc4 *m/z* 1955.6975, Hex6dHex2HexNAc5 *m/z* 2320.8294, Hex6dHex3HexNAc5 *m/z* 2466.8873). D, E) Segmentation analysis across eight nodes of 72 N-glycan structures showing differential associations of glycans between CA19-9-staining and non-CA19-9-staining tissue.

TB#	Race	Sex	Age	Vital Status	Recurrence	Type of Recurrence	Days between Surgery and Recurrence	Days between Surgery and Date of last contact/Death	Histological Type	Histological Grade	Staging
2204	WHITE	M	75	Unknown	Unknown	N/A	N/A	272	Adenocarcinoma	moderately differentiated	T3NOMX
2278	WHITE	M	76	Unknown	Unknown	N/A	N/A	211	cholangiocarcinoma	moderately differentiated	T3N1MX
2279	WHITE	F	61	Unknown	YES	distant recurrence - GAST	2233	2486	Adenocarcinoma	well differentiated	T3NOMX
2381	WHITE	M	73	Unknown	YES	Distant recurrence - CNS	2499	2587	Mucinous Adenocarcinoma	Not Reported	
2417	WHITE	M	80	Unknown	Unknown	N/A	N/A	23	Adenocarcinoma	poorly differentiated	T3N1MX
2454	WHITE	F	69	Unknown	Unknown	N/A	N/A	Unknown	Adenocarcinoma	moderately differentiated	T3NOMX
2487	BLACK	M	73	Unknown	Unknown	N/A	N/A	106	Adenocarcinoma	well differentiated	T3N1MX
2464	BLACK	F	61	Unknown	YES	metasticized to liver and omentum	N/A	Unknown	Adenocarcinoma	poorly differentiated	T4N1Mx
1751	WHITE	M	65	Unknown	Unknown	N/A	N/A	101	Adenocarcinoma	well differentiated	T3N1MX
1800	BLACK	F	67	Unknown	Unknown	N/A	N/A	204	Adenocarcinoma	poorly differentiated	T3N1MX
1691	WHITE	M	69	Unknown	Unknown	N/A	N/A	92	Adenocarcinoma	Not Reported	T3N1MX
1848	WHITE	M	71	Unknown	NO	Never disease free	N/A	314	Adenocarcinoma	poorly differentiated	T3N1MX
1911	WHITE	M	83	Unknown	NO	Disease free	N/A	118	Adenocarcinoma	moderately differentiated	T3N1MX
1747	WHITE	F	63	Unknown	NO	Possible recurrence	N/A	138	Adenocarcinoma	poorly differentiated	T3N1MX
1673	WHITE	M	78	Unknown	NO	Disease free	N/A	440	Adenocarcinoma	moderately differentiated	T3N1
1685	BLACK	M	66	Unknown	NO	Never disease free	N/A	173	Adenocarcinoma	poorly differentiated	T3N1MX
1987	WHITE	F	58	Unknown	NO	Disease free	N/A	404	Adenocarcinoma	moderately differentiated	T3N1MX
2055	WHITE	M	69	Unknown	Unknown	N/A	N/A	808	Adenocarcinoma	poorly differentiated	T3NXMX
1955	BLACK	F	51	Unknown	Unknown	N/A	N/A	392	Adenocarcinoma	moderately differentiated	T3N1MX
1958	WHITE	M	42	Unknown	Unknown	N/A	N/A	429	Adenocarcinoma	poorly differentiated	T3N1MX
1982	WHITE	F	66	Unknown	Unknown	N/A	N/A	336	Adenocarcinoma	moderately differentiated	T2N1MX
2572	WHITE	F	87	Unknown	NO	Disease free	N/A	1387	Adenocarcinoma	moderately differentiated	T1NOMX
2091	WHITE	F	54	Unknown	YES	Local Recurrence	406	547	Adenocarcinoma	Not Reported	T2NOMX
2495	WHITE	M	56	Dead	NO	Never disease free	N/A	76	Adenocarcinoma	Not Reported	T3N1MX
2807	BLACK	F	62	Unknown	NO	Never disease free	N/A	126	Adenocarcinoma	poorly differentiated	T3NOMX
2815	WHITE	F	76	Unknown	Unknown	N/A	N/A	Unknown	Ductal adenocarcinoma	moderately differentiated	T3NOMX
2893	BLACK	F	68	Unknown	YES	Local recurrence	613	613	Adenocarcinoma	moderately differentiated	T3N1MX
2916	WHITE	F	37	Unknown	YES	Distant recurrence	165	236	Adenocarcinoma	moderately differentiated	T3N1MX
2790	WHITE	M	78	Unknown	NO	Never disease free	N/A	32	Adenocarcinoma	moderately differentiated	T2N1bMX
2695	WHITE	M	70	Unknown	NO	Never disease free	N/A	185	Adenocarcinoma	Not Reported	T2NOMX
3721	WHITE	F	66	Unknown	NO	Never diease free	N/A	50	Ductal adenocarcinoma	poorly differentiated	T3N0MX
3627	BLACK	F	84	Unknown	Unknown	N/A	N/A	Unknown	Adenocarcinoma	moderately differentiated	T2N1MX
3508	WHITE	M	49	Unknown	NO	Never disease free	N/A	221	Adenocarcinoma	moderately differentiated	T3N1MX
3727	WHITE	M	74	Unknown	YES	Distant recurrence	140	140	Adenosquamous carcinoma	moderately differentiated	T3NOMX
3499	WHITE	F	66	Unknown	NO	Disease free	N/A	125	Ductal adenocarcinoma	Not Reported	T1NOMX
3463	WHITE	F	64	Unknown	NO	Disease free	N/A	215	Ductal adenocarcinoma	moderately differentiated	T3N1MX
3832	WHITE	M	68	Unknown	YES	Distant recurrence - LUNG	332	353	Adenocarcinoma	moderately differentiated	T3N1MX
3978	WHITE	M	63	Unknown	NO	Disease free	N/A	25	Adenocarcinoma	Not Reported	T2N1bMX
3922	BLACK	M	72	Unknown	NO	Disease free	N/A	94	Ductal adenocarcinoma	moderately differentiated	T1NOMX
3986	WHITE	F	70	Unknown	NO	Disease free	N/A	27	Adenocarcinoma	poorly differentiated	T3N1MX
3406	WHITE	F	74	Unknown	NO	Disease free	N/A	62	Ductal adenocarcinoma	moderately differentiated	T3N1MX
4038	WHITE	M	71	Unknown	YES	Distant recurrence - LUNG	525	318	Adenocarcinoma	moderately differentiated	T3N1MX
3859	WHITE	F	50	Dead	YES	Distant recurrence - HEPT	196	202	Ductal adenocarcinoma	moderately differentiated	T3N1MX
3915	WHITE	F	53	Dead	YES	Distant recurrence - LUNG	1182	1213	Ductal adenocarcinoma	moderately differentiated	T2N0MX
3891	WHITE	F	62	Unknown	Unknown	N/A	N/A	1148	Ductal adenocarcinoma	moderately differentiated	T3N1bM
3886	WHITE	M	62	Unknown	YES	Distant recurrence - HEPT	337	604	Adenocarcinoma	moderately differentiated	T3N1bMX
4072	WHITE	F	71	Unknown	NO	Disease Free	N/A	27	Ductal adenocarcinoma	moderately differentiated	T3N0MX
4524	WHITE	F	60	Unknown	NO	Disease Free	N/A	130	Ductal adenocarcinoma	moderately differentiated	T3N1MX
4071	BLACK	M	64	Unknown	NO	Disease Free	N/A	43	Ductal adenocarcinoma	moderately differentiated	T1N1bMX
4365	WHITE	F	78	Unknown	NO	Disease Free	N/A	90	Adenocarcinoma	moderately differentiated	T1N0MX
4180	WHITE	M	87	Unknown	NO	Disease Free	N/A	69	Adenocarcinoma	poorly differentiated	T3N1bMX
4374	BLACK	M	51	Unknown	YES	Distant recurrence - HEPT	735	736	Ductal adenocarcinoma	moderately differentiated	T3N1MX
4257	ASIAN	M	39	Dead	NO	Never disease free	N/A	9	Ductal adenocarcinoma	moderately differentiated	T3N1MX

Table S1. Clinical information for patient samples used for TMA cores and for whole tissue analysis.

Composition	Theoretical Mass	Observed Mass	Mass Error	Composition	Theoretical Mass	Observed Mass	Mass Error	Composition	Theoretical Mass	Observed Mass	Mass Error
Hex3dHex1HexNAc2 + Na	1079.3749	1079.3735	1.2961	Hex5HexNAc5(2,6)NeuAc1 + Na	2184.8035	2184.8077	1.9224	Hex6dHex4HexNAc5(2,3)NeuAc1 + Na	2903.0566	2903.0591	0.8612
Hex3HexNAc3 + Na	1136.3964	1136.3971	0.6169	Hex5dHex1HexNAc6 + Na	2215.7980	2215.7952	1.2637	Hex8dHex1HexNAc7 + Na	2906.0393	2906.0507	3.9229
Hex5HexNAc2 + Na	1257.4226	1257.4224	0.1583	Hex6HexNAc6 + Na	2232.7964	2232.7902	2.7589	Hex7dHex4HexNAc6 + Na	2979.0808	2979.0835	0.9063
Hex3dHex1HexNAc3 + Na	1282.4543	1282.4522	1.6367	Hex3dHex2HexNAc6 + Na + 2S	2242.6469	2242.6535	2.9430	Hex6dHex1HexNAc6(2,3)NeuAc1(2,6)NeuAc1 + Na	2986.1050	2986.1060	0.3349
Hex4HexNAc3 + Na	1298.4492	1298.4495	0.2318	Hex5HexNAc4(2,3)NeuAc2 + Na	2243.8042	2243.8000	1.8718	Hex6dHex2HexNAc7(2,3)NeuAc1 + Na	3017.0996	3017.1001	0.1657
Hex6HexNAc2 + Na	1419.4754	1419.4769	1.0574	Hex5dHex2HexNAc4(2,3)NeuAc1 + Na	2245.8086	2245.8094	0.3562	Hex7dHex3HexNAc7 + Na	3036.1023	3036.1050	0.8893
Hex4dHex1HexNAc3 + Na	1444.5071	1444.5063	0.5531	Hex5HexNAc4(2,3)NeuAc1(2,6)NeuAc1 + Na	2271.8355	2271.8445	3.9616	Hex6dHex2HexNAc7(2,6)NeuAc1 + Na	3045.1309	3045.1407	3.2183
Hex5HexNAc3 + Na	1460.5020	1460.5026	0.4115	Hex4dHex2HexNAc5(2,3)NeuAc1 + Na	2286.8352	2286.8368	0.6997	Hex6dHex3HexNAc5(2,3)NeuAc2 + Na	3047.1101	3047.0993	3.5443
Hex3dHex1HexNAc4 + Na	1485.5337	1485.5338	0.0680	Hex5dHex1HexNAc5(2,3)NeuAc1 + Na	2302.8301	2302.8257	1.9107	Hex8HexNAc7(2,3)NeuAc1 + Na	3049.0894	3049.0859	1.1479
Hex4HexNAc4 + Na	1501.5286	1501.5282	0.2657	Hex5dHex3HexNAc5 + Na	2304.8345	2304.8318	1.1710	Hex8dHex1HexNac8 + Na	3109.1187	3109.1186	0.0322
Hex3HexNAc5 + Na	1542.5552	1542.5536	1.0366	Hex6HexNAc5(2,3)NeuAc1 + Na	2318.8250	2318.8205	1.9406	Hex7dHex1HexNAc6(2,3)NeuAc2 + Na	3120.1265	3120.1198	2.1473
Hex7HexNAc2 + Na	1581.5282	1581.5320	2.4034	Hex6dHex2HexNAc5 + Na	2320.8294	2320.8242	2.2401	Hex7dHex3HexNAc6(2,3)NeuAc1 + Na	3122.1309	3122.1277	1.0249
Hex4HexNAc3(2,3)NeuAc1 + Na	1588.5606	1588.5557	3.0846	Hex5dHex1HexNAc5(2,6)NeuAc1 + Na	2330.8614	2330.8532	3.5180	Hex9HexNAc8 + Na	3124.1353	3124.1252	3.2329
Hex5dHex1HexNAc3 + Na	1606.5599	1606.5528	4.4188	Hex6HexNAc5(2,6)NeuAc1 + Na	2346.8563	2346.8634	3.0253	Hex6HexNAc6(2,3)NeuAc2(2,6)NeuAc1 + Na	3130.1585	3130.1478	3.4184
Hex4dHex1HexNAc4 + Na	1647.5865	1647.5862	0.1815	Hex6dHex1HexNAc6 + Na	2377.8509	2377.8482	1.1351	Hex6dHex2HexNAc6(2,3)NeuAc1(2,6)NeuAc1 + Na	3132.1629	3132.1748	3.7993
Hex5HexNAc4 + Na	1663.5814	1663.5831	1.0225	Hex5HexNAc6(2,6)NeuAc1 + Na	2387.8828	2387.8879	2.1358	Hex7dHex2HexNAc7(2,3)NeuAc1 + Na	3179.1524	3179.1513	0.3460
Hex3dHex1HexNAc5 + Na	1688.6131	1688.6128	0.1771	Hex5dHex1HexNAc4(2,3)NeuAc2 + Na	2389.8621	2389.8575	1.9248	Hex8dHex3HexNAc7 + Na	3198.1551	3198.1541	0.3127
Hex4HexNAc5 + Na	1704.6080	1704.6083	0.1766	Hex5dHex3HexNAc4(2,3)NeuAc1 + Na	2391.8665	2391.8600	2.7175	Hex8dHex2HexNAc8 + Na	3255.1766	3255.1788	0.6758
Hex4dHex1HexNAc3(2,3)NeuAc1 + Na	1734.6158	1734.6101	3.2860	Hex7HexNAc6 + Na	2393.8458	2393.8466	0.3346	Hex7HexNAc6(2,3)NeuAc3 + Na	3264.1800	3264.1855	1.6850
Hex8HexNAc2 + Na	1743.5810	1743.5814	0.2300	Hex5dHex1HexNAc4(2,3)NeuAc1(2,6)NeuAc1 + Na	2417.8934	2417.8946	0.4963	Hex7dHex2HexNAc6(2,3)NeuAc2 + Na	3266.1844	3266.1844	0.0000
Hex5HexNAc3(2,3)NeuAc1 + Na	1750.6134	1750.6177	2.4563	Hex5dHex3HexNAc4(2,6)NeuAc1 + Na	2419.8978	2419.9008	1.2397	Hex7dHex4HexNAc6(2,3)NeuAc1 + Na	3268.1888	3268.1996	3.3046
Hex4HexNAc4(2,3)NeuAc1 + Na	1791.6489	1791.6489	0.0000	Hex5dHex2HexNAc5(2,3)NeuAc1 + Na	2448.8880	2448.8986	4.3285	Hex9dHex1HexNAc8 + Na	3270.1681	3270.1758	2.3549
Hex5dHex1HexNAc4 + Na	1809.6393	1809.6394	0.0558	Hex6dHex1HexNAc5(2,3)NeuAc1 + Na	2464.8829	2464.8741	3.5701	Hex7dHex1HexNAc7(2,3)NeuAc2 + Na	3323.2059	3323.2070	0.3310
Hex4dHex1HexNAc5 + Na	1850.6659	1850.6646	0.7019	Hex6dHex3HexNAc5 + Na	2466.8873	2466.8915	1.7030	Hex7dHex3HexNAc7(2,3)NeuAc1 + Na	3325.2103	3325.2053	1.5037
Hex5HexNAc5 + Na	1866.6608	1866.6627	1.0184	Hex5HexNAc5(2,3)NeuAc1(2,6)NeuAc1 + Na	2474.9149	2474.9121	1.1314	Hex7dHex5HexNAc7 + Na	3328.2181	3328.2107	2.2234
Hex3dHex1HexNAc6 + Na	1891.6925	1891.6903	1.1625	Hex5dHex3HexNAc6 + Na	2507.9139	2507.9015	4.9443	Hex6dHex1HexNAc6(2,6)NeuAc3 + Na	3332.2790	3332.2829	1.1704
Hex9HexNAc2 + Na	1905.6338	1905.6345	0.3679	Hex6dHex2HexNAc6 + Na	2523.9088	2523.9113	0.9909	Hex7dHex1HexNAc6(2,3)NeuAc3 + Na	3410.2379	3410.2364	0.4399
Hex5dHex1HexNAc4 + 2Na + S	1911.5859	1911.5781	4.0636	Hex7dHex1HexNAc6 + Na	2539.9037	2539.9033	0.1571	Hex7dHex2HexNAc7(2,3)NeuAc2 + Na	3469.2638	3469.2783	4.1796
Hex6HexNAc3(2,3)NeuAc1 + Na	1912.6662	1912.6619	2.2482	Hex6dHex1HexNAc7 + Na	2581.9336	2581.9336	0.0155	Hex8dHex1HexNAc7(2,3)NeuAc2 + Na	3485.2587	3485.2551	1.0329
Hex4dHex1HexNAc4(2,3)NeuAc1 + Na	1937.6979	1937.6928	2.6320	Hex5dHex1HexNAc5(2,3)NeuAc2 + Na	2592.9415	2592.9445	1.1570	Hex8dHex3HexNAc7(2,3)NeuAc1 + Na	3487.2631	3487.2583	1.3764
Hex4dHex1HexNAc5 + Na + S	1952.6125	1952.6059	3.3801	Hex5dHex3HexNAc5(2,3)NeuAc1 + Na	2594.9459	2594.9536	2.9673	Hex8dHex2HexNAc8(2,3)NeuAc1 + Na	3544.2846	3544.2935	2.5111
Hex5HexNAc4(2,3)NeuAc1 + Na	1953.6928	1953.6928	0.0000	Hex7HexNAc7 + Na	2596.9252	2596.9269	0.6550	Hex7dHex2HexNAc6(2,3)NeuAc3 + Na	3556.3210	3556.3148	1.7434
Hex5dHex2HexNAc4 + Na	1955.6972	1955.6959	0.6642	Hex6HexNAc5(2,3)NeuAc2 + Na	2608.9364	2608.9475	4.2546	Hex7dHex4HexNAc6(2,3)NeuAc2 + Na	3558.3002	3558.3117	3.2319
Hex3dHex1HexNAc6 + Na + S	1993.6390	1993.6337	2.6585	Hex6dHex2HexNAc5(2,3)NeuAc1 + Na	2610.9408	2610.9421	0.4979	Hex9dHex1HexNAc8(2,3)NeuAc1 + Na	3560.2795	3560.2885	2.5279
Hex4HexNAc5(2,3)NeuAc1 + Na	1994.7193	1994.7098	4.7626	Hex6dHex4HexNAc5 + 1Na	2613.9486	2613.9419	2.5632	Hex9dHex1HexNAc8(2,6)NeuAc1 + Na	3588.3108	3588.3089	0.5295
Hex4dHex2HexNAc5 + Na	1996.7238	1996.7235	0.1502	Hex6dHex1HexNAc6(2,3)NeuAc1 + Na	2667.9623	2667.9623	0.0000	Hex8dHex4HexNAc7(2,3)NeuAc1 + Na	3633.3210	3633.3287	2.1193
Hex5dHex1HexNAc5 + Na	2012.7187	2012.7180	0.3473	Hex6dHex3HexNAc6 + Na	2669.9667	2669.9796	4.8319	Hex10dHex1HexNAc9 + Na	3636.3037	3636.2973	1.7600
Hex4HexNAc5(2,6)NeuAc1 + Na	2022.7506	2022.7546	1.9775	Hex7dHex2HexNAc6 + Na	2685.9616	2685.9595	0.7815	Hex7dHex4HexNAc8(2,3)NeuAc1 + Na	3674.3476	3674.3471	0.1361
Hex6HexNAc5 + Na	2028.7136	2028.7158	1.0849	Hex7dHex1HexNAc7 + Na	2742.9831	2742.9857	0.9482	Hex7dHex3HexNAc6(2,3)NeuAc3 + Na	3702.3789	3702.3866	2.0797
Hex5dHex2HexNAc4 + 2Na + S	2057.6438	2057.6533	4.6325	Hex6dHex1HexNAc5(2,3)NeuAc2 + Na	2754.9943	2754.9948	0.1815	Hex9HexNAc8(2,3)NeuAc2 + Na	3704.3330	3704.3417	2.3486
Hex6dHex1HexNAc3(2,3)NeuAc1 + Na	2058.7242	2058.7323	3.9345	Hex6dHex3HexNAc5(2,3)NeuAc1 + Na	2756.9987	2756.9994	0.2539	Hex9dHex4HexNAc8 + Na	3709.3452	3709.3412	1.0784
Hex4dHex2HexNAc4(2,3)NeuAc1 + Na	2083.7558	2083.7473	4.0792	Hex6dHex2HexNAc6(2,3)NeuAc1 + Na	2814.0202	2814.0335	4.7263	Hex9dHex2HexNAc8(2,6)NeuAc1 + Na	3734.3687	3734.3618	1.8477
Hex3dHex1HexNAc6 + Na + 2S	2095.5856	2095.5805	2.4337	Hex7dHex1HexNAc6(2,3)NeuAc1 + Na	2830.0151	2830.0192	1.4488	Hex10HexNAc9(2,3)NeuAc1 + Na	3779.3888	3779.3858	0.7938
Hex5dHex1HexNAc4(2,3)NeuAc1 + Na	2099.7507	2099.7501	0.2857	Hex7dHex3HexNAc6 + Na	2832.0195	2832.0124	2.5067	Hex8dHex4HexNAc8(2,3)NeuAc1 + Na	3836.4004	3836.4063	1.5379
Hex5dHex3HexNAc4 + Na	2101.7551	2101.7545	0.2850	Hex6HexNAc6(2,3)NeuAc1(2,6)NeuAc1 + Na	2840.0471	2840.0494	0.8098	Hex9dHex1HexNAc8(2,3)NeuAc2 + Na	3850.3909	3850.3930	0.5454
Hex5dHex1HexNAc4(2,6)NeuAc1 + Na	2127.7820	2127.7791	1.3629	Hex6dHex2HexNAc6(2,6)NeuAc1 + Na	2842.0515	2842.0592	2.7093	Hex9dHex3HexNAc8(2,3)NeuAc1 + Na	3852.3953	3852.4069	3.0111
Hex3dHex2HexNAc6 + Na + S	2139.6969	2139.6970	0.0467	Hex7dHex1HexNAc6(2,6)NeuAc1 + Na	2858.0464	2858.0526	2.1693	Hex9dHex5HexNAc8 + Na	3855.4031	3855.3881	3.8906
Hex4dHex1HexNAc5(2,3)NeuAc1 + Na	2140.7773	2140.7724	2.2889	Hex6dHex3HexNAc7 + Na	2873.0461	2873.0331	4.5245	Hex9dHex3HexNAc8(2,6)NeuAc1 + Na	3880.4266	3880.4128	3.5563
Hex5HexNAc5(2,3)NeuAc1 + Na	2156.7722	2156.7735	0.6028	Hex7dHex2HexNAc7 + Na	2890.0444	2890.0463	0.6574	Hex8dHex5HexNAc8(2,3)NeuAc1 + Na	3982.4583	3982.4489	2.3604
Hex5dHex2HexNAc5 + Na	2158.7766	2158.7746	0.9260	Hex6dHex1HexNAc7(2,6)NeuAc1 + Na	2899.0729	2899.0739	0.3449				
Hex6dHex1HexNAc5 + Na	2174.7715	2174.7747	1.4719	Hex6dHex2HexNAc5(2,3)NeuAc2 + Na	2901.0522	2901.0474	1.6546				

Table S2. Composition and mass accuracy of 151 N-glycan structures obtained via MALDI-IMS analysis. All detected structures are monoisotopic, underivatized and singly-sodiated unless otherwise indicated. Sialic acid derivatization by amidation amidation (AA) reaction yields a mass difference of +290.1114 for 2,3 linked sialic acid residues and +318.1427 for 2,6 linked sialic acid residues. Mass error calculation: ((ABS(Theoretical *m/z* – Observed *m/z*)/Theoretical *m/z*)*1,000,000).

m/z	Structure	m/z	Structure	m/z	Structure	m/z	Structure	m/z	Structure	m/z	Structure
1079.3745*		1905.6338		2215.7980*		2539.9037*		2979.0808*		3485.2587	
1136.3964		1911.5859*		2232.7964		2581.9336		2986.1050		3487.2631	
1257.4226		1912.6662		2242.6469*		2592.9415		3017.0996		3544.2846	
1282.4543*		1937.6979		2243.8042		2594.9459		3036.1023*		3556.3210	
1298.4492		1952.6125*		2245.8086		2596.9252		3045.1309		3558.3002	
1419.4754		1953.6928		2271.8355		2608.9364		3047.1101		3560.2795	
1444.5071*		1955.6972*		2286.8352		2610.9408		3049.0894		3588.3108	
1460.5020		1993.6390*		2302.8301		2613.9486		3109.1187*		3633.3210	
1485.5337*		1994.7193		2304.8345*		2667.9623		3120.1265		3636.3037	
1501.5286		1996.7238*		2318.8250		2669.9667*		3122.1309		3674.3476	
1542.5552		2012.7187*		2320.8294*		2685.9616*		3124.1353*		3702.3789	
1581.5282		2022.7506		2330.8614		2742.9831*		3130.1585		3704.3330	
1588.5606		2028.7136		2346.8563		2754.9943		3132.1629		3709.3452	
1606.5599*		2057.6438		2377.8509*		2756.9987		3179.1524		3734.3687	
1647.5865*		2058.7242		2387.8828		2814.0202		3198.1551*		3779.3888	
1663.5814		2083.7558		2389.8621		2830.0151		3255.1766*		3836.4004	
1688.6131*		2095.5856*		2391.8665		2832.0195*		3264.1800		3850.3909	
1704.6080		2099.7507		2393.8458		2840.0471		3266.1844		3852.3953	
1734.6158		2101.7551*		2417.8934		2842.0515		3268.1888		3855.4031	
1743.5810		2127.7820		2419.8978		2858.0464		3270.1681*		3880.4266	
1750.6134		2139.6969*		2448.8880		2873.0461*		3323.2059		3982.4583	
1791.6489		2140.7773		2464.8829		2890.0444*		3325.2103			
1809.6393*		2156.7722		2466.8873*		2899.0729		3328.2181*			
1850.6659*		2158.7766*		2474.9149		2901.0522		3332.2790			
1866.6608		2174.7715*		2507.9139*		2903.0566		3410.2379			
1891.6925*		2184.8035		2523.9088*		2906.0393*		3469.2638			

Galactose (Hex)
Mannose (Hex)
N-acetylglucosamine (HexNAc)
N-acetylgalactosamine (HexNAc)
Fucose (dHex)
2,3-Sialic Acid (2,3NeuAc)
2,6-Sialic Acid (2,6NeuAc)

Table S3. Summary table of N-glycan structures cumulatively detected across all analyzed tissues. A key to the monosaccharide unit symbols is provided in the bottom right. N-glycan structures denoted with a * indicates that the presence of a core fucose reside has been confirmed via analysis

using Endo F3. It is important to note that the representative structures provided do not encompass all possible isomeric conformations for these N-glycans, but are the most likely based on previous reporting, orthogonal analyses and the available biosynthetic pathways.

	Both CA19.9 and sTRA		CA19.9		sTRA		Neither CA19-9 nor sTRA	
Centroid [*m/z*]	Fold Change	Intensity	Fold Change	Intensity	Fold Change	Intensity	Fold Change	Intensity
1079.3749	0.5580	1030.2615	0.5620	1013.5629	0.7529	799.8649	0.0691	681.6336
1136.3964	0.6459	676.5595	0.3493	637.1259	0.7473	578.7372	0.7534	556.2163
1257.4226	0.1402	2654.0624	-0.0147	2351.0033	0.3208	2490.4892	0.0796	2039.8801
1282.4543	0.3096	2313.2134	0.3698	2365.8460	0.6335	2372.4875	0.3087	1953.0532
1298.4492	0.2279	3618.3780	0.0596	3606.5293	0.4242	3475.8308	0.5033	3542.7250
1419.4754	-0.0708	4435.2276	0.0448	4359.8460	-0.0115	4282.9775	-0.0462	4038.3505
1444.5071	0.2024	6781.2728	0.2931	7440.2953	0.6529	7254.8175	0.4100	6397.8605
1460.5020	0.4495	1261.6067	0.2437	1421.0725	0.8493	1163.6526	0.5832	1078.5609
1485.5337	0.3397	4496.9126	0.1656	4925.8533	0.7496	4141.5008	0.5373	3847.4355
1501.5286	0.4488	3504.0746	0.1823	3252.7713	0.5833	3217.3858	0.6136	3322.8205
1542.5552	0.6699	631.8115	0.0037	479.5078	0.5234	704.8998	0.2051	472.3477
1581.5282	0.2772	3565.8188	0.2526	3591.4847	0.3927	2961.3900	0.1304	2688.9080
1606.5599	0.3671	940.9854	0.4849	1080.3293	0.9305	1025.6925	0.6006	931.9258
1647.5865	0.2576	10790.2408	0.2137	11085.4327	0.4937	11279.3008	0.3803	11031.1855
1663.5814	0.2230	47111.1360	0.0303	48719.1733	0.4207	45885.2833	0.5582	48841.2200
1688.6131	0.0528	2952.7942	-0.2663	2889.9897	0.5822	3677.0442	0.0743	2442.0707
1704.6080	0.9186	2452.2669	0.3307	2116.1872	1.1304	1949.8679	0.8319	1476.5198
1743.5810	-0.0583	8841.7870	0.0148	8836.4647	-0.0550	7861.5800	-0.1055	7811.1225
1791.6489	0.4449	3437.4208	0.5246	4130.0147	1.1472	2999.8529	0.5074	2359.7695
1809.6393	0.2343	126072.3760	0.3589	145564.3267	0.7564	141867.2083	0.5121	119715.5000
1850.6659	0.4498	13157.5664	0.1981	13928.0233	0.8493	14265.8200	0.5151	11948.0795
1866.6608	0.6915	5408.9500	0.3092	4009.2933	0.8292	4745.9275	0.6856	5016.9438
1891.6925	0.0593	771.5054	0.2295	1007.8143	-0.1452	546.2488	-0.7741	468.9750
1905.6338	-0.3614	4891.3512	-0.1989	5941.8553	-0.0484	4008.0575	-0.1055	4541.7310
1911.5859	0.0774	9652.6898	-0.2242	10247.3253	0.2193	9037.3833	0.6677	11668.2585
1955.6972	-0.2590	7289.5402	-0.0843	7869.6207	-0.0750	6896.1758	-0.3268	6893.2070
1996.7238	0.8381	4608.1824	0.6761	3736.5147	0.5950	3428.9295	-0.2268	2940.5792
2012.7187	0.4143	43498.1460	0.3009	45450.9400	0.9035	51220.2250	0.4612	38769.4650
2028.7136	0.4012	9839.5872	0.2221	10008.1940	0.7816	9078.7983	0.8338	9383.0765
2057.6438	0.0535	18426.9780	0.1073	21937.0833	0.4448	20046.7167	0.5167	21521.1305
2101.7551	-0.0692	7501.7748	0.0102	8503.1773	0.6562	7969.7775	0.2302	7388.0085
2158.7766	0.4011	11363.7478	0.3342	9282.0100	-0.0345	7989.2683	-0.5551	7531.1735
2174.7715	0.2815	53484.7100	0.4252	57246.1800	0.7232	64477.1917	0.5321	59652.0200
2215.7980	0.9590	4382.5464	0.5851	4778.4880	1.2673	3955.2642	0.4642	3491.0819
2232.7964	0.9598	4320.7963	0.8038	3384.2840	1.4892	2737.8392	0.8888	2981.5574
2304.8345	0.8943	6529.6180	0.9264	6704.9727	0.5532	3284.2436	-0.2947	3249.5261
2320.8294	-0.1965	6459.7967	-0.1060	6053.7300	0.1800	5034.5917	-0.4141	5075.9610
2377.8509	0.4545	16636.5238	0.2718	14331.7167	0.9078	18517.2333	0.2226	17797.5590
2393.8458	0.5722	4319.5450	0.4443	4369.4367	1.2084	3761.7533	1.2048	3747.1576
2466.8873	-0.2042	8261.3122	-0.1177	8194.9387	0.2008	8662.9767	-0.3248	8401.9900
2507.9139	0.6044	2482.9564	0.8787	2573.6232	-0.4874	1244.8233	-0.7505	1571.1613
2523.9088	0.6238	8240.1160	0.4511	6913.1740	0.5957	5074.2350	-0.5965	4028.8324
2539.9037	0.0839	34950.5440	0.2730	34169.4800	0.5440	47457.7000	0.3586	46961.3775
2581.9336	0.9281	3156.8733	0.4690	3384.8950	0.8477	2477.0816	0.2002	3008.1229
2613.9486	-0.5574	1040.7924	-0.1643	1097.4909	-1.0460	1052.5307	-1.6169	1427.1412
2669.9667	1.2122	3157.0727	0.8713	3087.5575	0.5335	865.8589	-0.6530	927.7440
2685.9616	-0.3670	6207.3348	-0.2400	5973.3425	-0.0170	5614.4558	-0.6943	5776.5140
2742.9831	0.9924	11120.2304	0.8345	10064.4913	1.9265	8673.5875	0.5077	5522.0960
2832.0195	-0.3598	7215.8054	-0.3907	6264.5160	-0.0894	7355.2933	-0.6649	7728.5317
2890.0444	1.1449	5095.1726	0.4796	4355.5224	1.0930	1939.7393	-0.6336	1792.3854
2906.0393	0.3592	13588.1608	0.5779	16725.3580	1.1644	16365.6783	1.0149	14296.7880
2979.0808	-0.7050	2110.1836	-0.8280	3538.4809	-0.7391	1756.5904	-1.4827	2281.3808
3036.1023	0.9367	12185.8834	0.6211	7251.7120	0.2973	6190.2181	-0.7168	5473.1566
3109.1187	0.7600	3619.1301	0.8687	3715.9760	0.6550	3580.1689	0.4216	3847.6254
3124.1102	-0.0703	1367.6939	-0.2554	1744.9258	-0.2194	1498.8243	-0.9417	1624.6381
3198.1551	-0.5800	4070.1716	-0.4382	4077.9780	-0.6499	5322.4083	-0.9959	4366.8126
3255.1766	0.4646	4019.1874	0.3279	3363.3185	0.2530	2510.7031	-0.5447	2158.0608
3270.1681	0.3299	2498.8385	0.7281	3600.9105	1.3078	3232.0292	0.7372	2983.1392
3328.2181	0.9078	6558.7212	1.1305	6332.3680	-0.0396	3531.0183	-0.1763	3367.1860
3636.3037	0.0741	7494.0596	0.4479	8220.9520	0.4143	10278.0333	0.2161	10434.0220
3709.3452	-0.6620	2239.3698	-0.7074	2430.1141	-0.7433	3195.2648	-1.0279	2632.6308
3855.4031	-0.4590	2145.1399	-0.5476	1865.9784	-0.7010	3220.6986	-0.9423	3064.2660

Table S4. Log_2FC and AUP intensity data for biomarker-classified TMA core groupings. Log_2FC of area under the peak (AUP) measurements for 62 N-glycan masses between individual patient normal and tumor cores were calculated then averaged for the 4 biomarker-designations. Positive values represent enrichment in tumor tissue. Shown too are the average tumor AUP intensities for each N-glycan in each category.

	Both CA19.9 and sTRA		CA19.9		sTRA		Neither CA19.9 nor sTRA	
Centroid [m/z]	Fold Change	Intensity	Fold Change	Intensity	Fold Change	Intensity	Fold Change	Intensity
1588.5557	0.7300	878.9225	0.2582	698.8101	0.6013	914.8836	1.0571	900.6399
2099.7507	0.4085	46658.9906	0.6431	45193.2667	0.7600	67360.1333	0.9116	51497.8833
2391.8665	0.4367	34321.2866	0.8654	37454.7827	0.9439	53369.4667	0.9800	36108.7595
2389.8621	0.4071	33339.7756	0.8272	38784.7980	0.9122	49766.4667	0.9518	34549.3119
3045.1309	0.6408	13394.7468	0.6678	12234.5000	0.9185	20410.6367	0.7377	13590.6520
3047.1101	0.6488	12995.0706	0.6338	11794.2727	0.9016	19478.3375	0.8882	13156.2914
2245.8086	0.6080	11617.8172	0.0660	8465.1040	0.4763	12004.9650	0.9556	11237.0686
2754.9943	0.5707	11507.1192	0.7844	11127.6347	0.8623	16962.4208	0.8884	12515.7129
2243.8042	0.5690	11289.2776	0.0248	8271.0900	0.5687	12303.3358	1.0540	11417.7861
2756.9987	0.6774	10354.7216	0.6550	8177.2100	0.8206	14632.9867	0.7978	10887.5210
1953.6928	0.5610	5341.5294	0.1919	4483.8242	0.2918	5800.5642	1.0531	5745.1135
3122.1309	0.5321	5251.0380	0.3164	3755.3627	0.4770	6739.2292	0.5712	5701.2346
3120.1265	0.2387	4072.2387	0.2652	3178.2790	0.5609	5881.9900	0.6059	4716.3866
2302.8301	1.4655	3847.4997	0.9116	3172.0937	1.3803	4229.3554	1.1299	2820.8969
2464.8829	0.9450	3460.7556	1.1502	3142.9455	1.3584	5208.6625	1.3483	3688.6793
1937.6979	0.8157	2947.7337	0.7730	2664.7448	0.9204	3516.7792	0.8982	2645.7965
2330.8614	1.1615	2824.5832	0.3524	2297.8135	0.5762	2525.8255	1.3915	2862.6964
2899.0729	0.5971	2181.1156	-0.4711	1199.2390	0.6073	2430.2258	1.0099	2032.7094
2901.0522	0.7902	2170.4498	-0.2949	1141.1947	0.4682	2071.1514	0.7954	1771.5648
2814.0202	1.3854	1855.9642	0.7914	1118.1365	-0.0829	401.2256	0.2334	549.3123
3179.1524	1.3878	1669.0263	0.4102	1522.2567	0.1445	357.5152	0.0920	512.8538
1734.6158	0.8024	1607.1447	1.0297	1632.8729	1.1968	2150.3284	1.1124	1489.2360
3049.0894	0.5562	1284.9651	0.3277	1123.7731	1.1540	2015.6976	0.9335	1379.2139
2592.9415	1.2570	1263.7443	0.7126	1020.7995	1.1694	1692.1517	0.3048	835.1244
2184.8035	0.9618	1045.4470	1.3543	1027.8778	1.3159	1145.7591	1.4707	1051.8391
1791.6489	0.9779	1043.3444	0.4214	775.6399	0.8273	997.3373	1.0619	842.1190
3325.2103	1.1212	1008.8287	0.2911	1179.1920	-0.1319	256.9930	0.4174	438.9953
2594.9459	1.4722	953.4439	1.0805	656.8822	1.2411	867.6010	0.5377	449.5816
3266.1844	0.3052	952.2453	0.1311	720.6593	-0.0221	715.1896	0.3677	831.5409
2140.7773	1.5996	898.3702	0.9094	820.8087	1.6031	778.4248	1.1731	544.4596
2417.8934	0.7703	885.0449	0.5632	781.6632	0.8685	1023.3772	0.8630	778.8700
2667.9623	1.1839	881.8418	0.6622	906.5518	0.9665	597.8705	0.5209	557.4260
3588.3108	-0.1586	870.4476	-0.0132	771.8786	-0.2820	557.7137	-0.1153	561.1179
3633.3210	0.0963	846.9732	0.1725	731.8269	0.2039	761.6313	0.0689	719.0854
3556.3210	0.0232	801.2463	-0.0855	655.2075	-0.0248	673.1899	-0.0190	668.3081
3702.3789	0.0218	801.0947	-0.1589	584.6486	0.2690	1368.1991	0.1782	749.8388
3734.3687	0.3393	780.6715	0.2635	402.7431	0.1676	307.3056	0.3022	358.2584
3852.3953	0.2559	763.1563	0.6593	675.2545	1.1005	1101.8338	0.3252	928.5617
3880.4266	0.1475	737.3105	0.1582	250.2074	0.3917	243.8357	-0.0014	289.7402
2474.9149	0.8946	686.1145	0.9829	656.6366	1.1113	800.4861	0.9617	629.4493
2083.7558	1.0305	683.1610	0.9682	688.7409	1.0032	819.3805	0.7386	580.6011
3264.1800	0.1508	681.4168	0.1624	586.8056	-0.2936	466.6913	0.0243	587.8730
3558.3002	0.0887	650.5435	0.3413	448.1070	-0.1998	377.3892	-0.2380	445.9021
3017.0996	0.5944	628.9637	0.2463	897.2575	-0.1218	355.5194	0.2646	483.1616
3268.1888	0.3009	619.2407	0.0105	370.1782	-0.2830	310.6225	-0.0844	513.8394
3779.3888	0.0072	618.9588	-0.0012	491.1958	0.0765	590.6224	0.0306	592.2058
2271.8355	0.6132	595.1615	0.0929	578.5817	0.3732	457.5226	1.5522	689.6138
3544.2846	0.2542	594.1858	0.1111	567.3241	-0.4338	305.4741	-0.0415	332.0953
2986.1050	0.2106	585.3374	-0.5972	292.6053	0.0032	404.1517	0.2094	431.8489
2318.8250	0.6538	584.3362	0.3338	398.4857	0.5436	533.5258	0.9121	536.5304
3982.4489	0.4478	580.6259	-0.2800	425.2241	0.1226	352.2969	-0.3154	303.5218
3560.2795	0.3271	507.9753	0.1348	440.5444	0.1861	399.4267	0.0528	425.8965
2903.0591	0.4282	501.0887	0.0034	292.1498	0.0105	344.4316	-0.0149	318.7844
2830.0151	0.3095	497.6539	0.0635	333.8733	0.5711	682.2370	0.3720	670.9259
3836.4004	0.4564	485.8277	-0.2249	372.0839	0.0272	213.7607	-0.0277	227.1791
2858.0464	0.3286	471.1037	0.1159	399.8335	0.2109	351.9727	0.4582	408.8395
3332.2790	-0.2879	467.4517	-0.1936	555.6265	-0.3919	420.6194	-0.2819	452.3097
2156.7722	1.1207	463.3966	0.3849	198.9112	0.3225	211.8881	0.4691	176.1748
3674.3476	-0.0949	461.6950	0.4208	617.0365	0.0559	382.7484	0.0033	377.7417
3469.2638	0.3234	451.8388	0.2901	557.1624	0.1247	307.3554	-0.3367	235.0908
2419.8978	0.7187	438.1490	1.0715	691.8955	0.8272	399.9938	0.6140	341.8993
2840.0471	0.1858	435.9853	-0.1140	342.0754	-0.0157	372.8133	0.1023	411.9397
3132.1629	0.5202	414.1247	-0.2280	291.2164	0.2698	349.5046	0.4708	379.7658
2286.8352	1.2124	387.2843	0.7412	321.8799	0.6546	207.9919	0.0488	172.4521
3850.3909	0.0671	381.4620	-0.2276	292.1214	0.3974	414.4407	0.4601	430.5678
3485.2587	-0.0369	355.2222	0.3044	390.2540	0.6817	502.0180	0.2904	398.1496
2387.8828	0.4619	353.3201	0.6471	476.2327	1.1563	356.8234	0.4037	255.9164
3487.2631	0.0582	348.7592	-0.2127	280.0704	0.3030	414.5090	0.5492	425.8026
2448.8880	1.2145	346.3911	1.2166	203.4291	0.6341	124.8313	0.1787	104.3229
1912.6662	0.6200	338.5660	0.2897	223.6775	0.0208	266.9027	0.5936	271.3491
3704.3330	0.3689	334.1641	-0.0561	254.6241	0.0847	368.9007	0.1781	370.4934
3323.2059	0.3631	331.7865	0.3741	598.2298	-0.2183	262.0051	0.0551	305.2166
2022.7506	0.5885	283.7597	0.5043	262.4727	0.7844	332.7603	0.7320	276.5223
3410.2379	0.0387	278.9485	0.3709	243.1178	0.4703	363.7577	0.0509	260.5690
3130.1585	0.2613	277.6546	0.3256	240.1254	0.1284	210.9380	0.0178	239.1858
2610.9408	0.0568	217.1047	0.1058	147.2209	-0.4102	126.9894	-0.1188	148.4337
2842.0515	0.0351	204.7200	-0.0433	159.2148	-0.1203	190.2229	0.0270	191.5226
1994.7193	0.8127	197.2779	0.5334	157.6480	0.0055	119.7286	0.3797	115.8698
2346.8563	0.3056	190.8673	0.5525	219.4622	0.5506	168.0192	0.5498	194.9802
2058.7242	0.5841	187.8892	0.6469	125.5378	-0.2552	90.8619	0.3214	111.9037
2608.9364	0.1552	186.2921	-0.1916	110.5371	0.1005	178.4843	0.2861	184.8986
1750.6134	0.4084	172.3391	0.0769	142.8973	0.0794	146.7574	0.4893	159.4521
2101.7551	0.3837	126.9926	0.7124	143.5501	0.0491	130.0193	0.6879	128.6026

Table S5. Log_2FC and AUP intensity data for AA-stabilized TMA cores. Log_2FC of area under the peak (AUP) measurements for 84 AA-stabilized sialylated N-glycan masses between individual patient normal and tumor cores were calculated then averaged for the 4 biomarker-designations. Positive values represent enrichment in tumor tissue. Shown too are the average tumor AUP intensities for each sialylated N-glycan in each category.

Annotation of Reported N-glycan Structures by CID Fragmentation

timsTOF Flex MALDI-QTOF MS/MS Settings

Transfer
Funnel 1 RF: 425.0 Vpp
Funnel 2: RF: 500.0 Vpp
Multipole RF: 550.0 Vpp
Deflection Δ: 70.0 V
MALDI Plate Offset: 50.0 V

MS Settings
Scan Begin: 50 *m/z*
Scan End: 2500 *m/z*
Ion Polarity: Positive
Scan Mode: MS/MS
Laser Shots: 400
Laser Power: 36%
Frequency: 10000 Hz

Pre-TOF Focus
Transfer Time: 72.0 μs
Pre-Pulse Storage: 12.0 μs

Collision Cell
Collision Energy: 25.0 eV
Collison RF: 1500.0 Vpp

Quadrupole
Ion Energy: 4.0 eV
Low Mass: 150 *m/z*

CID Settings
Isolation Width: 10.00 *m/z*
Collision Energy: 150.00 eV

Notes on CID

CID fragmentation of 23 N-glycan base structures representing a mix of both core and terminally-fucosylated bi-, tri- and tetra-antennary configurations with and without bisecting GlcNAc residues. Where applicable EndoF3 was used to trim higher-mass core-fucosylated N-glycans to improve CID performance

CID Legend

X,Y,Z ions
B,C ions
Intermixed X,Y,Z/B,C ions
Precursor ions

Example MS/MS

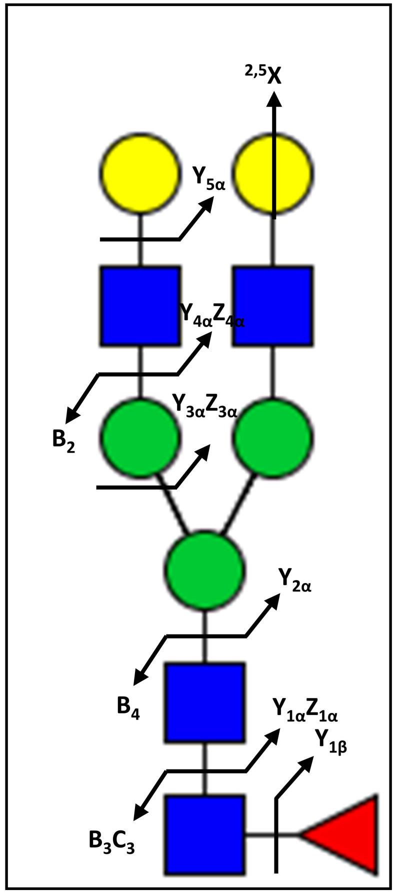

Supplemental Figure 4: Annotation of Reported N-glycan Structures by CID Fragmentation

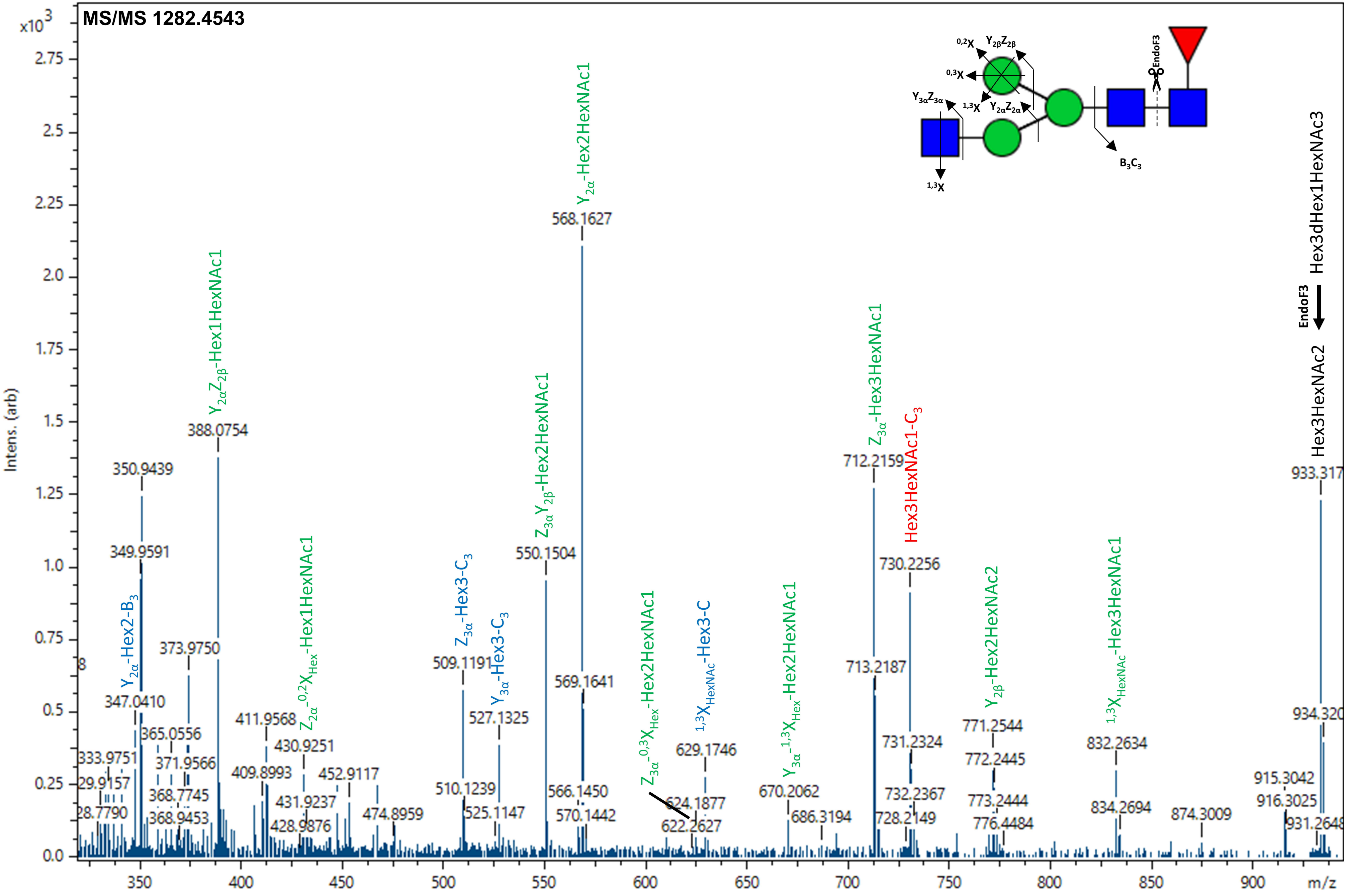
MS/MS 1282.4543
x10^3
Intens. (arb)
m/z
EndoF3
Hex3dHex1HexNAc3
Hex3HexNAc2
Hex3HexNAc1-C3
B3C3
Y2αZ2α
Y3αZ3α
Y2βZ2β
568.1627
388.0754
350.9439
349.9591
550.1504
712.2159
730.2256
933.317
373.9750
509.1191
569.1641
713.2187
347.0410
527.1325
629.1746
771.2544
832.2634
670.2062
934.320
915.3042
916.3025
874.3009
834.2694
686.3194
622.2627
624.1877
566.1450
570.1442
525.1147
510.1239
474.8959
452.9117
431.9237
430.9251
428.9876
411.9568
409.8993
368.9453
368.7745
371.9566
365.0556
333.9751
731.2324
732.2367
728.2149
772.2445
773.2444
776.4484
931.2648

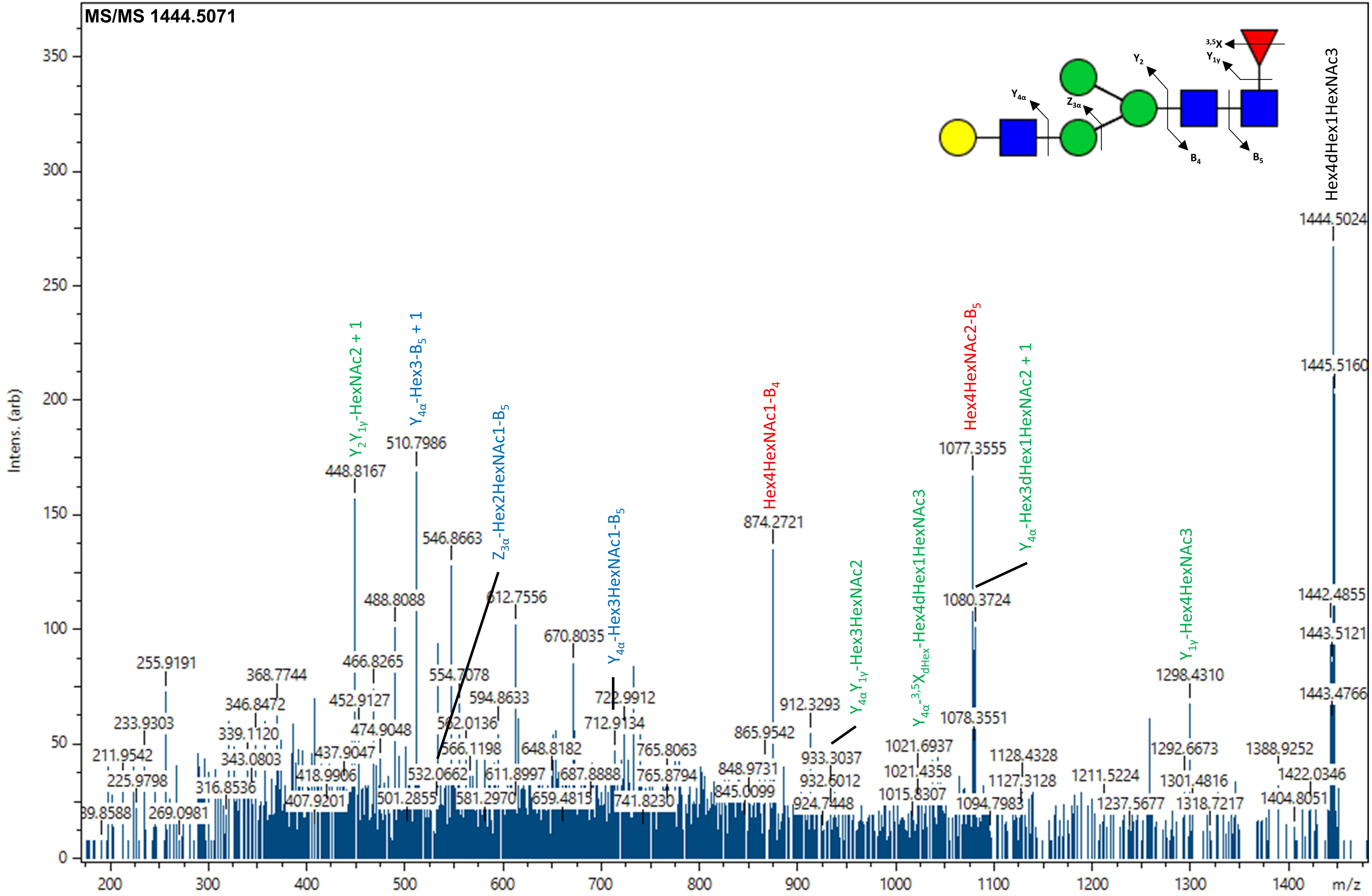

MS/MS 1444.5071
Intens. (arb)
m/z
Hex4dHex1HexNAc3
Y2Y1γ-HexNAc2 + 1
Y4α-Hex3-B5 + 1
Z3α-Hex2HexNAc1-B5
Y4α-Hex3HexNAc1-B5
Hex4HexNAc1-B4
Hex4HexNAc2-B5
Y4α-Hex3dHex1HexNAc2 + 1
Y4αY1γ-Hex3HexNAc2
Y4α-3,5XdHex-Hex4dHex1HexNAc3
Y1γ-Hex4HexNAc3
Y4α
Z3α
Y2
Y1γ
3,5X
B4
B5

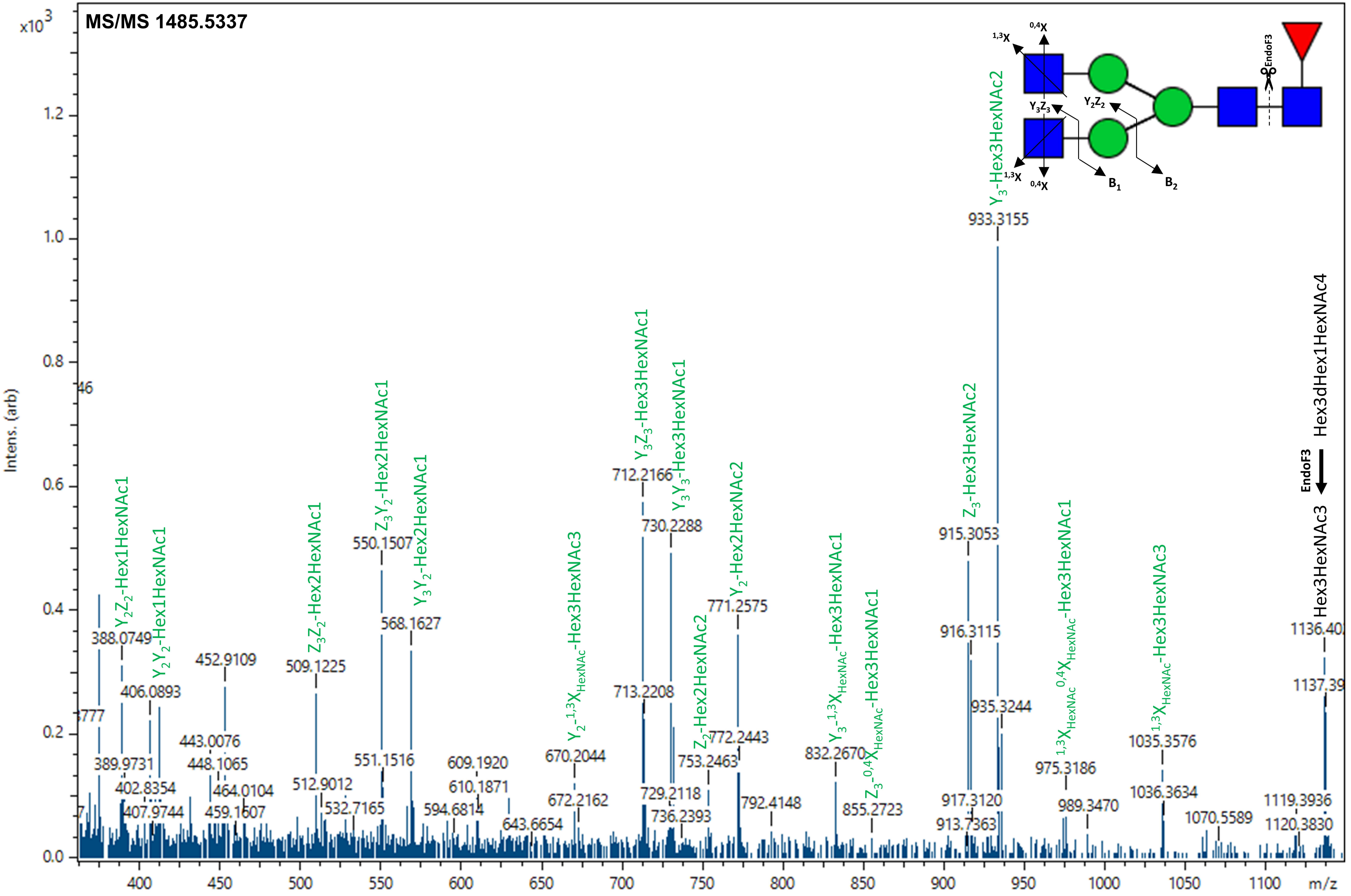
MS/MS 1485.5337
x10^3
Intens. (arb)
m/z
Y3-Hex3HexNAc2
933.3155
Y3Z3-Hex3HexNAc1
712.2166
Y3Y3-Hex3HexNAc1
730.2288
Z3Y2-Hex2HexNAc1
550.1507
Y3Y2-Hex2HexNAc1
568.1627
Z3Z2-Hex2HexNAc1
509.1225
Y2Z2-Hex1HexNAc1
388.0749
Y2Y2-Hex1HexNAc1
406.0893
452.9109
443.0076
448.1065
389.9731
402.8354
407.9744
464.0104
459.1607
512.9012
532.7165
551.1516
594.6814
609.1920
610.1871
643.6654
Y2-1,3XHexNAc-Hex3HexNAc3
670.2044
672.2162
713.2208
729.2118
736.2393
Z2-Hex2HexNAc2
753.2463
Y2-Hex2HexNAc2
771.2575
772.2443
792.4148
Y3-1,3XHexNAc-Hex3HexNAc1
832.2670
Z3-0,4XHexNAc-Hex3HexNAc1
855.2723
Z3-Hex3HexNAc2
915.3053
916.3115
917.3120
913.7363
935.3244
1,3XHexNAc0,4XHexNAc-Hex3HexNAc1
975.3186
989.3470
1,3XHexNAc-Hex3HexNAc3
1035.3576
1036.3634
1070.5589
1119.3936
1120.3830
1136.40
1137.39
Hex3dHex1HexNAc4
EndoF3
Hex3HexNAc3
1,3X
0,4X
Y3Z3
Y2Z2
B1
B2
EndoF3

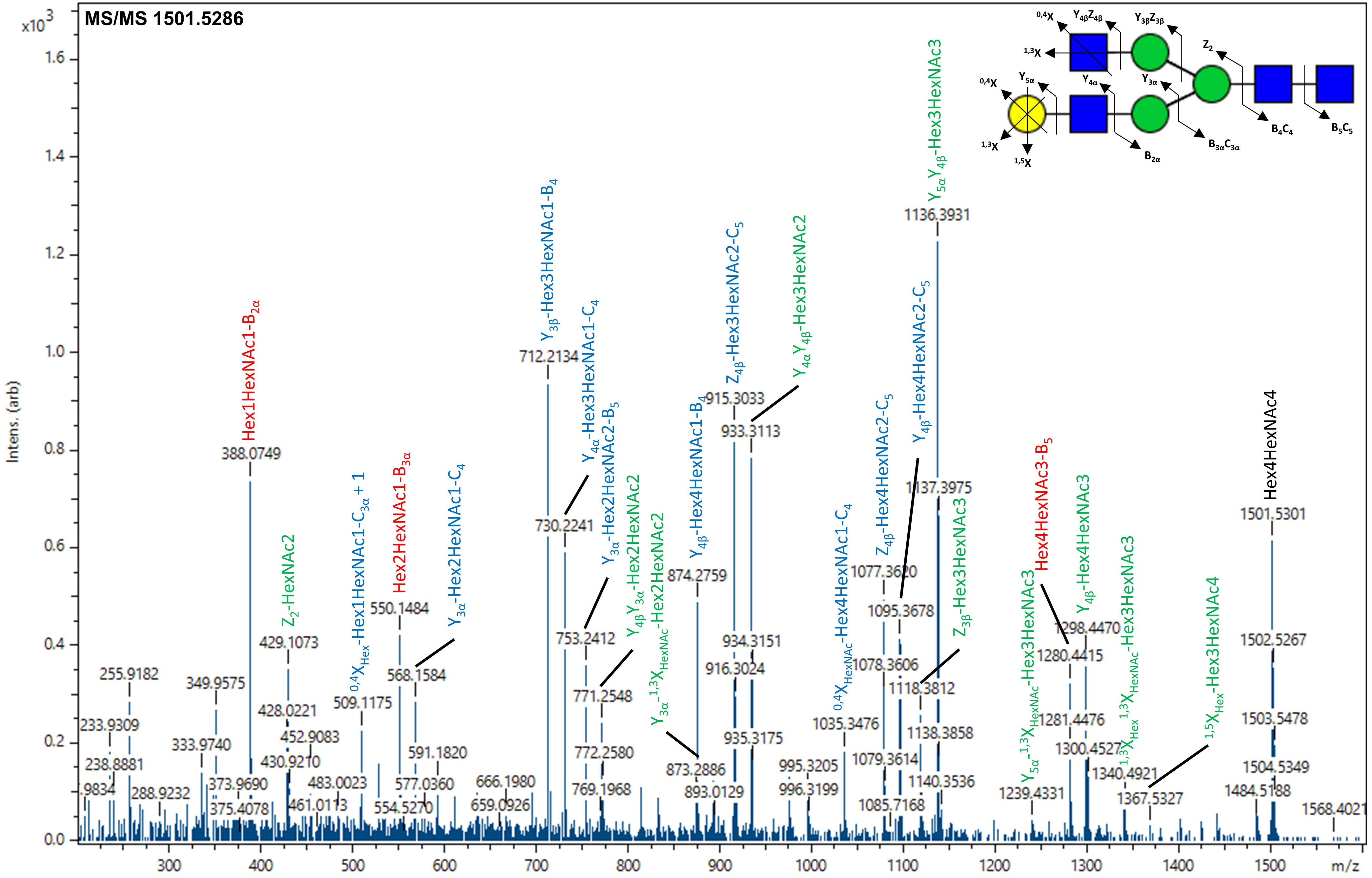

MS/MS 1501.5286
x10^3
Intens. (arb)
m/z
Hex1HexNAc1-B2α
388.0749
Z2-HexNAc2
429.1073
Hex2HexNAc1-B3α
550.1484
Y3α-Hex2HexNAc1-C4
568.1584
Y3β-Hex3HexNAc1-B4
712.2134
Y4α-Hex3HexNAc1-C4
730.2241
Y3α-Hex2HexNAc2-B5
753.2412
Y4βY3α-Hex2HexNAc2
771.2548
Y4β-Hex4HexNAc1-B4
874.2759
Z4β-Hex3HexNAc2-C5
915.3033
Y4αY4β-Hex3HexNAc2
933.3113
Z4β-Hex4HexNAc2-C5
1077.3620
Y4β-Hex4HexNAc2-C5
1095.3678
Y5αY4β-Hex3HexNAc3
1136.3931
1137.3975
Z3β-Hex3HexNAc3
1118.3812
Hex4HexNAc3-B5
1280.4415
Y4β-Hex4HexNAc3
1298.4470
Hex4HexNAc4
1501.5301
1502.5267
1503.5478
1504.5349
1484.5188
1568.4021
1239.4331
1367.5327
1340.4921
1300.4527
1281.4476
1138.3858
1140.3536
1085.7168
1079.3614
1078.3606
1035.3476
996.3199
995.3205
935.3175
934.3151
916.3024
893.0129
873.2886
772.2580
769.1968
666.1980
659.0926
591.1820
577.0360
554.5270
509.1175
483.0023
461.0113
452.9083
430.9210
428.0221
375.4078
373.9690
349.9575
333.9740
288.9232
255.9182
238.8881
233.9309

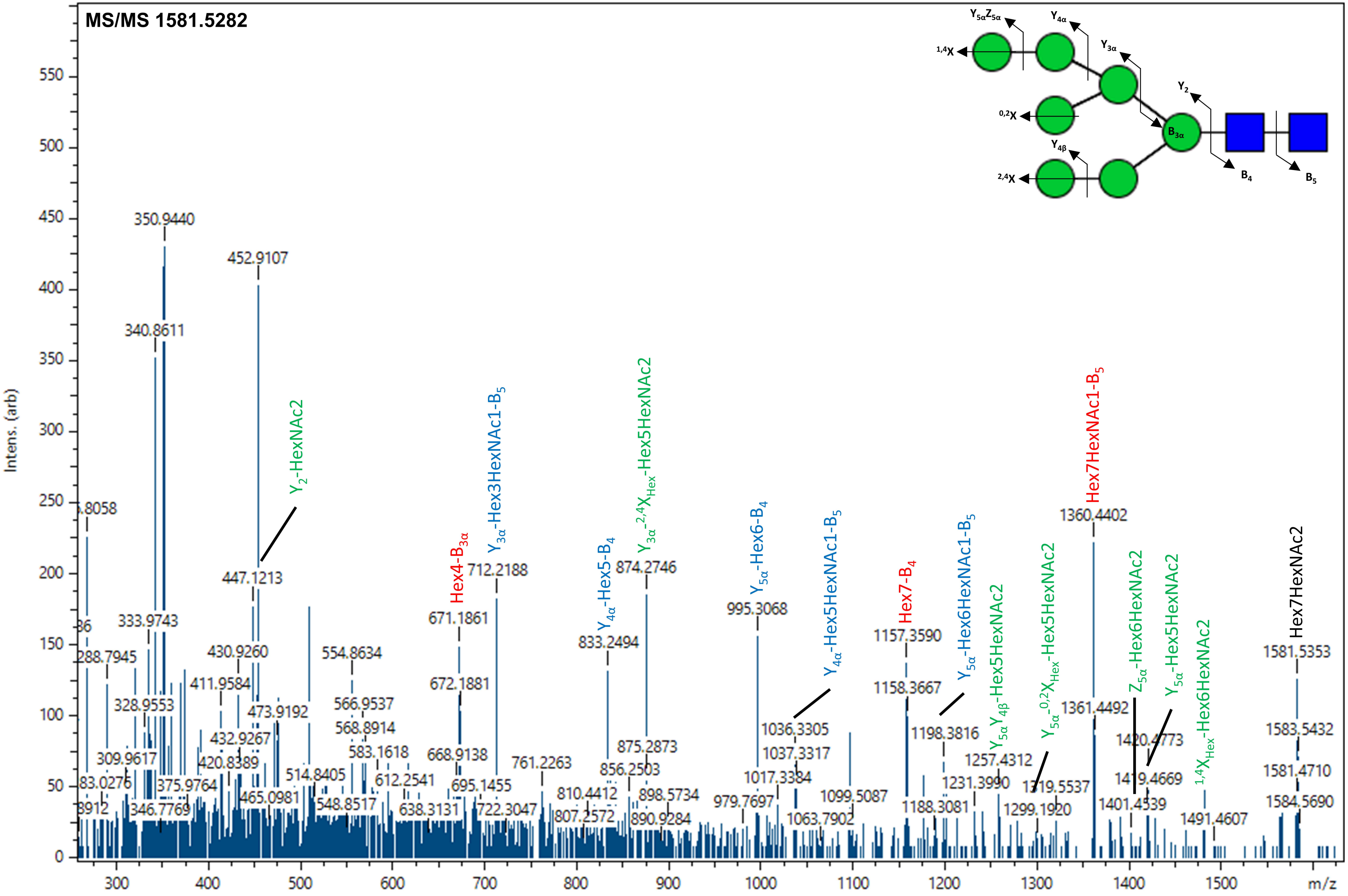

MS/MS 1581.5282
Intens. (arb)
m/z
Y2-HexNAc2
Hex4-B3α
Y3α-Hex3HexNAc1-B5
Y4α-Hex5-B4
Y3α-2,4XHex-Hex5HexNAc2
Y5α-Hex6-B4
Y4α-Hex5HexNAc1-B5
Hex7-B4
Y5α-Hex6HexNAc1-B5
Y5αY4β-Hex5HexNAc2
Y5α-0,2XHex-Hex5HexNAc2
Hex7HexNAc1-B5
Z5α-Hex6HexNAc2
Y5α-Hex5HexNAc2
1,4XHex-Hex6HexNAc2
Hex7HexNAc2
350.9440
452.9107
340.8611
447.1213
712.2188
874.2746
671.1861
833.2494
995.3068
1157.3590
1360.4402
1581.5353

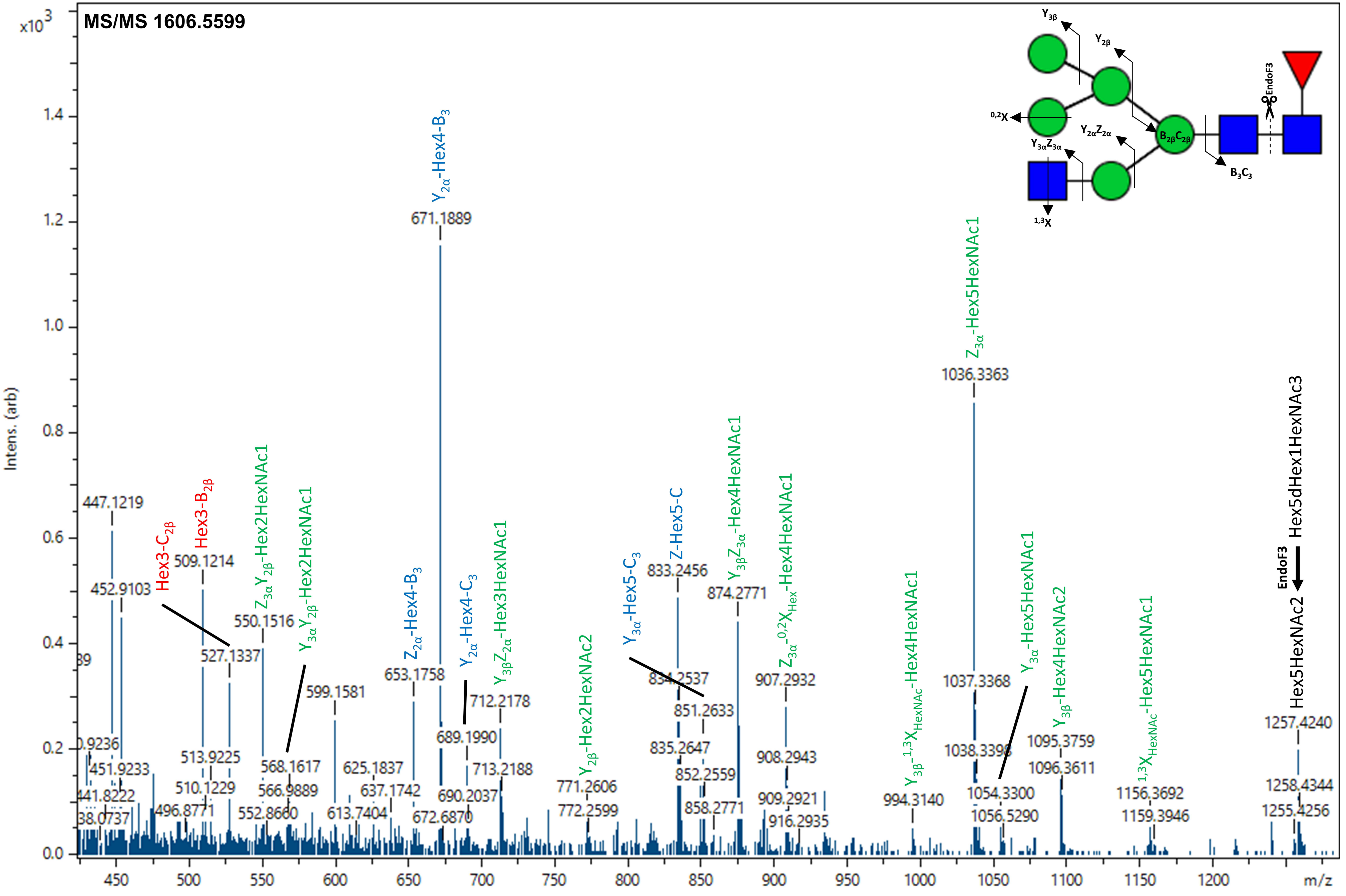

MS/MS 1606.5599
x10^3
Intens. (arb)
m/z
Y2α-Hex4-B3
671.1889
Z3α-Hex5HexNAc1
1036.3363
Hex5dHex1HexNAc3
EndoF3
Hex5HexNAc2
1257.4240
447.1219
Hex3-C2β
Hex3-B2β
509.1214
452.9103
Z3αY2β-Hex2HexNAc1
Y3αY2β-Hex2HexNAc1
550.1516
527.1337
599.1581
Z2α-Hex4-B3
653.1758
Y2α-Hex4-C3
Y3βZ2α-Hex3HexNAc1
712.2178
689.1990
Y3α-Hex5-C3
Z-Hex5-C
833.2456
Y3βZ3α-Hex4HexNAc1
874.2771
Z3α-0,2XHex-Hex4HexNAc1
907.2932
Y2β-Hex2HexNAc2
Y3β-1,3XHexNAc-Hex4HexNAc1
Y3α-Hex5HexNAc1
Y3β-Hex4HexNAc2
1,3XHexNAc-Hex5HexNAc1
1037.3368
1038.3398
1095.3759
1096.3611
1054.3300
1056.5290
994.3140
1156.3692
1159.3946
1258.4344
1255.4256
908.2943
909.2921
916.2935
851.2633
835.2647
852.2559
858.2771
771.2606
772.2599
713.2188
690.2037
672.6870
625.1837
637.1742
613.7404
568.1617
566.9889
552.8660
513.9225
510.1229
496.8771
451.9233
441.8222
Y3β
Y2β
0,2X
Y3αZ3α
Y2αZ2α
B2βC2β
B3C3
1,3X
EndoF3

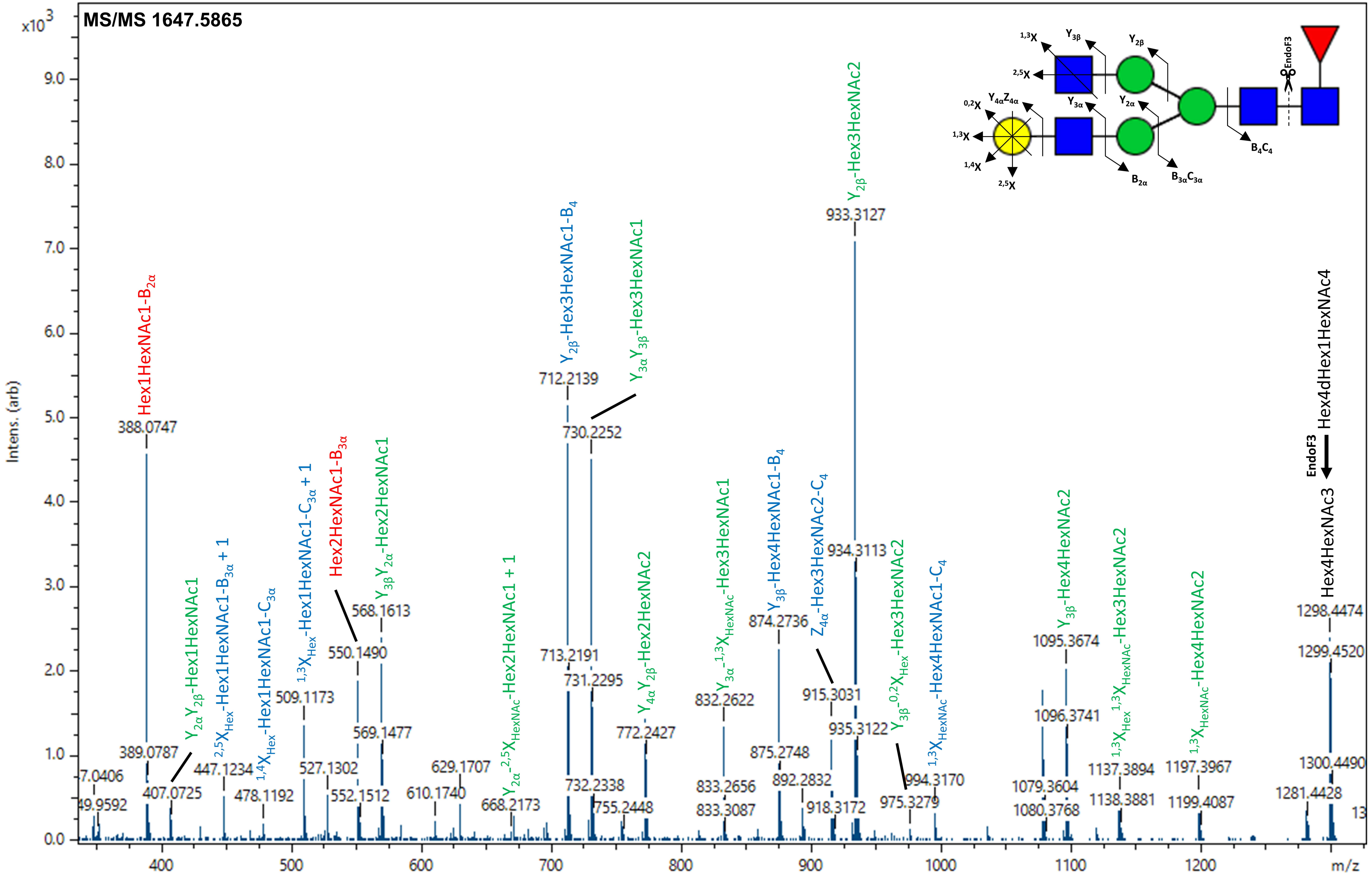

MS/MS 1647.5865
x10^3
Intens. (arb)
m/z
EndoF3
Hex4dHex1HexNAc4
Hex4HexNAc3
Hex1HexNAc1-B2α
388.0747
Y2β-Hex3HexNAc1-B4
712.2139
Y3αY3β-Hex3HexNAc1
730.2252
Y2β-Hex3HexNAc2
933.3127
934.3113
Hex2HexNAc1-B3α
550.1490
Y3βY2α-Hex2HexNAc1
568.1613
569.1477
Y3β-Hex4HexNAc1-B4
874.2736
875.2748
Z4α-Hex3HexNAc2-C4
915.3031
Y3β-0,2XHex-Hex3HexNAc2
935.3122
975.3279
1,3XHexNAc-Hex4HexNAc1-C4
994.3170
Y3β-Hex4HexNAc2
1095.3674
1096.3741
1,3XHex-1,3XHexNAc-Hex3HexNAc2
1137.3894
1138.3881
1,3XHexNAc-Hex4HexNAc2
1197.3967
1199.4087
1298.4474
1299.4520
1300.4490
1281.4428
1079.3604
1080.3768
Y2αY2β-Hex1HexNAc1
407.0725
389.0787
2,5XHex-Hex1HexNAc1-B3α + 1
447.1234
1,4XHex-Hex1HexNAc1-C3α
478.1192
1,3XHex-Hex1HexNAc1-C3α + 1
509.1173
527.1302
552.1512
610.1740
629.1707
Y2α-2,5XHexNAc-Hex2HexNAc1 + 1
668.2173
713.2191
731.2295
732.2338
755.2448
Y4αY2β-Hex2HexNAc2
772.2427
Y3α-1,3XHexNAc-Hex3HexNAc1
832.2622
833.2656
833.3087
892.2832
918.3172
EndoF3
B4C4
B2α
B3αC3α
Y3β
Y2β
Y3α
Y2α
Y4αZ4α

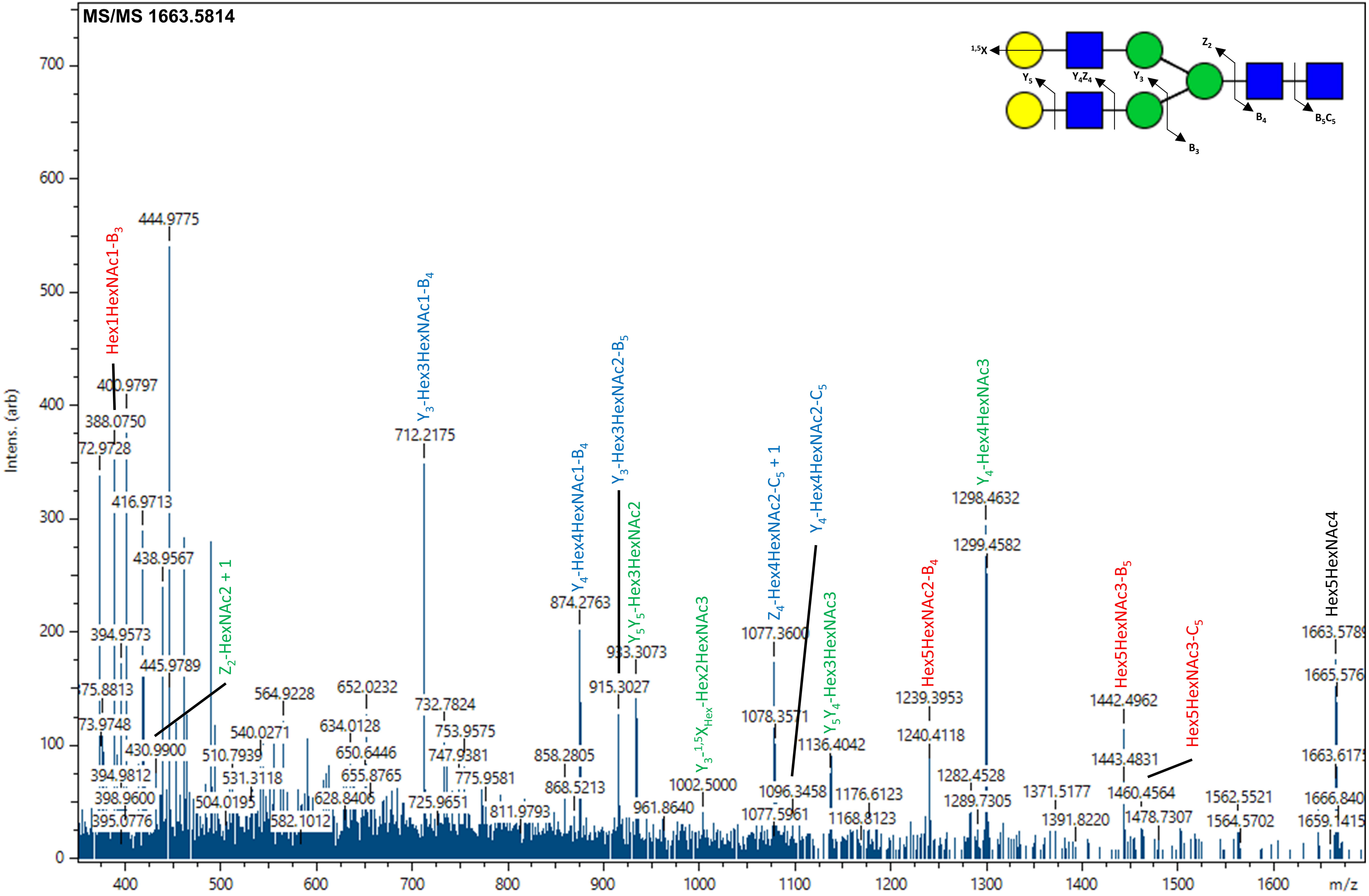

MS/MS 1663.5814
Intens. (arb)
m/z
Hex1HexNAc1-B3
444.9775
400.9797
388.0750
372.9728
416.9713
438.9567
394.9573
445.9789
375.8813
373.9748
430.9900
394.9812
398.9600
395.0776
Z2-HexNAc2 + 1
564.9228
540.0271
510.7939
531.3118
504.0195
582.1012
652.0232
634.0128
650.6446
655.8765
628.8406
Y3-Hex3HexNAc1-B4
712.2175
732.7824
753.9575
747.9381
775.9581
725.9651
811.9793
Y4-Hex4HexNAc1-B4
874.2763
858.2805
868.5213
Y3-Hex3HexNAc2-B5
Y5Y5-Hex3HexNAc2
933.3073
915.3027
961.8640
Y3-1,5XHex-Hex2HexNAc3
1002.5000
Z4-Hex4HexNAc2-C5 + 1
Y4-Hex4HexNAc2-C5
1077.3600
1078.3571
1096.3458
1077.5961
Y5Y4-Hex3HexNAc3
1136.4042
1176.6123
1168.8123
Hex5HexNAc2-B4
1239.3953
1240.4118
Y4-Hex4HexNAc3
1298.4632
1299.4582
1282.4528
1289.7305
1371.5177
1391.8220
Hex5HexNAc3-B5
1442.4962
1443.4831
1460.4564
1478.7307
Hex5HexNAc3-C5
1562.5521
1564.5702
Hex5HexNAc4
1663.5789
1665.576
1663.6175
1666.840
1659.1415

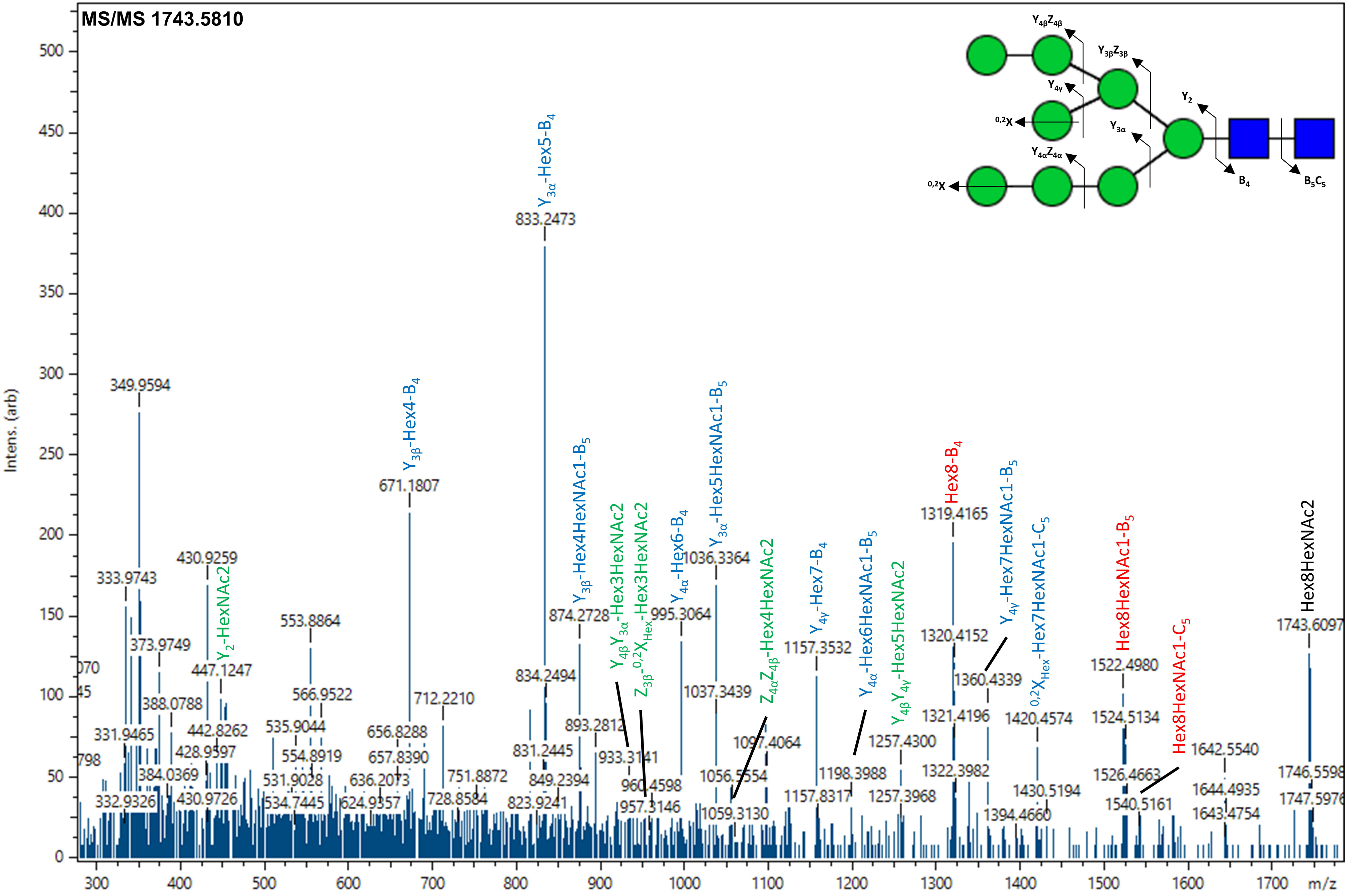

MS/MS 1743.5810
Intens. (arb)
m/z
Y3α-Hex5-B4
833.2473
Y3β-Hex4-B4
671.1807
349.9594
Hex8-B4
1319.4165
Hex8HexNAc2
1743.6097
Hex8HexNAc1-B5
1522.4980
Hex8HexNAc1-C5
Y2-HexNAc2
430.9259
Y3β-Hex4HexNAc1-B5
874.2728
Y4βY3α-Hex3HexNAc2
Z3β-0,2XHex-Hex3HexNAc2
Y4α-Hex6-B4
995.3064
Y3α-Hex5HexNAc1-B5
1036.3364
Z4αZ4β-Hex4HexNAc2
Y4γ-Hex7-B4
1157.3532
Y4α-Hex6HexNAc1-B5
Y4βY4γ-Hex5HexNAc2
Y4γ-Hex7HexNAc1-B5
0,2XHex-Hex7HexNAc1-C5

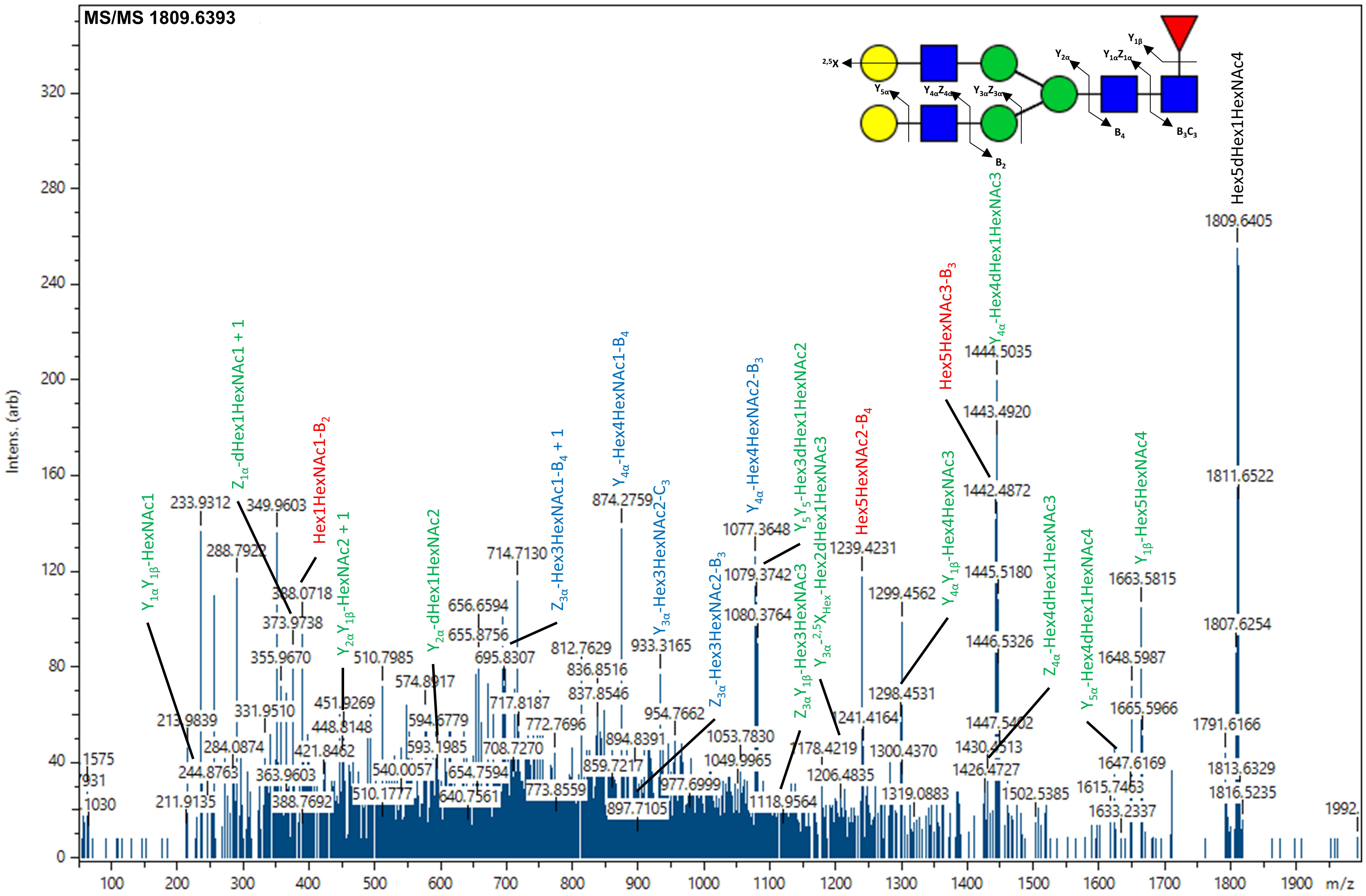
MS/MS 1809.6393
Intens. (arb)
m/z
Hex5dHex1HexNAc4
Y1αY1β-HexNAc1
Z1α-dHex1HexNAc1 + 1
Hex1HexNAc1-B2
Y2αY1β-HexNAc2 + 1
Y2α-dHex1HexNAc2
Z3α-Hex3HexNAc1-B4 + 1
Y4α-Hex4HexNAc1-B4
Y3α-Hex3HexNAc2-C3
Z3α-Hex3HexNAc2-B3
Y4α-Hex4HexNAc2-B3
Y5Y5-Hex3dHex1HexNAc2
Z3αY1β-Hex3HexNAc3
Y3α-2,5XHex-Hex2dHex1HexNAc3
Hex5HexNAc2-B4
Hex5HexNAc3-B3
Y4α-Hex4dHex1HexNAc3
Y4αY1β-Hex4HexNAc3
Z4α-Hex4dHex1HexNAc3
Y5α-Hex4dHex1HexNAc4
Y1β-Hex5HexNAc4
2,5X
Y5α
Y4αZ4α
Y3αZ3α
Y2α
Y1αZ1α
Y1β
B2
B4
B3C3
1809.6405
1811.6522
1807.6254
1791.6166
1813.6329
1816.5235
1444.5035
1443.4920
1442.4872
1445.5180
1446.5326
1447.5402
1430.4513
1426.4727
1502.5385
1663.5815
1648.5987
1665.5966
1647.6169
1615.7463
1633.2337
1239.4231
1241.4164
1299.4562
1298.4531
1300.4370
1319.0883
1178.4219
1206.4835
1077.3648
1079.3742
1080.3764
1053.7830
1049.9965
977.6999
1118.9564
874.2759
933.3165
954.7662
894.8391
859.7217
897.7105
812.7629
836.8516
837.8546
714.7130
656.6594
655.8756
695.8307
717.8187
772.7696
708.7270
773.8559
654.7594
640.7561
574.8917
594.6779
593.1985
540.0057
510.1777
510.7985
451.9269
448.8148
421.8462
233.9312
349.9603
288.7922
388.0718
373.9738
355.9670
331.9510
363.9603
388.7692
213.9839
284.0874
244.8763
211.9135
1992.
1575
931
1030

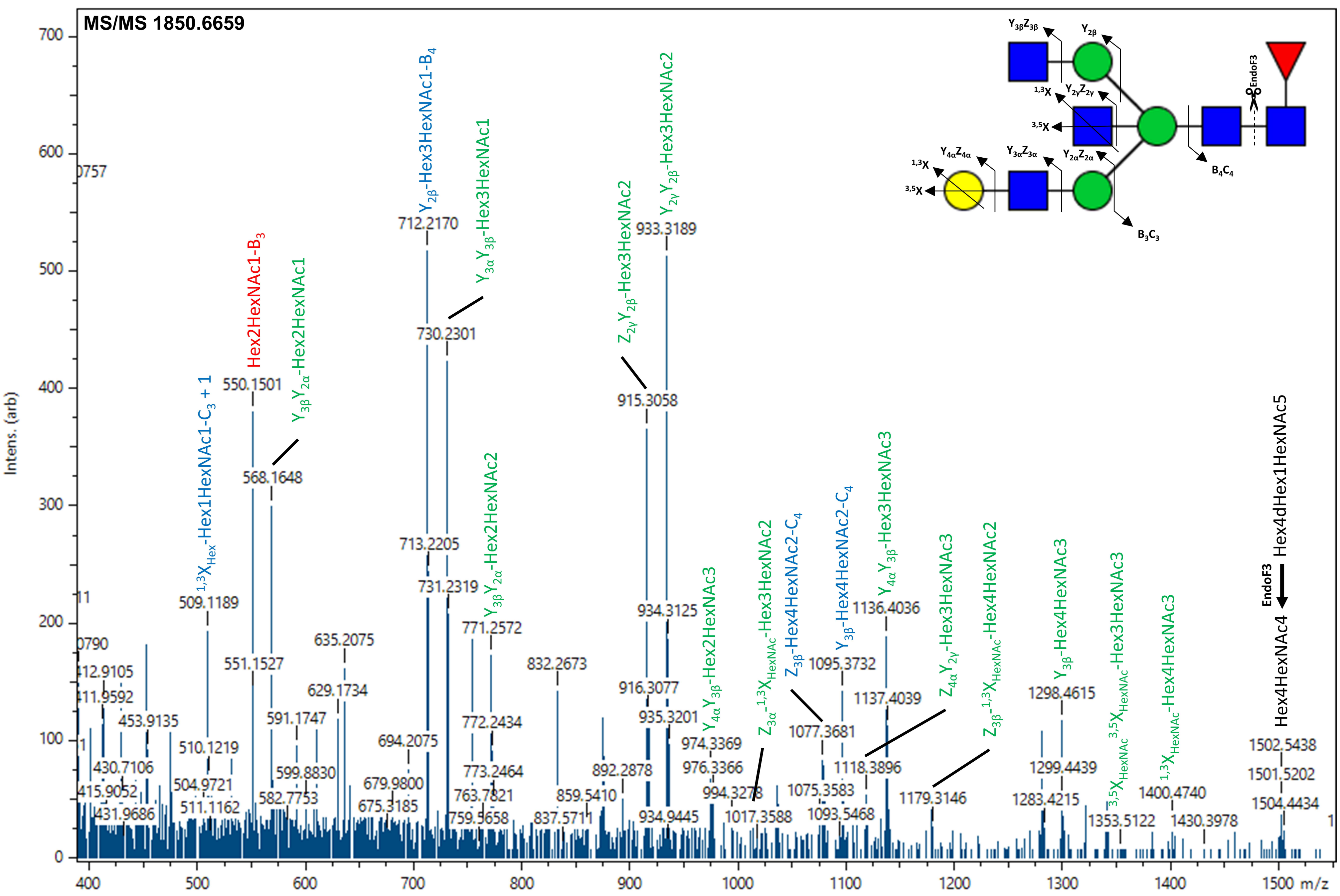

MS/MS 1850.6659
Intens. (arb)
m/z
Y2β-Hex3HexNAc1-B4
712.2170
Y3αY3β-Hex3HexNAc1
730.2301
Y2γY2β-Hex3HexNAc2
933.3189
Z2γY2β-Hex3HexNAc2
915.3058
Hex2HexNAc1-B3
550.1501
Y3βY2α-Hex2HexNAc1
568.1648
1,3XHex-Hex1HexNAc1-C3 + 1
509.1189
Y3βY2α-Hex2HexNAc2
771.2572
713.2205
731.2319
934.3125
635.2075
832.2673
Y4αY3β-Hex2HexNAc3
Z3α-1,3XHexNAc-Hex3HexNAc2
Z3β-Hex4HexNAc2-C4
Y3β-Hex4HexNAc2-C4
Y4αY3β-Hex3HexNAc3
Z4αY2γ-Hex3HexNAc3
Z3β-1,3XHexNAc-Hex4HexNAc2
Y3β-Hex4HexNAc3
3,5XHexNAc-Hex3HexNAc3
1,3XHexNAc-Hex4HexNAc3
Hex4HexNAc4
EndoF3
Hex4dHex1HexNAc5
1136.4036
1095.3732
1077.3681
1118.3896
1179.3146
1298.4615
1502.5438
Y3βZ3β
Y2β
1,3X
Y2γZ2γ
3,5X
EndoF3
Y4αZ4α
Y3αZ3α
Y2αZ2α
B4C4
B3C3

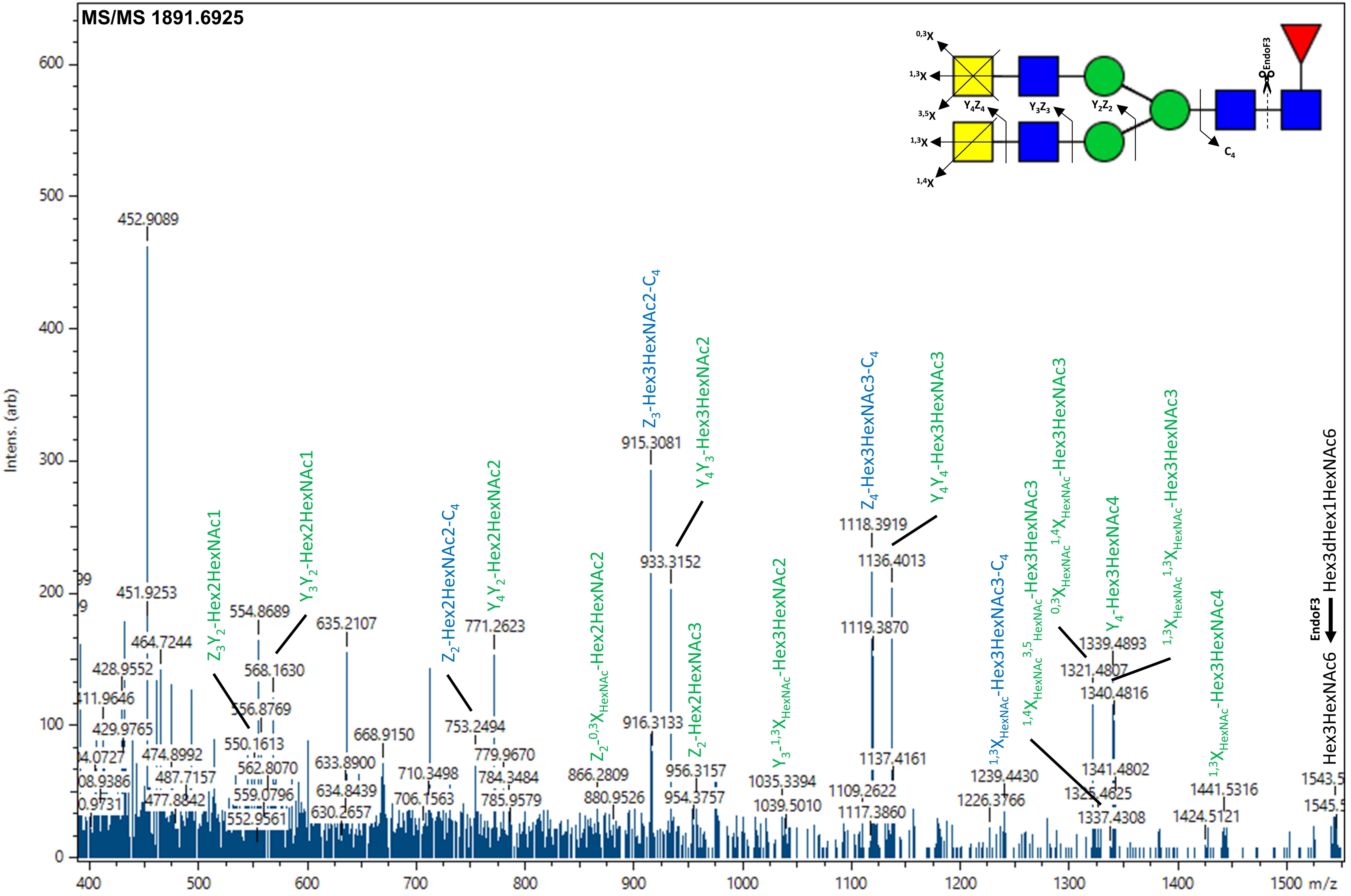

MS/MS 1891.6925
Intens. (arb)
m/z
452.9089
915.3081
933.3152
1118.3919
1136.4013
1119.3870
1137.4161
1339.4893
1321.4807
1340.4816
1341.4802
771.2623
635.2107
554.8689
568.1630
753.2494
Z3-Hex3HexNAc2-C4
Y4Y3-Hex3HexNAc2
Z4-Hex3HexNAc3-C4
Y4Y4-Hex3HexNAc3
Z3Y2-Hex2HexNAc1
Y3Y2-Hex2HexNAc1
Z2-Hex2HexNAc2-C4
Y4Y2-Hex2HexNAc2
Z2-Hex2HexNAc3
Y4-Hex3HexNAc4
EndoF3
Hex3dHex1HexNAc6
Hex3HexNAc6
C4

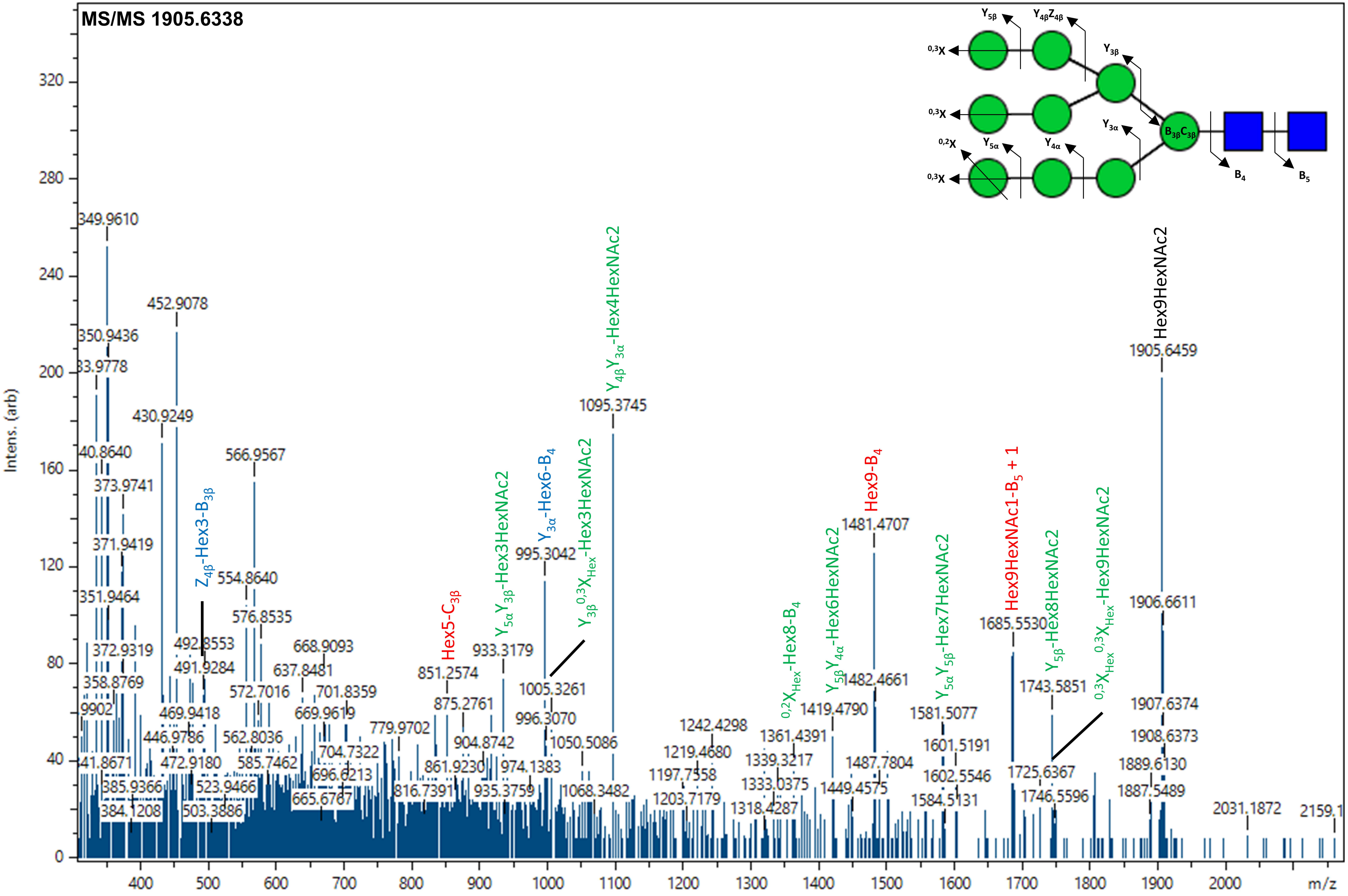
MS/MS 1905.6338
Intens. (arb)
m/z
Hex9HexNAc2
1905.6459
1095.3745
Hex9-B4
1481.4707
Hex9HexNAc1-B5 + 1
1685.5530
452.9078
349.9610
430.9249
566.9567
995.3042
Hex5-C3β
851.2574
933.3179
1005.3261
1906.6611
1907.6374
1908.6373
1889.6130
1887.5489
2031.1872
1743.5851
1725.6367
1746.5596
1581.5077
1601.5191
1602.5546
1584.5131
1419.4790
1482.4661
1487.7804
1449.4575
1361.4391
1339.3217
1333.0375
1318.4287
1242.4298
1219.4680
1197.7558
1203.7179
1050.5086
1068.3482
996.3070
974.1383
935.3759
904.8742
875.2761
861.9230
816.7391
779.9702
668.9093
637.8481
701.8359
669.9619
704.7322
696.6213
665.6767
554.8640
576.8535
572.7016
562.8036
585.7462
492.8553
491.9284
469.9418
446.9786
472.9180
523.9466
503.3886
350.9436
373.9741
371.9419
351.9464
372.9319
358.8769
385.9366
384.1208

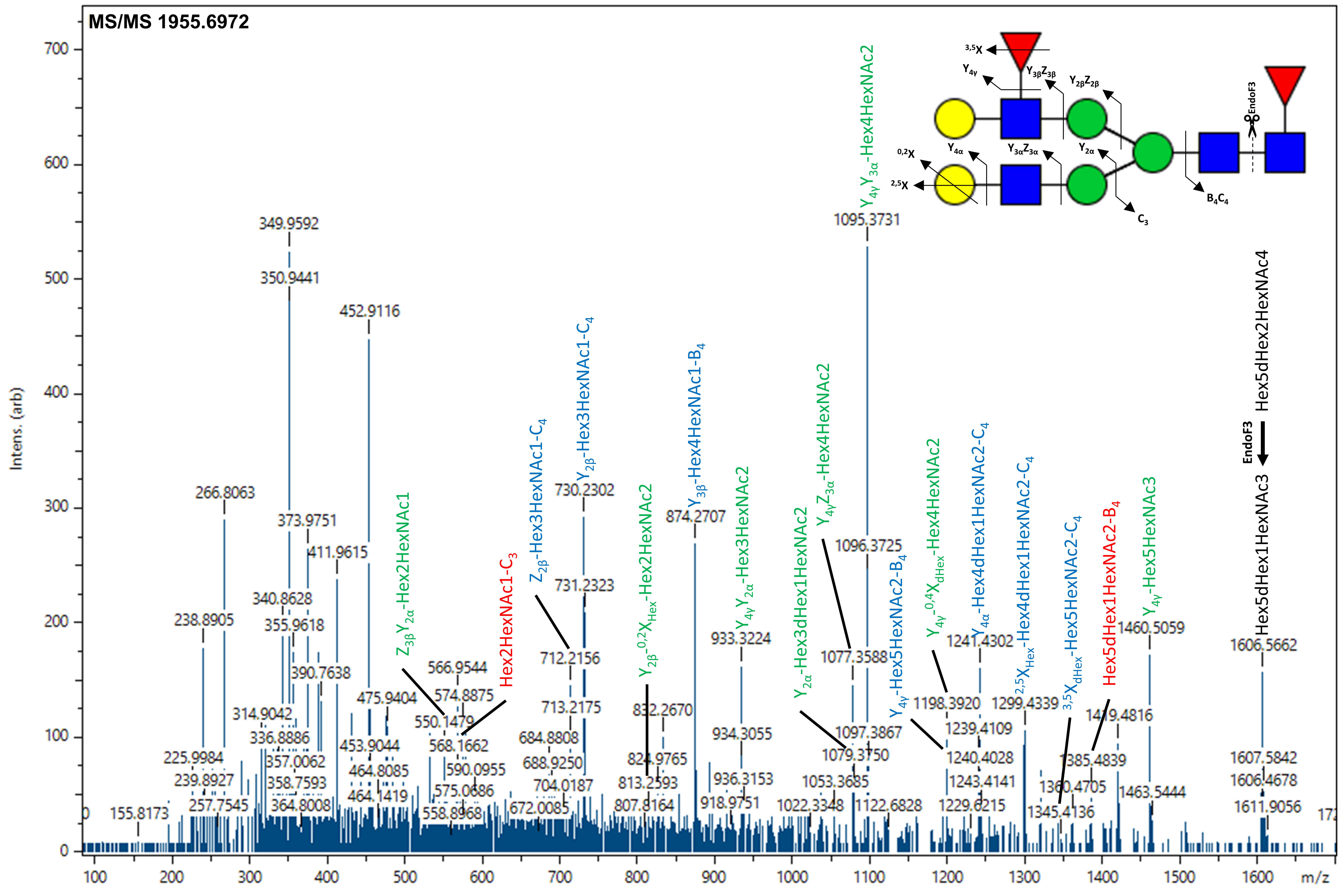
MS/MS 1955.6972
Intens. (arb)
m/z
EndoF3
Hex5dHex2HexNAc4
Hex5dHex1HexNAc3
1095.3731
349.9592
350.9441
452.9116
266.8063
373.9751
411.9615
1096.3725
874.2707
730.2302
731.2323
340.8628
355.9618
238.8905
390.7638
566.9544
574.8875
475.9404
550.1479
568.1662
712.2156
713.2175
832.2670
933.3224
934.3055
1077.3588
1097.3867
1079.3750
1241.4302
1198.3920
1239.4109
1240.4028
1299.4339
1460.5059
1606.5662
1419.4816
1385.4839
1607.5842
1606.4678
1611.9056
Hex2HexNAc1-C3
Hex5dHex1HexNAc2-B4

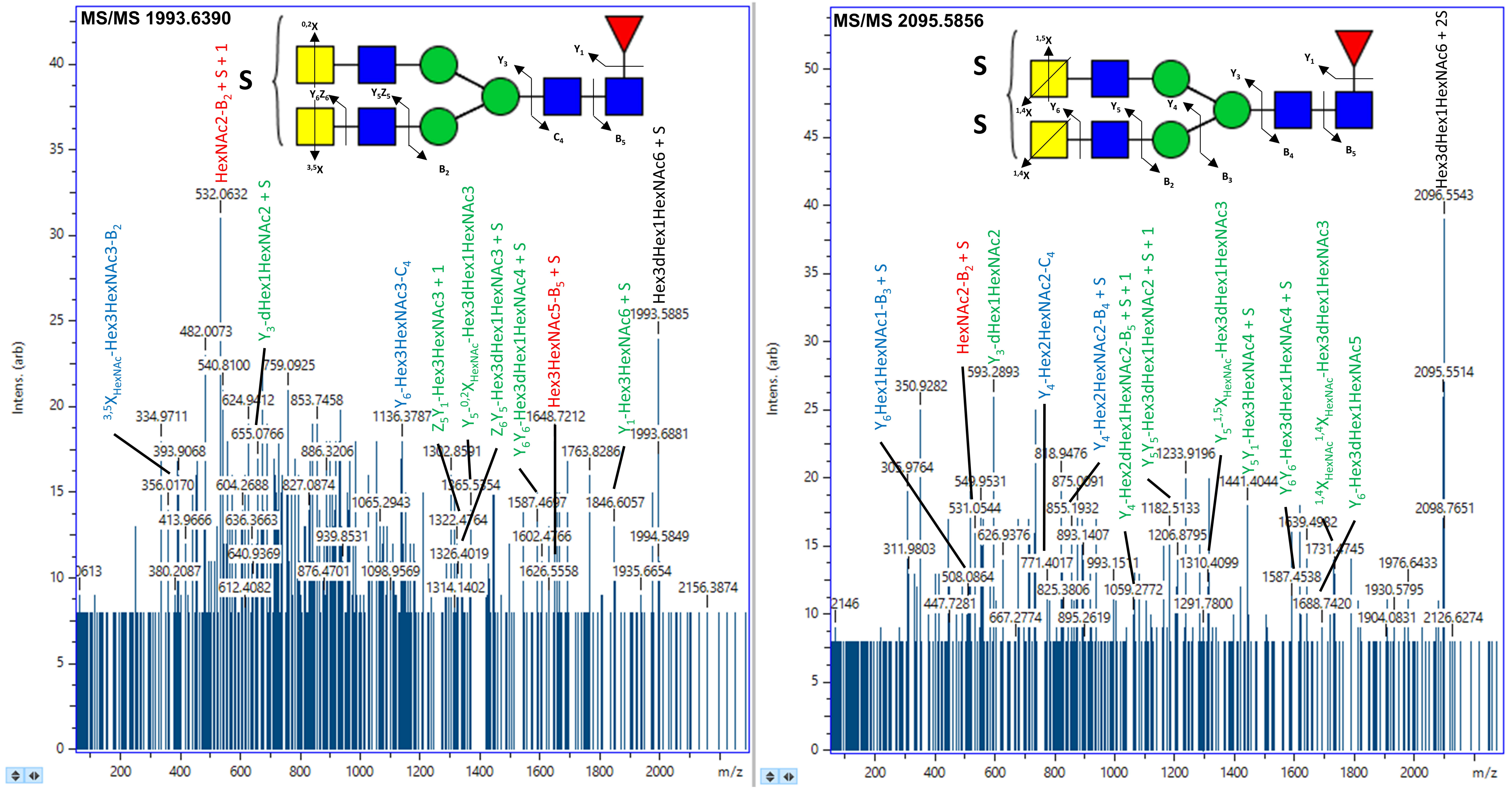

MS/MS 1993.6390
MS/MS 2095.5856
Intens. (arb)
m/z
HexNAc2-B2 + S + 1
Hex3dHex1HexNAc6 + S
Hex3dHex1HexNAc6 + 2S
HexNAc2-B2 + S
Hex3HexNAc5-B5 + S

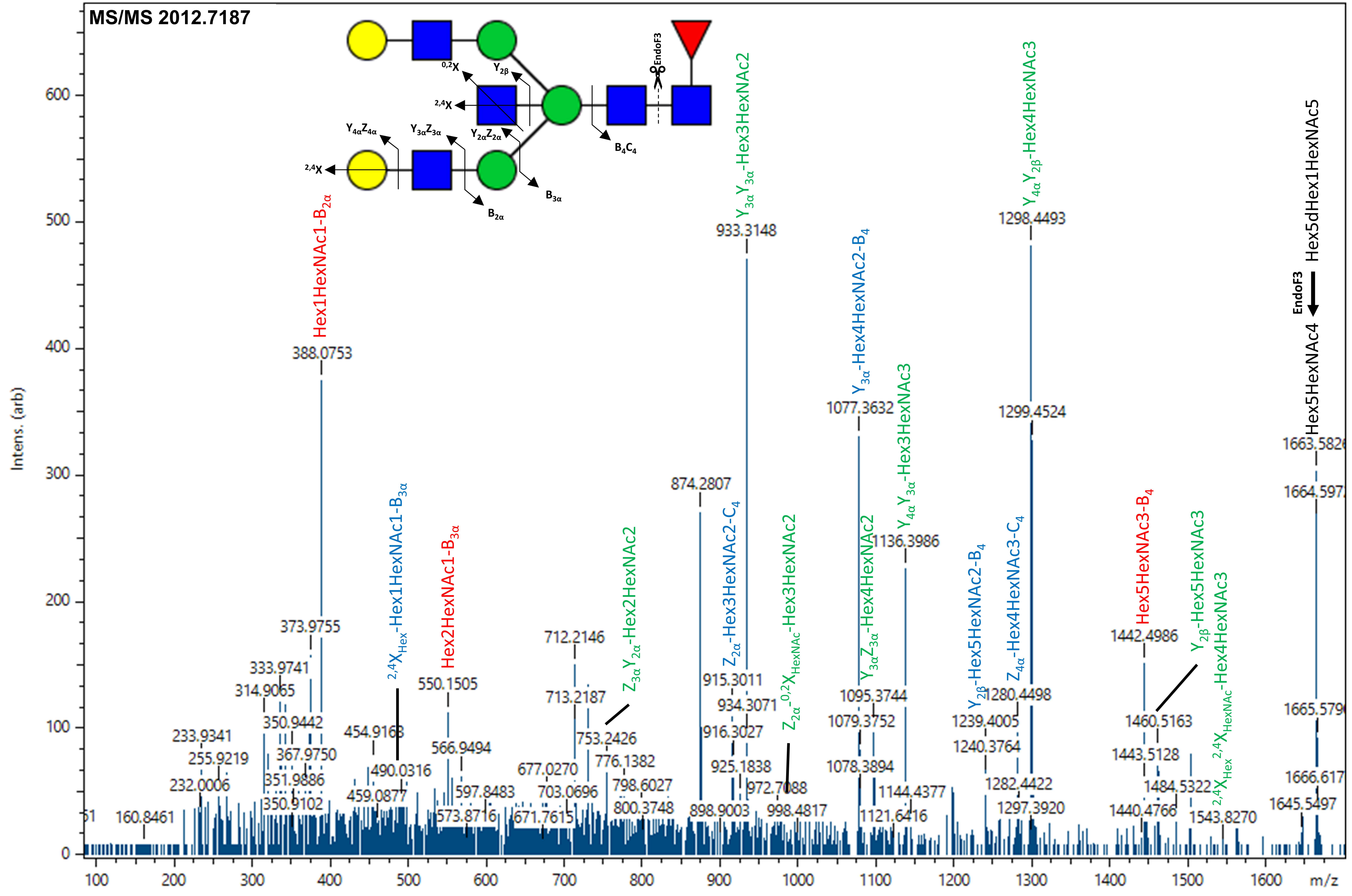
MS/MS 2012.7187
Intens. (arb)
m/z
EndoF3
Hex1HexNAc1-B2α
388.0753
Y3αY3α-Hex3HexNAc2
933.3148
Y4αY2β-Hex4HexNAc3
1298.4493
1299.4524
Y3α-Hex4HexNAc2-B4
1077.3632
Y4αY3α-Hex3HexNAc3
1136.3986
Hex5dHex1HexNAc5
Hex5HexNAc4
1663.5826
1664.5972
1665.5796
1666.617
1645.5497
874.2807
2,4XHex-Hex1HexNAc1-B3α
Hex2HexNAc1-B3α
550.1505
Z3αY2α-Hex2HexNAc2
712.2146
713.2187
Z2α-Hex3HexNAc2-C4
Z2α-0,2XHexNAc-Hex3HexNAc2
Y3αZ3α-Hex4HexNAc2
Y2β-Hex5HexNAc2-B4
Z4α-Hex4HexNAc3-C4
Hex5HexNAc3-B4
Y2β-Hex5HexNAc3
2,4XHex2,4XHexNAc-Hex4HexNAc3
1442.4986
1460.5163
1443.5128
1484.5322
1440.4766
1543.8270
373.9755
333.9741
314.9065
350.9442
367.9750
351.9886
350.9102
233.9341
255.9219
232.0006
160.8461
454.9163
490.0316
459.0877
566.9494
597.8483
573.8716
677.0270
703.0696
671.7615
753.2426
776.1382
798.6027
800.3748
915.3011
934.3071
916.3027
925.1838
972.7088
898.9003
998.4817
1095.3744
1079.3752
1078.3894
1144.4377
1121.6416
1239.4005
1280.4498
1240.3764
1282.4422
1297.3920

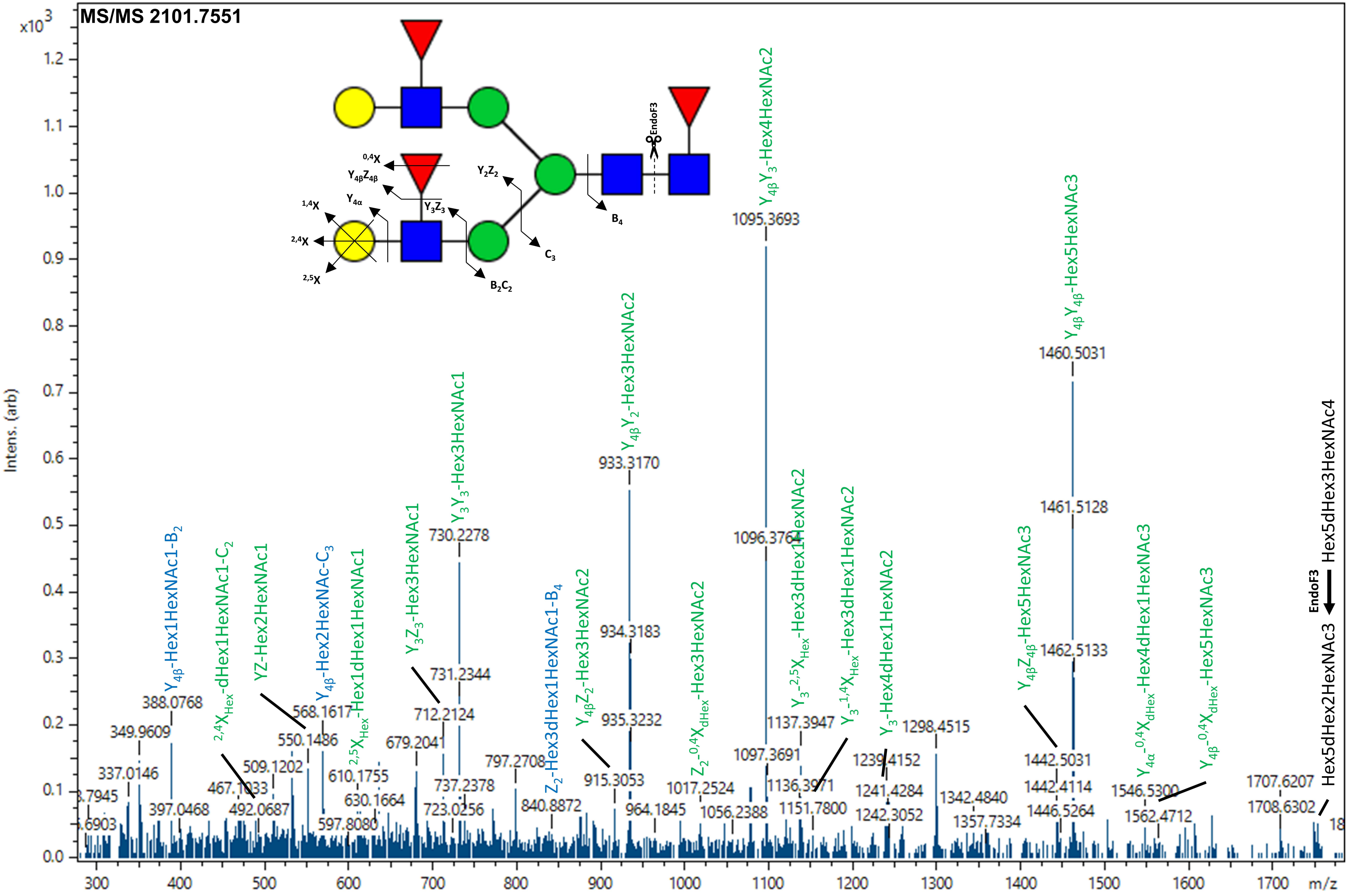

MS/MS 2101.7551
x10^3
Intens. (arb)
m/z
EndoF3
Y4βY3-Hex4HexNAc2
1095.3693
Y4βY4β-Hex5HexNAc3
1460.5031
1461.5128
1462.5133
Y4βY2-Hex3HexNAc2
933.3170
934.3183
935.3232
Y3Y3-Hex3HexNAc1
730.2278
731.2344
Hex5dHex3HexNAc4
Hex5dHex2HexNAc3

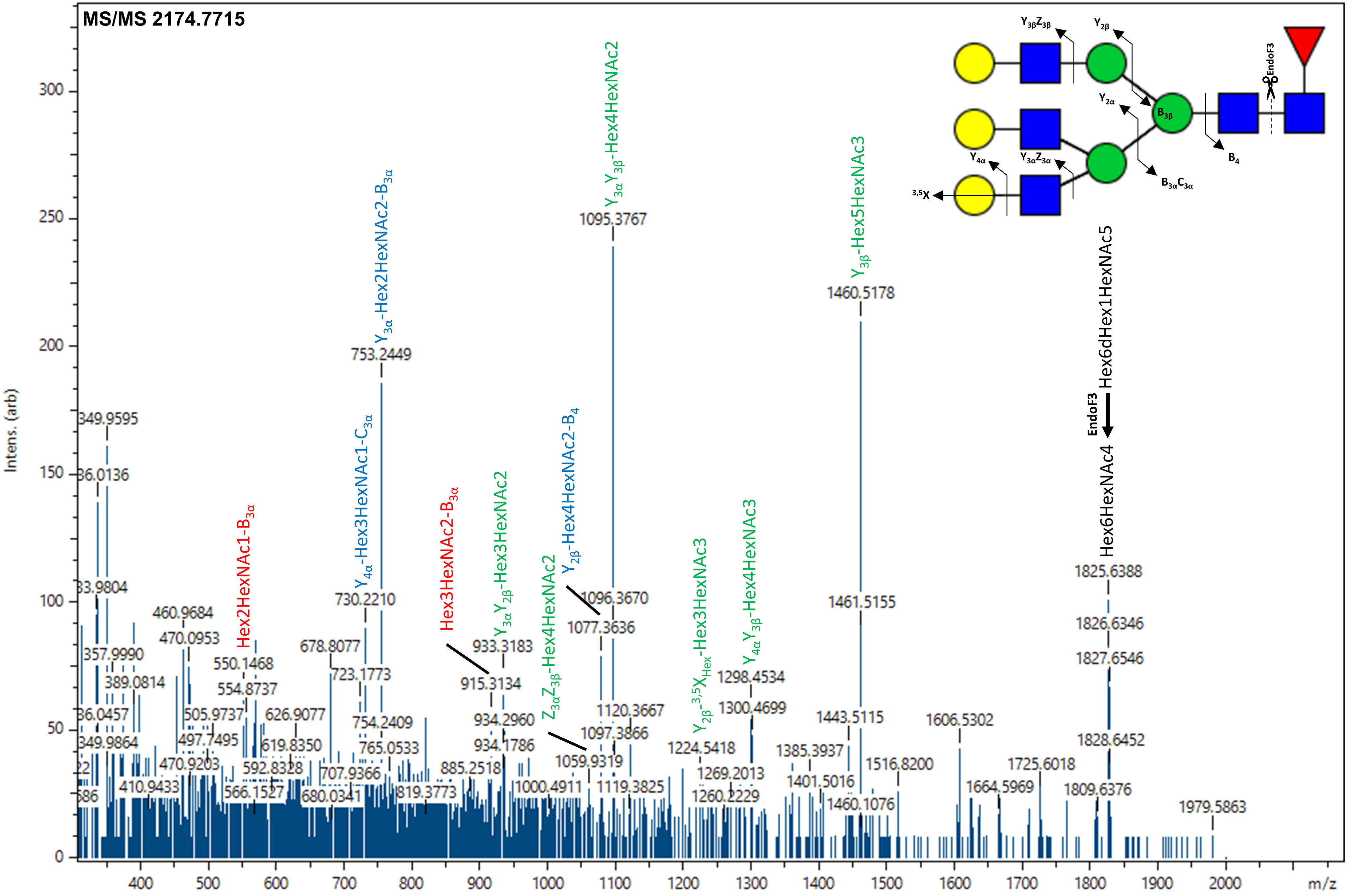
MS/MS 2174.7715
Intens. (arb)
m/z
1095.3767
1460.5178
753.2449
349.9595
1825.6388
1826.6346
1827.6546
1828.6452
1461.5155
1096.3670
1077.3636
933.3183
915.3134
730.2210
Hex6dHex1HexNAc5
EndoF3
Hex6HexNAc4
Hex2HexNAc1-B3α
Hex3HexNAc2-B3α
Y3α-Hex2HexNAc2-B3α
Y4α-Hex3HexNAc1-C3α
Y3αY3β-Hex4HexNAc2
Y3β-Hex5HexNAc3
Y2β-Hex4HexNAc2-B4
Y3αY2β-Hex3HexNAc2
Z3αZ3β-Hex4HexNAc2
Y2β-3,5XHex-Hex3HexNAc3
Y4αY3β-Hex4HexNAc3
1298.4534
1300.4699
1443.5115
1606.5302
1979.5863
1725.6018
1809.6376
1664.5969
1516.8200
1385.3937
1401.5016
1460.1076
1224.5418
1269.2013
1260.2229
1120.3667
1097.3866
1119.3825
1059.9319
1000.4911
934.2960
934.1786
885.2518
819.3773
754.2409
765.0533
723.1773
678.8077
707.9366
680.0341
626.9077
619.8350
592.8328
566.1527
550.1468
554.8737
505.9737
497.7495
470.9203
410.9433
460.9684
470.0953
357.9990
389.0814
349.9864
EndoF3
B3β
B4
B3αC3α
Y3βZ3β
Y2β
Y2α
Y4α
Y3αZ3α
3,5X

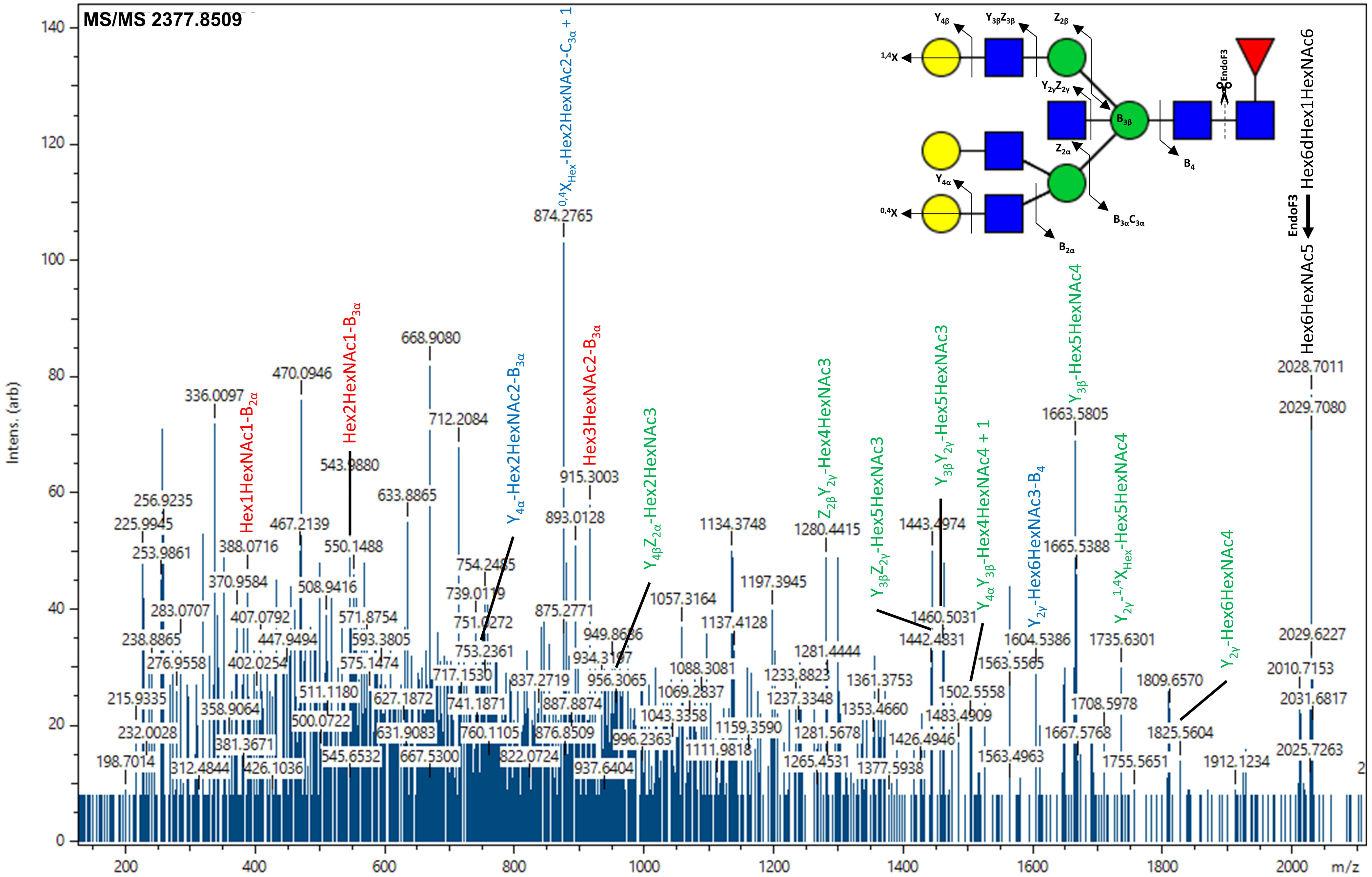
MS/MS 2377.8509
Intens. (arb)
m/z
Hex6dHex1HexNAc6
EndoF3
Hex6HexNAc5
874.2765
$^{0,4}X_{Hex}$-Hex2HexNAc2-$C_{3\alpha}$ + 1
668.9080
470.0946
336.0097
Hex1HexNAc1-$B_{2\alpha}$
Hex2HexNAc1-$B_{3\alpha}$
Hex3HexNAc2-$B_{3\alpha}$
$Y_{4\alpha}$-Hex2HexNAc2-$B_{3\alpha}$
$Y_{4\beta}Z_{2\alpha}$-Hex2HexNAc3
$Z_{2\beta}Y_{2\gamma}$-Hex4HexNAc3
$Y_{3\beta}Z_{2\gamma}$-Hex5HexNAc3
$Y_{3\beta}Y_{2\gamma}$-Hex5HexNAc3
$Y_{4\alpha}Y_{3\beta}$-Hex4HexNAc4 + 1
$Y_{2\gamma}$-Hex6HexNAc3-B_4
$Y_{3\beta}$-Hex5HexNAc4
$Y_{2\gamma}$-$^{1,4}X_{Hex}$-Hex5HexNAc4
$Y_{2\gamma}$-Hex6HexNAc4
1663.5805
2028.7011
2029.7080
915.3003
712.2084
543.9880
633.8865
1134.3748
1280.4415
1443.4974
1665.5388
1735.6301
1604.5386
1442.4831
1460.5031
1809.6570
1825.5604
1912.1234
2029.6227
2010.7153
2031.6817
2025.7263

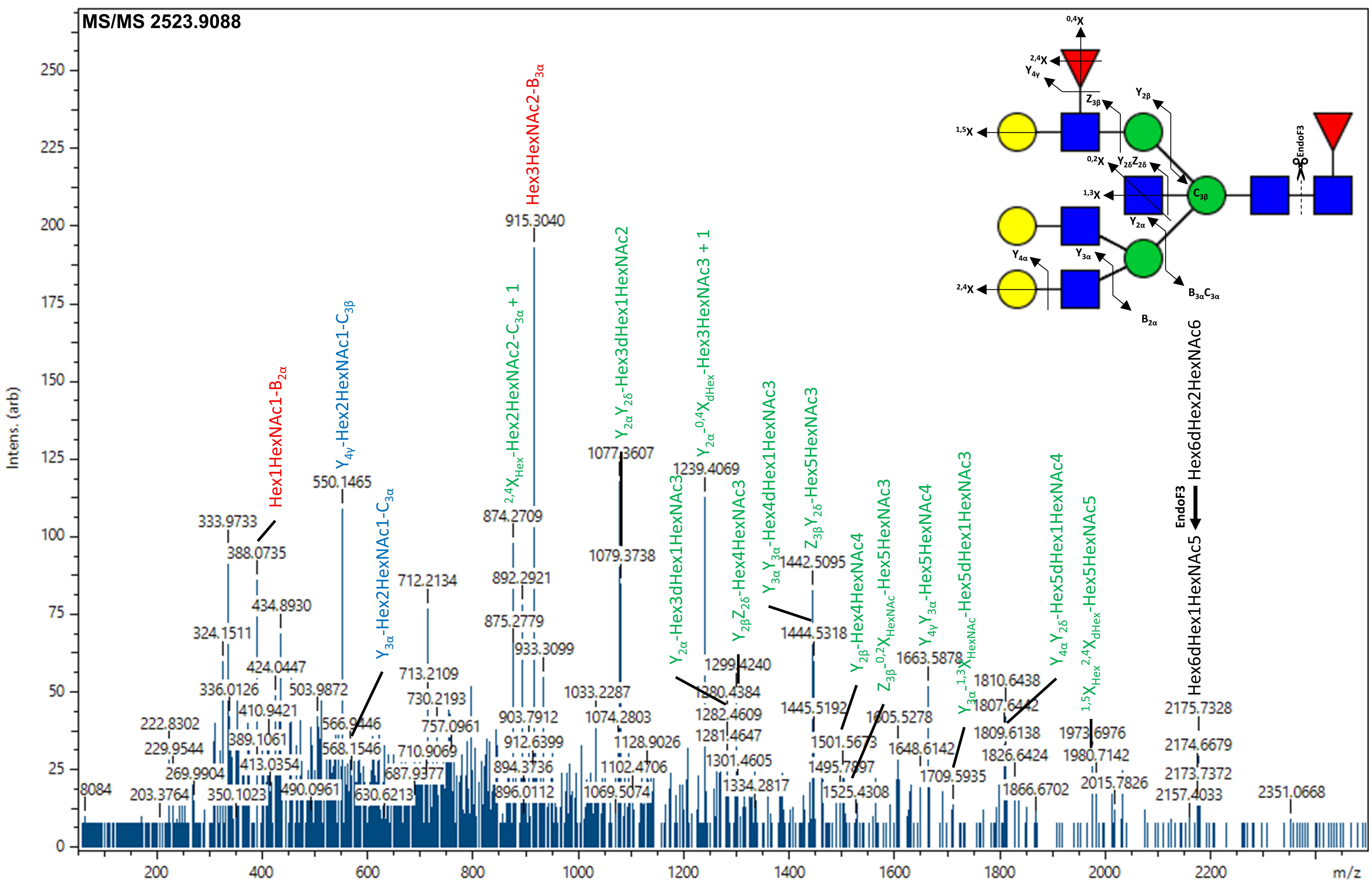
MS/MS 2523.9088
Intens. (arb)
m/z
Hex3HexNAc2-B3α
915.3040
Hex1HexNAc1-B2α
Y4γ-Hex2HexNAc1-C3β
550.1465
Y3α-Hex2HexNAc1-C3α
2,4XHex-Hex2HexNAc2-C3α + 1
Y2αY2δ-Hex3dHex1HexNAc2
Y2α-0,4XdHex-Hex3HexNAc3 + 1
Y3αY3α-Hex4dHex1HexNAc3
Z3βY2δ-Hex5HexNAc3
Y2α-Hex3dHex1HexNAc3
Y2βZ2δ-Hex4HexNAc3
Y2β-Hex4HexNAc4
Z3β-0,2XHexNAc-Hex5HexNAc3
Y4γY3α-Hex5HexNAc4
Y3α-1,3XHexNAc-Hex5dHex1HexNAc3
Y4αY2δ-Hex5dHex1HexNAc4
1,5XHex-2,4XdHex-Hex5HexNAc5
Hex6dHex2HexNAc6
EndoF3
Hex6dHex1HexNAc5
EndoF3

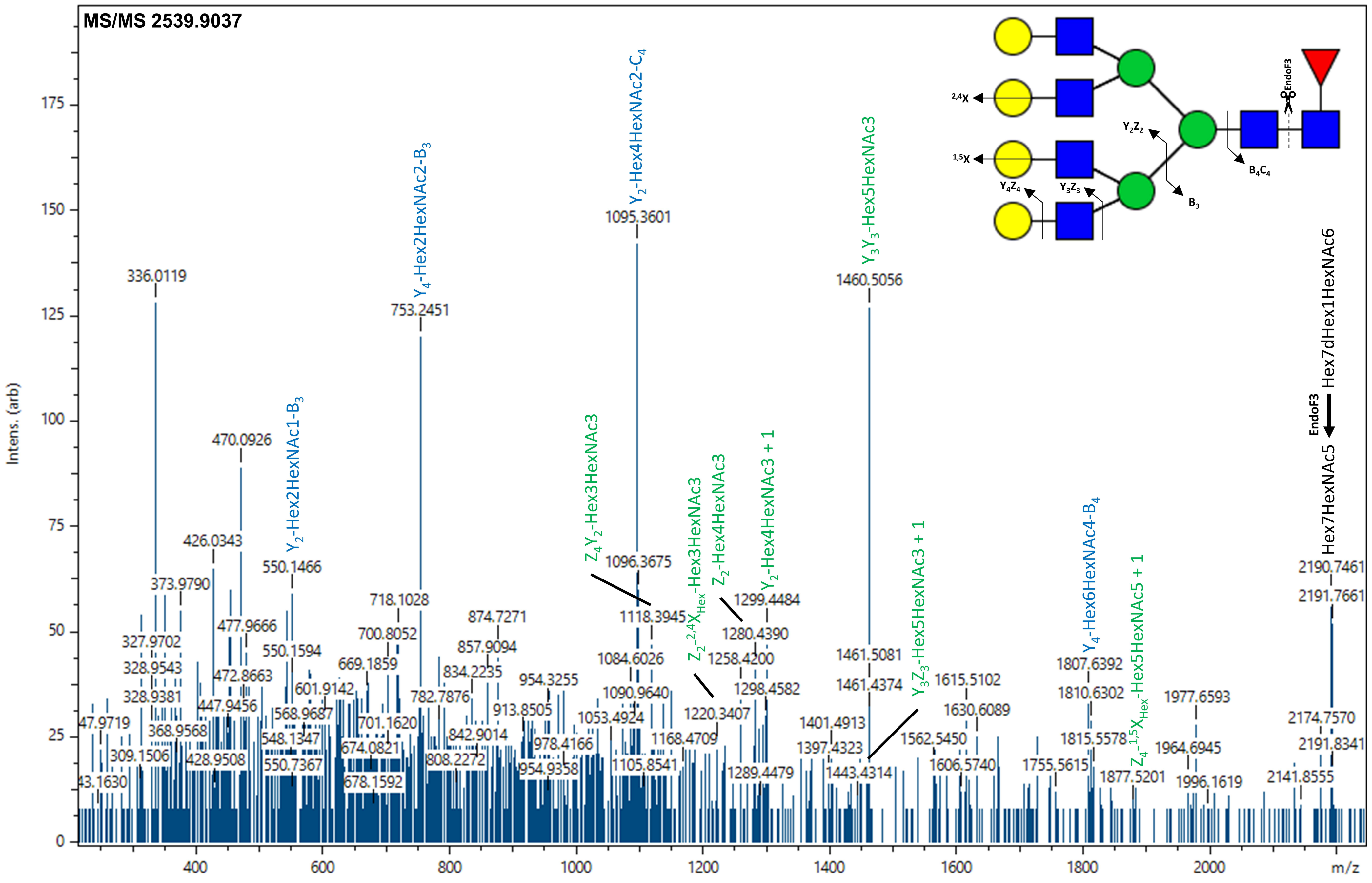
MS/MS 2539.9037
Intens. (arb)
m/z
Y2-Hex4HexNAc2-C4
1095.3601
Y4-Hex2HexNAc2-B3
753.2451
336.0119
Y3Y3-Hex5HexNAc3
1460.5056
470.0926
Y2-Hex2HexNAc1-B3
550.1466
426.0343
373.9790
Z4Y2-Hex3HexNAc3
1096.3675
1118.3945
Z2-2,4XHex-Hex3HexNAc3
Z2-Hex4HexNAc3
Y2-Hex4HexNAc3 + 1
1299.4484
1280.4390
1258.4200
1298.4582
1220.3407
Y3Z3-Hex5HexNAc3 + 1
Y4-Hex6HexNAc4-B4
Z4-1,5XHex-Hex5HexNAc5 + 1
Hex7dHex1HexNAc6
EndoF3
Hex7HexNAc5
2190.7461
2191.7661
2174.7570
2191.8341
2141.8555
1807.6392
1810.6302
1815.5578
1877.5201
1977.6593
1964.6945
1996.1619
1755.5615
1615.5102
1630.6089
1562.5450
1606.5740
1461.5081
1461.4374
1401.4913
1397.4323
1443.4314
1289.4479
1168.4709
1084.6026
1090.9640
1053.4924
1105.8541
954.3255
913.8505
978.4166
954.9358
874.7271
857.9094
834.2235
842.9014
808.2272
782.7876
718.1028
700.8052
669.1859
701.1620
674.0821
678.1592
601.9142
568.9687
548.1347
550.7367
550.1594
477.9666
472.8663
447.9456
428.9508
368.9568
327.9702
328.9543
328.9381
309.1506
47.9719
43.1630
Y2Z2
B4C4
B3
Y4Z4
Y3Z3
2,4X
1,5X
EndoF3
0
25
50
75
100
125
150
175
400
600
800
1000
1200
1400
1600
1800
2000

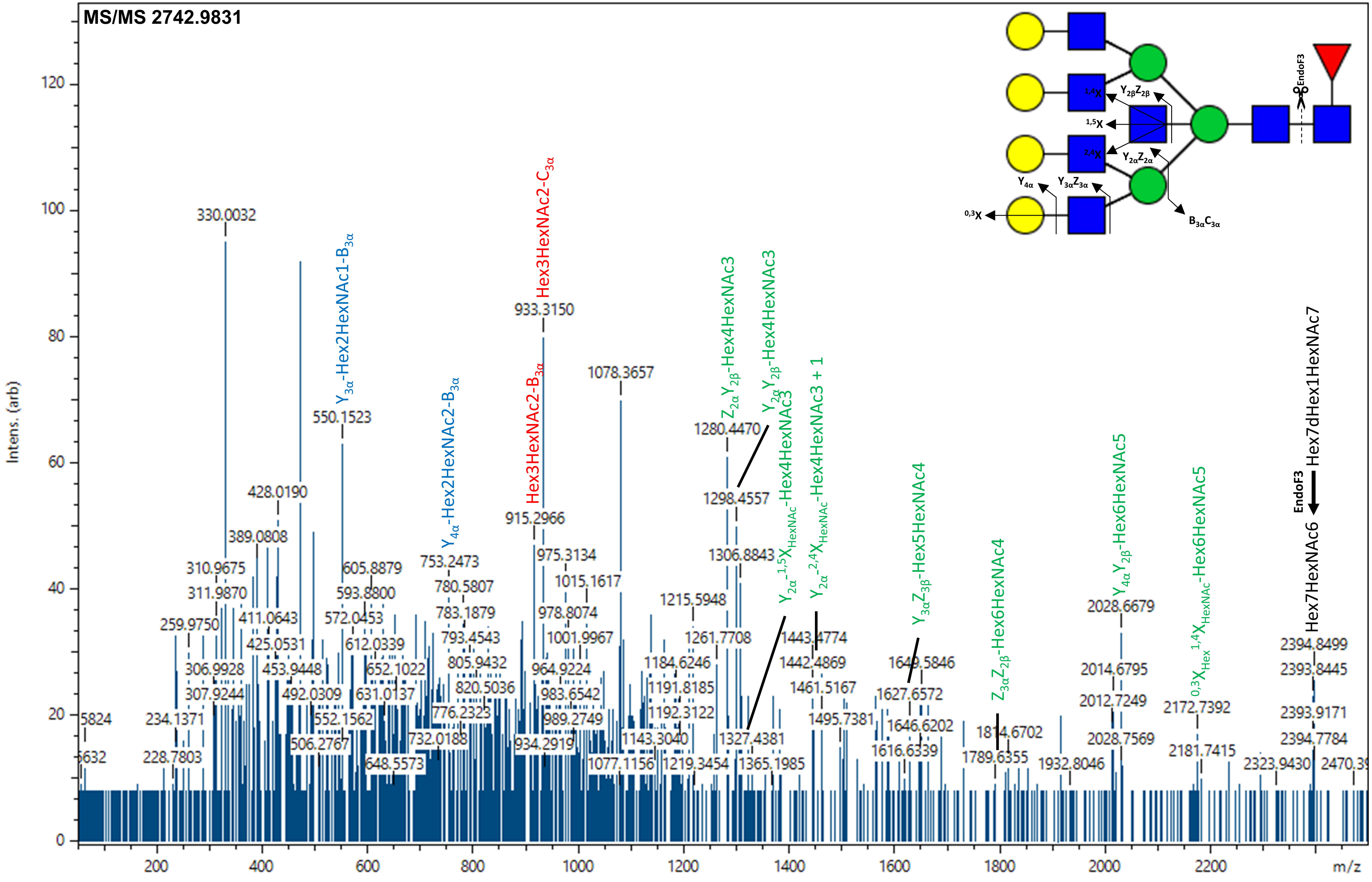
MS/MS 2742.9831
Intens. (arb)
m/z
330.0032
933.3150
1078.3657
550.1523
1280.4470
1298.4557
1306.8843
428.0190
389.0808
915.2966
Y3α-Hex2HexNAc1-B3α
Hex3HexNAc2-C3α
Hex3HexNAc2-B3α
Y4α-Hex2HexNAc2-B3α
Z2α Y2β-Hex4HexNAc3
Y2α Y2β-Hex4HexNAc3
Y2α-1,5XHexNAc-Hex4HexNAc3
Y2α-2,4XHexNAc-Hex4HexNAc3 + 1
Y3αZ3β-Hex5HexNAc4
Z3αZ2β-Hex6HexNAc4
Y4α Y2β-Hex6HexNAc5
0,3XHex 1,4XHexNAc-Hex6HexNAc5
Hex7dHex1HexNAc7
EndoF3
Hex7HexNAc6
2028.6679
2394.8499
1649.5846
1443.4774
1215.5948
Y2βZ2β
Y2αZ2α
Y3αZ3α
Y4α
B3αC3α
1,4X
1,5X
2,4X
0,3X
